# Supplementary material for: Mechanistic insights into Bcs1-mediated mitochondrial membrane translocation of the folded Rieske protein
Source: EMBO J. 2025 May 23;44(13):3720–41. doi: 10.1038/s44318-025-00459-4 (PMC12219900; doi:10.1038/s44318-025-00459-4)
Supplement: Supplementary file 1 — Appendix [file 44318_2025_459_MOESM1_ESM.pdf]

Appendix for

Mechanistic insights into Bcs1-mediated mitochondrial membrane translocation of the folded Rieske protein

Cristian Rosales-Hernandez[1], Matthias Thoms[1], Otto Berninghausen[1], Thomas Becker[1] and Roland Beckmann[1]

[1]Department of Biochemistry, Gene Center, Feodor-Lynen-Str. 25, University of Munich, 81377 Munich, Germany.

To whom correspondence should be addressed:

Roland Beckmann, Feodor-Lynen-Str. 25, 81377 Munich, Germany.

Phone: +49-89-218076900 Fax: +49-89-218076945

E-mail:beckmann@genzentrum.lmu.de

Table of Contents:

- Appendix Figures S1-S10 (incl. legends)

- Appendix Table S1: Cryo-EM data collection, refinement & validation statistics

-Appendix Table S2: Summary of Bcs1 states obtained by CryoEM.

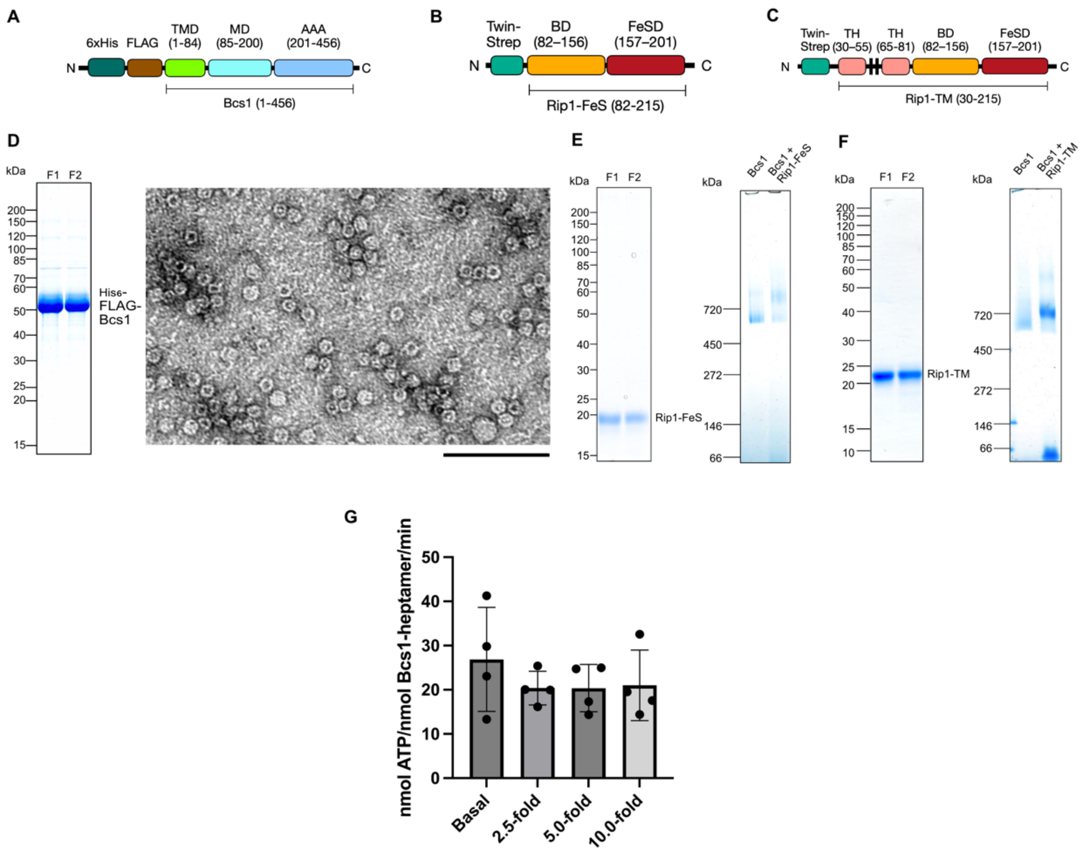

Appendix Figure S1: Reconstitution of Bcs1-Rip1 complexes from purified components.

A-C. Schematic diagrams of the used constructs (A, Bcs1; B, Rip1-FeS; C, Rip1-TM) with protein domains labelled and domain boundaries indicated in brackets. TMD = Bcs1 transmembrane domain; MD = Bcs1 middle domain; AAA = Bcs1 triple-A domain; BD = Rip1 base domain; FeSD = Rip1 2Fe-2S cluster binding subdomain; TH = Rip1 transmembrane helix. D-F. Purification of Bcs1 (D), Rip1-FeS (E) and Rip1-TM (F) from *S. cerevisiae.* D, left panel: SDS-PAGE showing two fractions of purified Bcs1 after size exclusion chromatography. 15 µl were loaded on the gel. Right panel: representative uranyl acetate-stained negative stain-TEM image of purified Bcs1. The length of the scale bar is 0.1 μm. E, SDS-PAGE showing two fractions of purified Rip1-FeS (15 µl loaded) after size exclusion chromatography (left panel) and Blue Native (BN) showing the *in vitro* reconstitution of the Bcs1-Rip1-FeS complex. F, SDS-PAGE showing two fractions of purified Rip1-FeS (15 µl loaded) after size exclusion chromatography (left panel) and Blue Native (BN) showing the *in vitro* reconstitution of the Bcs1-Rip1-TM complex. G, ATPase activity of Bcs1 in absence and presence of 2.5, 5.0 and 10.0-fold excess Rip1-TM substrate, as measured by an NADH-coupled fluorescence decay ATPase assay. Bars show mean rates of ATP hydrolysis ± 1 Standard deviation, with dots indicating replicate (n=3) independent assays.

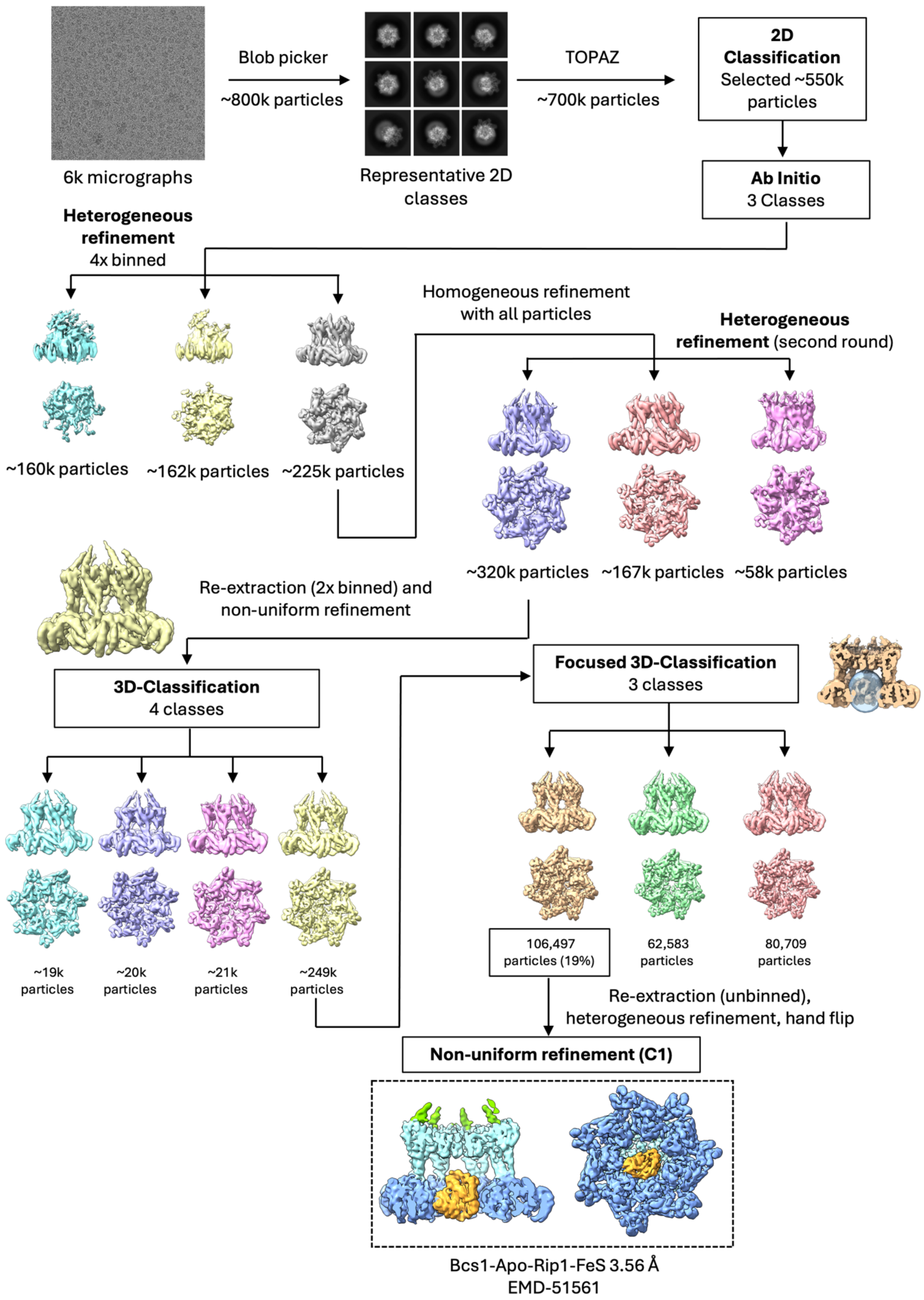

Appendix Figure S2: Cryo-EM data analysis of the Apo Bcs1-Rip1-FeS complex.

From a total of 6,044 micrographs, 546,208 particles were selected after particle picking using Topaz (ref) and 2D classification in CryoSPARC. Ab initio reconstruction followed by two

rounds of heterogeneous refinement yielded a particle subset with (320,760 particles) with substrate density. This reconstruction was further sub-sorted by 3D classification with a mask focusing on matrix vestibule of Bcs1, resulting in a final class (106,497 particles) with enriched density for Rip1-FeS. This class was refined to a final resolution of 3.56 Å.

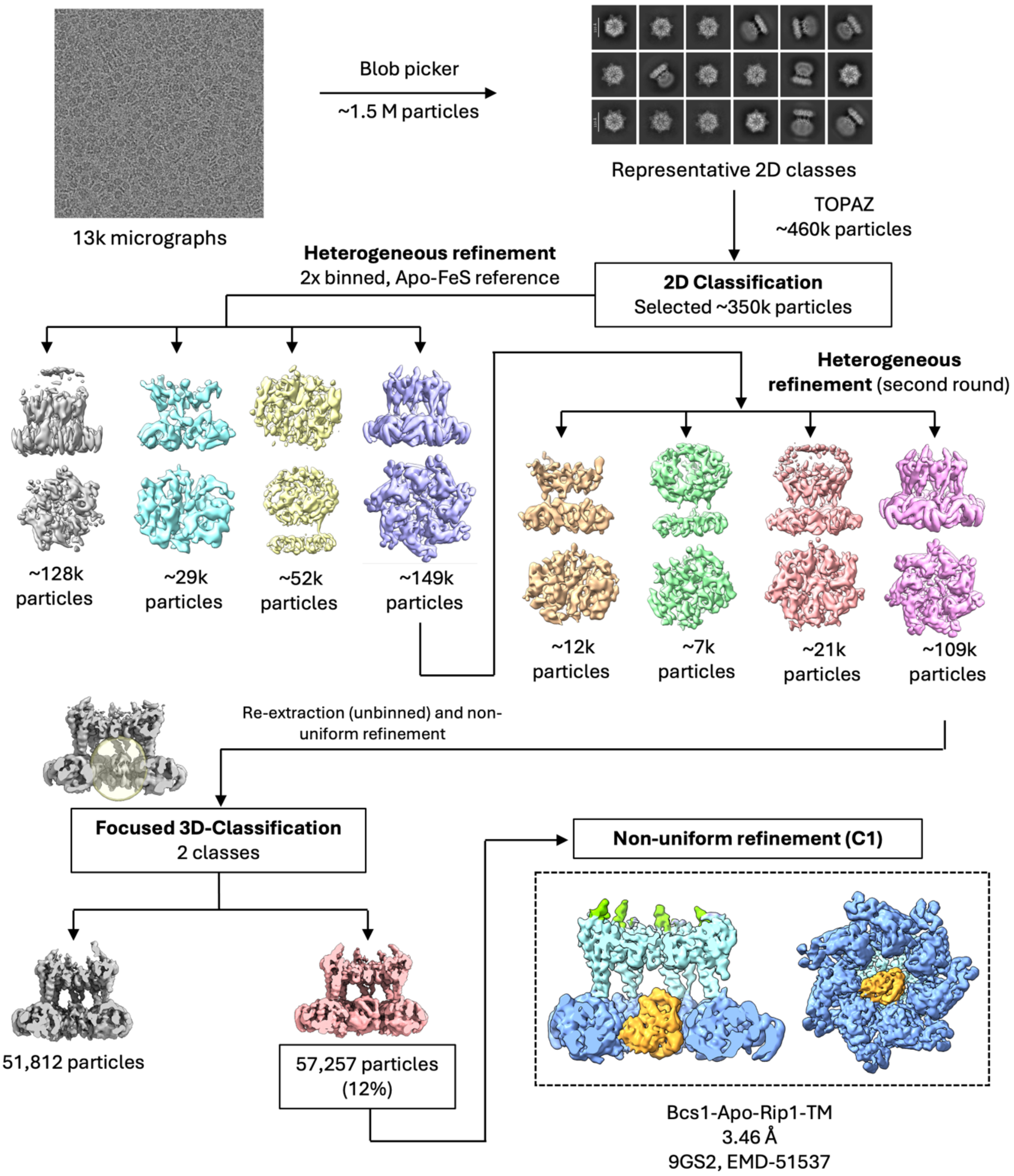

Appendix Figure S3: Cryo-EM data analysis of the Apo Bcs1-Rip1-TM complex.

This dataset was processed with a similar strategy to Bcs1-Rip1-FeS. From a total of 13,310 micrographs, 460,919 particles were selected. Heterogeneous refinement followed by (focused) 3D classification resulted in a final class (57,257 particles) with enriched density for Rip1-TM that was refined to a final resolution of 3.46 Å.

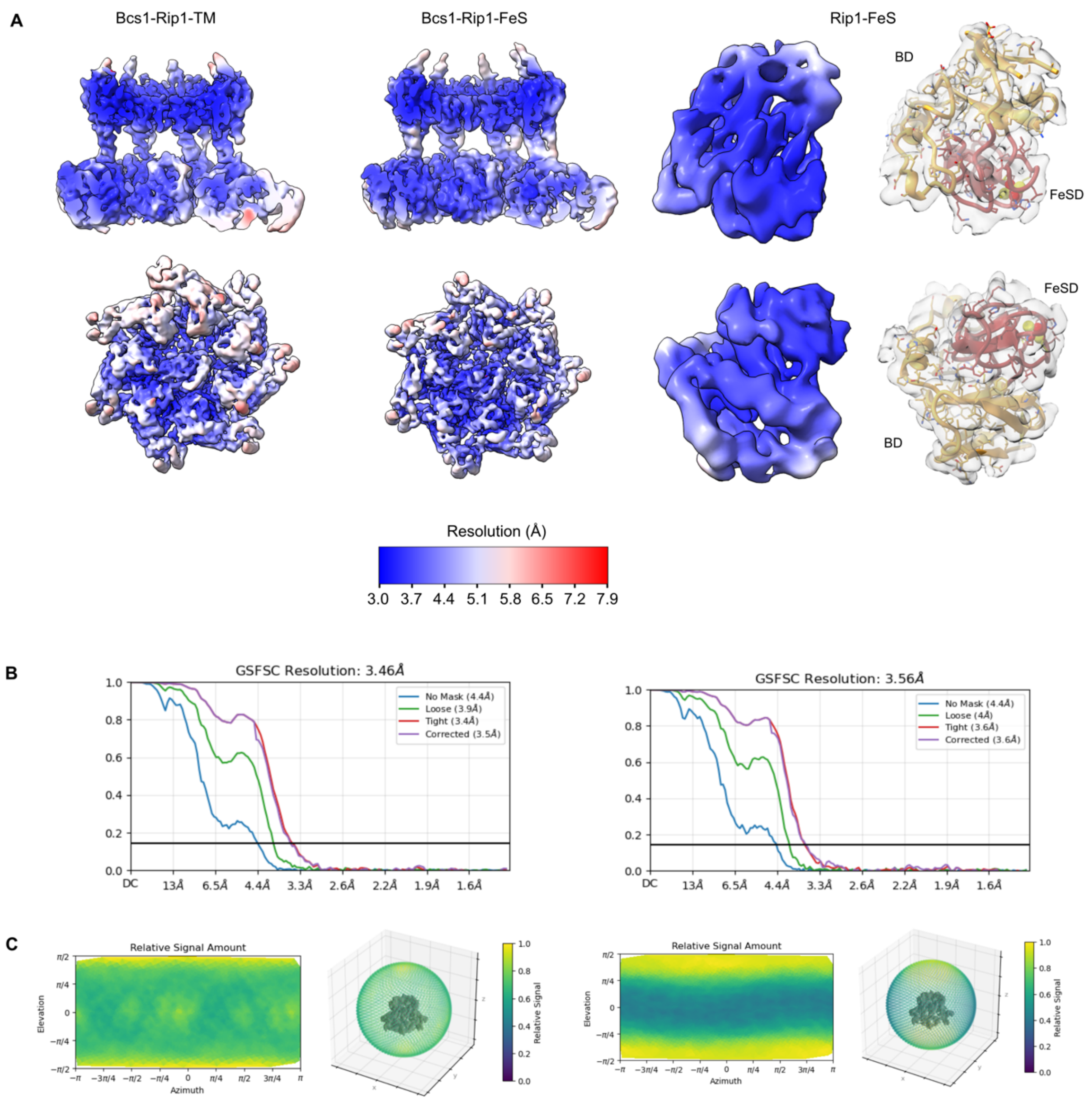

Appendix Figure S4: Local and overall resolution of Bcs1-Rip1-FeS and Bcs1-Rip1-TM maps

A, Cryo-EM maps of Bcs1-Rip1-TM (left), Bcs1-Rip1-FeS (middle) and isolated density for Rip1 (from the Bcs1-Rip1-TM reconstruction) low-pass filtered and colored according to local resolution as determined with CryoSPARC and fitted into density, subdivided into base domain (BD) and 2Fe-2S cluster binding subdomain (FeSD). Shown are cut side views (top) and bottom views (bottom) facing the matrix vestibule. B, Gold-standard Fourier Shell Correlation (GSFSC) resolution curves from CryoSPARC (right: Bcs1-Rip1-TM; left Bcs1-Rip1-FeS) displaying the average resolution using various automatically applied masks. C, angular distribution plots for final reconstructions obtained from CryoSPARC (right: Bcs1-Rip1-TM; left Bcs1-Rip1-FeS).

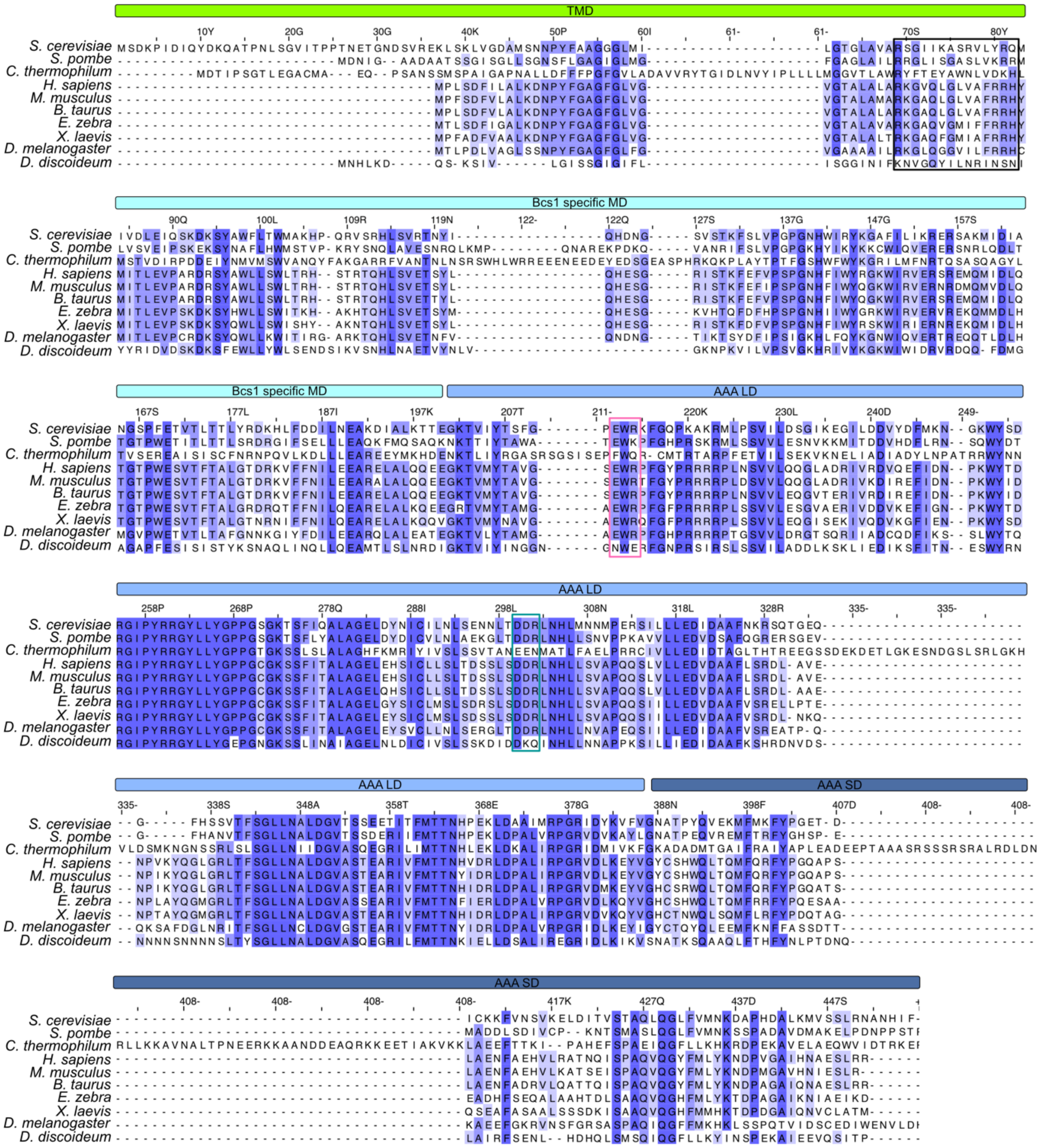

Appendix Figure S5: Multiple sequence alignment of Bcs1

Multiple sequence alignment of Bcs1 from selected species generated by Clustal Omega (Madeira *et al*, 2024) and displayed using Jalview (Waterhouse *et al*, 2009), highlighting major structural elements and colouring aminoacids according to their percentage of identity. TMD (aa1-84): transmembrane domain, MD (aa85-202): middle domain, AAA LD (aa203-387): AAA ATPase large domain, AAA SD (aa388-456): AAA ATPase small domain. Black box encloses positively charged region in the TMD domain, pink box encloses the EWR domain and green box encloses the DDR domain. Last 174 aminoacids of the *C. thermophilum* sequence have been omitted for clarity.

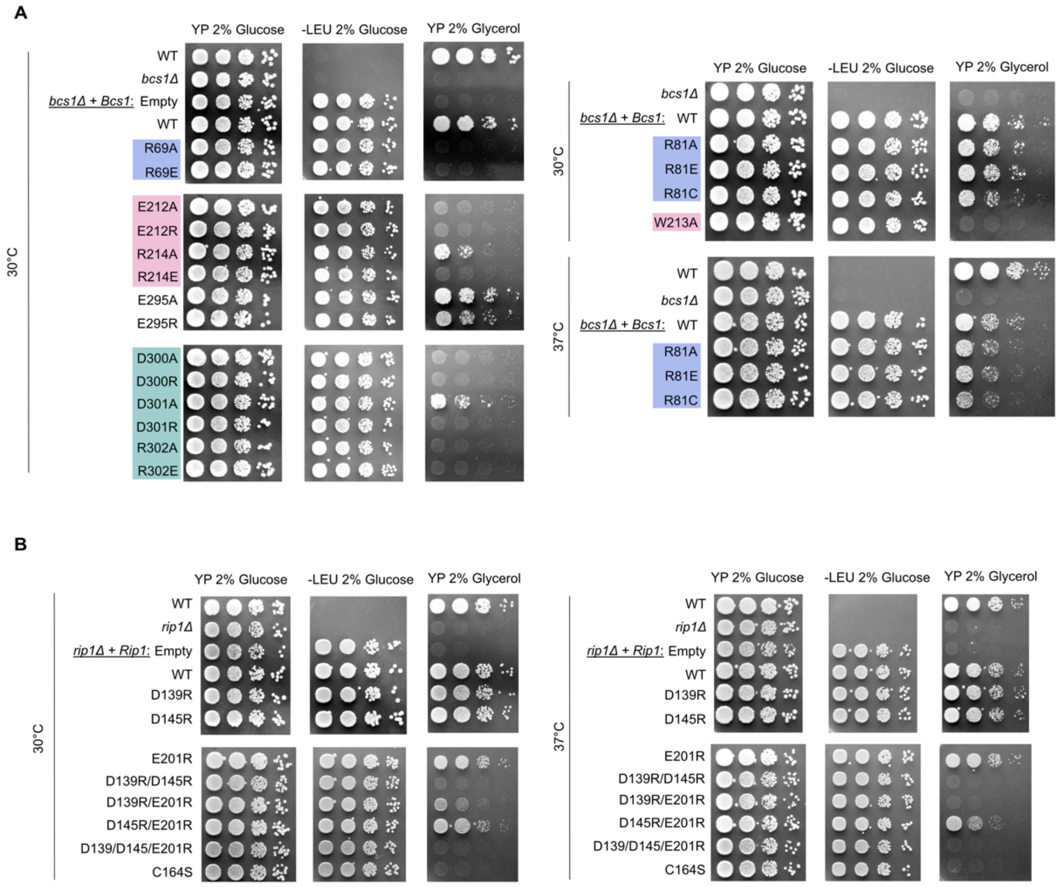

Appendix Figure S6: Mutational analysis of Bcs1 and Rip1

A, Growth assays (10-fold serial dilutions) of yeast cells (*bcs1Δ*) on a fermentable (glucose) or non-fermentable (glycerol) carbon source. Right panels show growth controls on rich media (YP) and middle panels show a control for presence of the Bcs1-expressing plasmid. The left panels show growth on YP supplemented with 2% glycerol. Growth was monitored at 30°C or 37°C. The residues from the EWR and the DDR motives, as well as those from the positive patch in the TD are highlighted with a pink, a green and a blue box, respectively. B, Growth assays for Rip1 were carried out in a similar way on a *rip1Δ* background and from a Rip1-expressing plasmid (see Methods section for details). Excerpts of this figure are shown in Figure 2, panels D-E, and Figure 4 panel E, to emphasize the residues displayed in Figure 2A-C and Figure 4D, respectively.

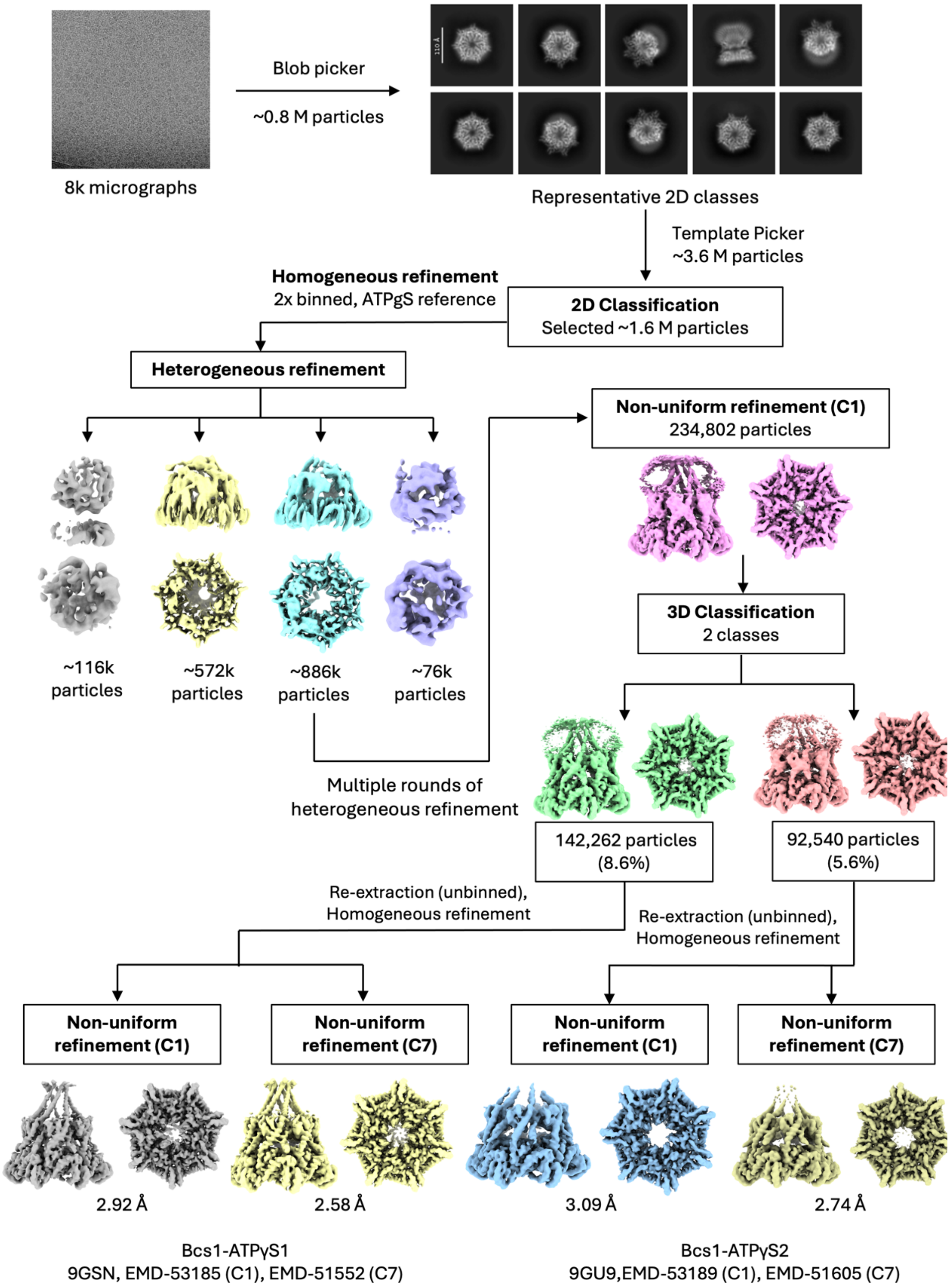

Appendix Figure S7: Cryo-EM data analysis of the ATPγS states of Bcs1

From a total of 8825 micrographs, 234,802 particles were selected after multiple rounds of heterogeneous refinement in CryoSPARC. 3D classification allowed for the separation into two classes that represent the states 1 (142,262) and 2 (92,540), further refined to yield C7-symmetric reconstructions with final resolution of 2.58 Å and 2.74 Å, respectively.

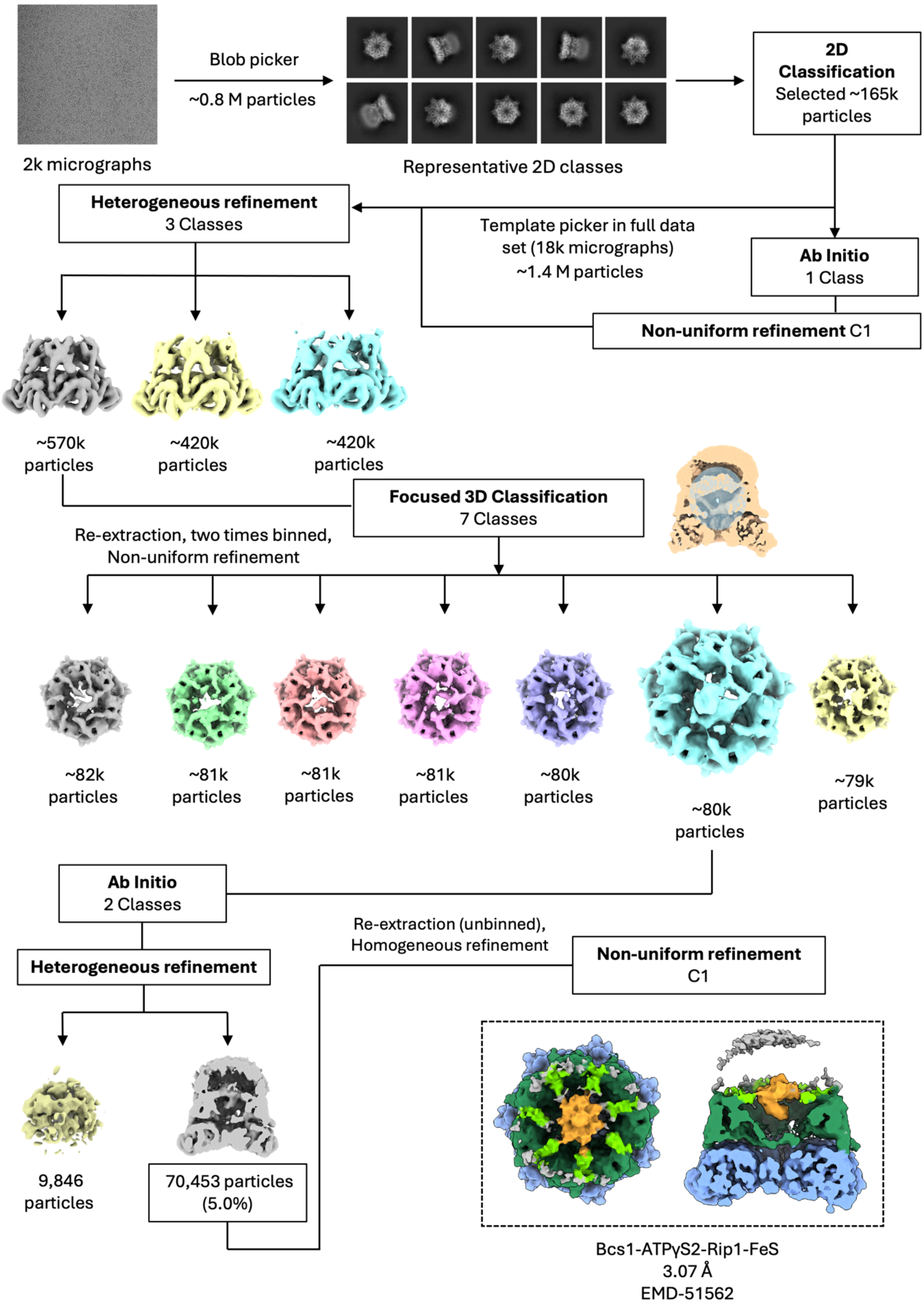

Appendix Figure S8: Cryo-EM data analysis of the ATPγS2-Rip1-FeS state of Bcs1

From a total data set of 18,385 micrographs, 567,113 particles were selected after heterogeneous refinement in CryoSPARC. Focused classification yielded a final subset of 70,453 which was refined to a resolution of 3.07 Å.

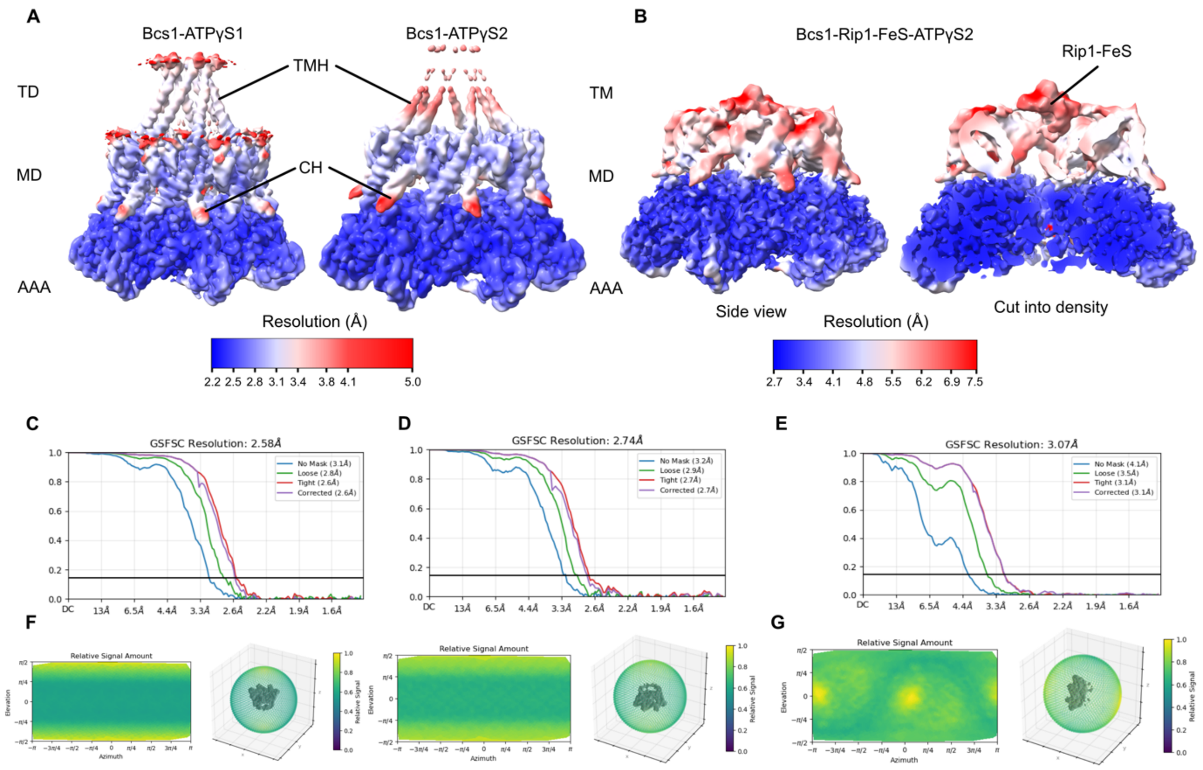

Appendix Figure S9: Local and overall resolution of Bcs1-ATPγS and Bcs1- Rip1-FeS-ATPγS maps.

A, B. Cryo-EM maps of Bcs1-ATPγS1 and Bcs1-ATPγS2 (A) and of Bcs1-Rip1-FeS-ATPγS2 (B) low-pass filtered and colored according to local resolution as determined with CryoSPARC. Maps and shown as side views (A and B left panel) or cut side view (B, right panel). Note that for Bcs1-ATPγS2 the TM basket helices show lower local resolution as in Bcs1-ATPγS1and become more flexible. C-E, Gold-standard Fourier Shell Correlation (GSFSC) resolution curves from CryoSPARC (C: Bcs1-ATPγS1; D: Bcs1-ATPγS2; E: Bcs1-Rip1-FeS-ATPγS2) displaying the average resolution using various automatically applied masks. F, G. Angular distribution plots for final reconstructions obtained from CryoSPARC (F, right: Bcs1-ATPγS1; F, middle: Bcs1-ATPγS2) and Bcs1-Rip1-FeS-ATPγS2 (G).

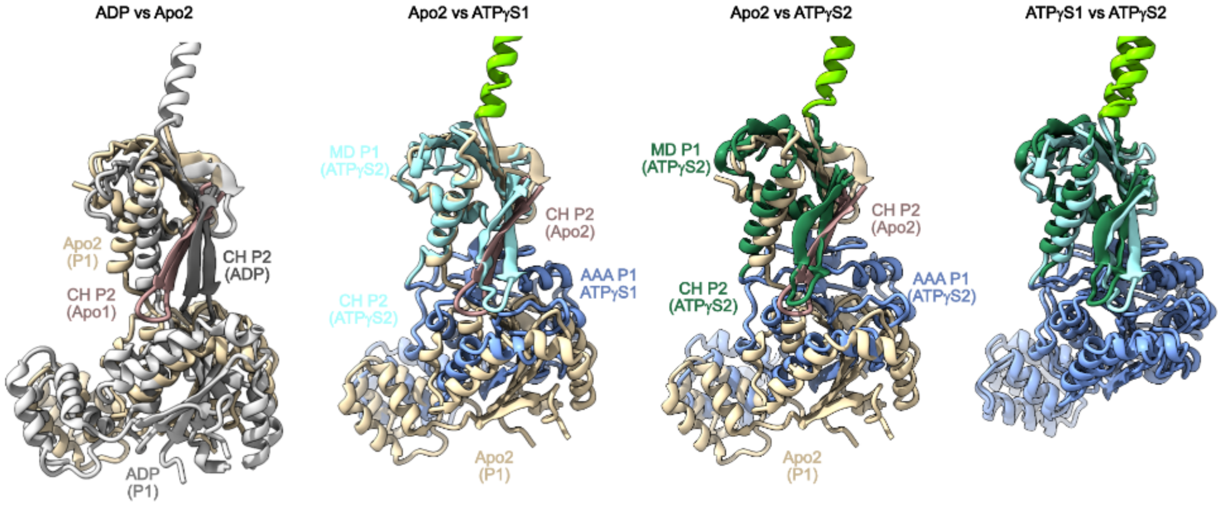

Appendix Figure S10: Conformation of the middle domain

Alignment on middle domain of one protomer (P2); displayed is the neighbouring protomer (P1) without the CH as well as the CH of the aligned protomer (P2). We see a tilt of the middle domain and a flip-out of the CH in ADP vs Apo2 (see (Kater *et al.*, 2020), Fig. 5d). We see that ATPγS1 is very similar to Apo2 with respect of the MD, only the CH is in different conformation. We see that the MD of ATPγS2 is slightly shifted compared to Apo2 and compared ATPγS2.

	Bcs1-Apo-Rip1-FeS	Bcs1-Apo-Rip1-TM	Bcs1-ATPγS1	Bcs1-ATPγS2	Bcs1-ATPγS2-Rip1-FeS
	(EMDB-51561)	(EMDB-51537)	(C1: EMDB-53185; C7: EMDB-51552)	(C1: EMDB-53189; C7: EMDB-51605)	(EMDB-51562)
		(PDB 9GS2)	(PDB 9GSN)	(PDB 9GU9)	
**Data collection and processing**					
**Magnification**	165,000	165,000	165,000	165,000	165,000
**Voltage (kV)**	300	300	300	300	300
**Electron exposure (e–/Å^2)**	33	40	40	40	40
**Defocus range (µm)**	0.5-3.0	0.5-3.0	0.5-3.0	0.5-3.0	0.5-3.0
**Pixel size (Å)**	0.727	0.727	0.727	0.727	0.727
**Symmetry imposed**	C1	C1	C7	C7	C1
**Initial particle images (no.)**	309,698	460,919	234,802	234,802	567,113
**Final particle images (no.)**	106,497	57,257	142,262	92,540	70,453
**Map resolution (Å)**	3.56	3.46	2.58	2.74	3.07
**FSC threshold (0.143)**					
**Map resolution range (Å)**	3.0-7.9	3.0-7.9	2.2-5.0	2.2-5.0	2.7-7.5
**Refinement**					
**Initial model used (PDB code)**		Alphafold, PDB-1KB9	de novo	de novo	
**Model resolution (Å)**		4.2	2.9	3.1	

**FSC threshold (0.5)**					
**Map sharpening *B* factor (Å^2)**	-112.0	-100.8	-113.9	-123.9	-99.6
**Model composition**					
**Non-hydrogen atoms**		44,657	43,140	43,162	
**Protein residues**		2798	2695	2695	
**Ligands**		1 FES	7 AGS	7 AGS	
***B* factors (Å^2)**					
**Protein**		196.10	132.91	155.15	
**Ligand**		230.23	115.45	119.27	
**R.m.s. deviations**					
**Bond lengths (Å)**		0.004	0.004	0.004	
**Bond angles (°)**		0.737	0.756	0.770	
**Validation**					
**MolProbity score**		1.65	1.60	1.55	
**Clashscore**		5.82	5.93	4.87	
**Poor rotamers (%)**		0.08	0.77	0.90	
**Ramachandran plot**					
**Favored (%)**		95.18	96.04	95.78	
**Allowed (%)**		4.71	3.92	4.22	
**Disallowed (%)**		0.11	0.04	0.00	

Appendix Table S1. CryoEM data collection, model refinement and validation statistics

Species	State	PDB	EMDB	TM-helices	Symmetry	Reference
S. cerevisiae	Bcs1-ADP	6SH3	10192	Resolved	C7	(Kater *et al.*, 2020)
S. cerevisiae	Bcs1-Apo1	6SH4	10193	Resolved	C7	(Kater *et al.*, 2020
S. cerevisiae	Bcs1-Apo2	6SH5	10194	Partially resolved	C7	(Kater *et al.*, 2020
S. cerevisiae	Bcs1-Apo1-Rip1-FeS	-	51561	Partially resolved	C1	This study
S. cerevisiae	Bcs1-Apo1-Rip1-TM	9GS2	51537	Partially resolved	C1	This study
S. cerevisiae	Bcs1-ATPγS1	-	53185	Resolved	C1	This study
S. cerevisiae	Bcs1-ATPγS1	9GSN	51552	Resolved	C7	This study
S. cerevisiae	Bcs1-ATPγS2	-	53189	Partially resolved	C1	This study
S. cerevisiae	Bcs1-ATPγS2	9GU9	51605	Partially resolved	C7	This study
S. cerevisiae	Bcs1-ATPγS2-Rip1-FeS	-	51562	Not resolved	C1	This study
M. musculus	Bcs1-Apo	6UKP	20808	Not resolved	C7	(Tang *et al.*, 2020)
M. musculus	Bcs1-ATPγS	6UKS	20811	Not resolved	C7	(Tang *et al.*, 2020)
M. musculus	Bcs1-ATP1	8TI0	41276	Not resolved	C1	(Zhan *et al.*, 2024)
M. musculus	Bcs1-ATP1	8T5U	41061	Not resolved	C7	(Zhan *et al.*, 2024)
M. musculus	Bcs1-ATP2	8TPL	41476	Partially resolved	C1	(Zhan *et al.*, 2024)
M. musculus	Bcs1-ATP2	8TP1	41462	Resolved	C7	(Zhan *et al.*, 2024)
M. musculus	Bcs1-ADP	8T7U	41095	Resolved	C1	(Zhan *et al.*, 2024)
M. musculus	Bcs1-ADP	8T14	40954	Resolved	C7	(Zhan *et al.*, 2024)
M. musculus	Bcs1-Rip1	-	41609	Not resolved	C1	(Zhan *et al.*, 2024)
M. musculus	Bcs1-Apo	8TBY	41148	Partially resolved	C1	(Zhan *et al.*, 2024)

Appendix Table 2. Summary of Bcs1 states obtained by CryoEM.

TM helices in the TD are classified as "resolved", "partially resolved" or "not resolved" based on the deposited CryoEM maps. Of note, visibility (conformational stability) of the helices is rather poorly correlated with any state of Bcs1. This points out to a high dependence on the environment (property of the lipid/detergent micelle) and quality of the reconstructions in the TD area, which in turn is mainly driven by technical parameters like signal-to-noise ratio, the number of particles, ice thickness, thoroughness of 3D classification, etc. For example, some mouse Bcs1 reconstructions show only density of the TD helices after re-processing/increasing particle amount: While the first published mBCS1-Apo state (PDB: 6UKP; EMDB: 20808) didn't show a defined TD, a later reconstruction showed a more defined TD (PDB: 8TBY; EMDB:41148). Similar observations have been made in the yeast datasets.
