# Supplementary figures and images for: Mechanistic insights into Bcs1-mediated mitochondrial membrane translocation of the folded Rieske protein
Source: EMBO J. 2025 May 23;44(13):3720–41. doi: 10.1038/s44318-025-00459-4 (PMC12219900; doi:10.1038/s44318-025-00459-4)

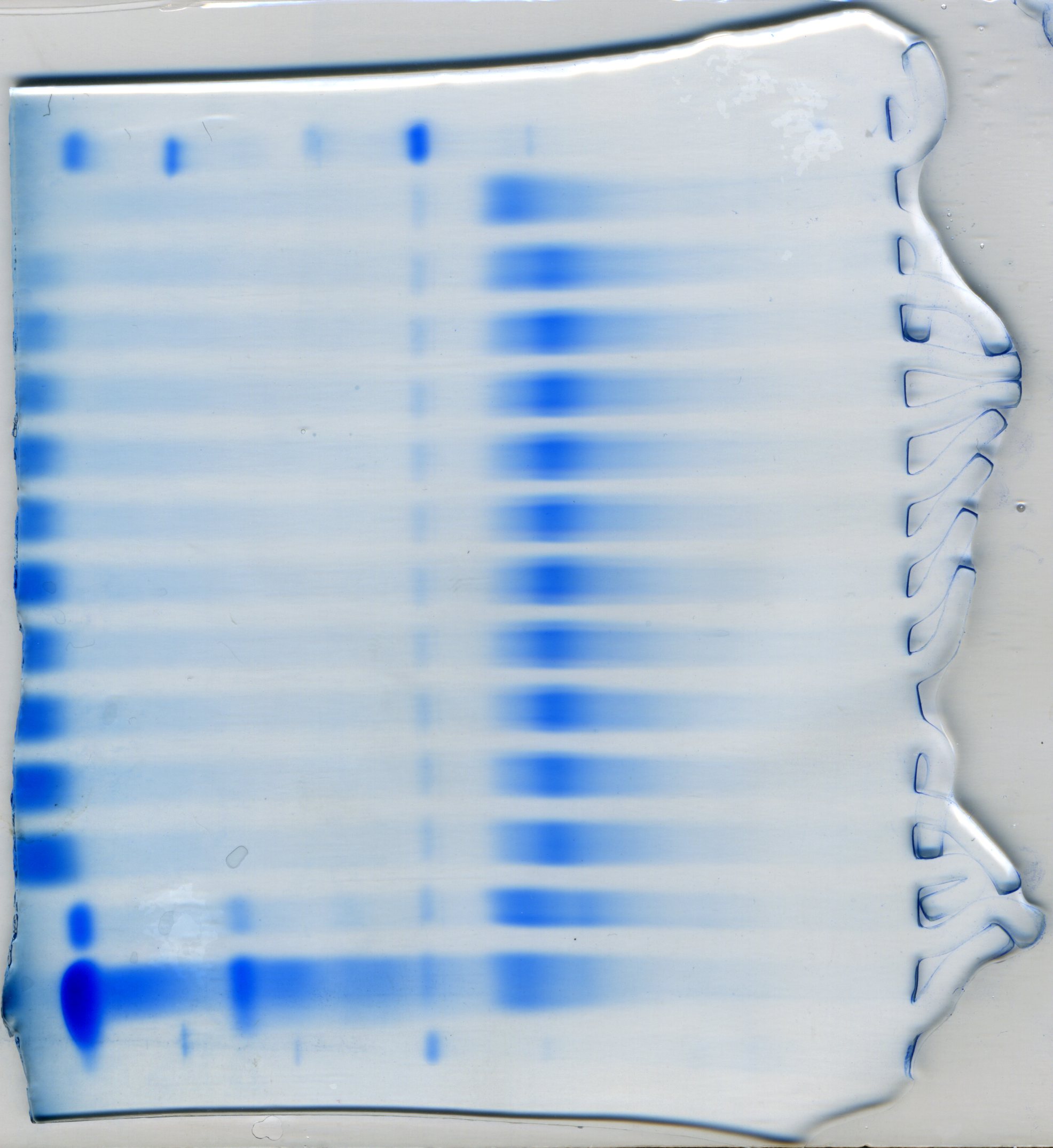

Supplement: Supplementary file 3 — Source data Fig. 1 [file 44318_2025_459_MOESM3_ESM.zip › Fig1/A/230221 BNdn82012.jpg]

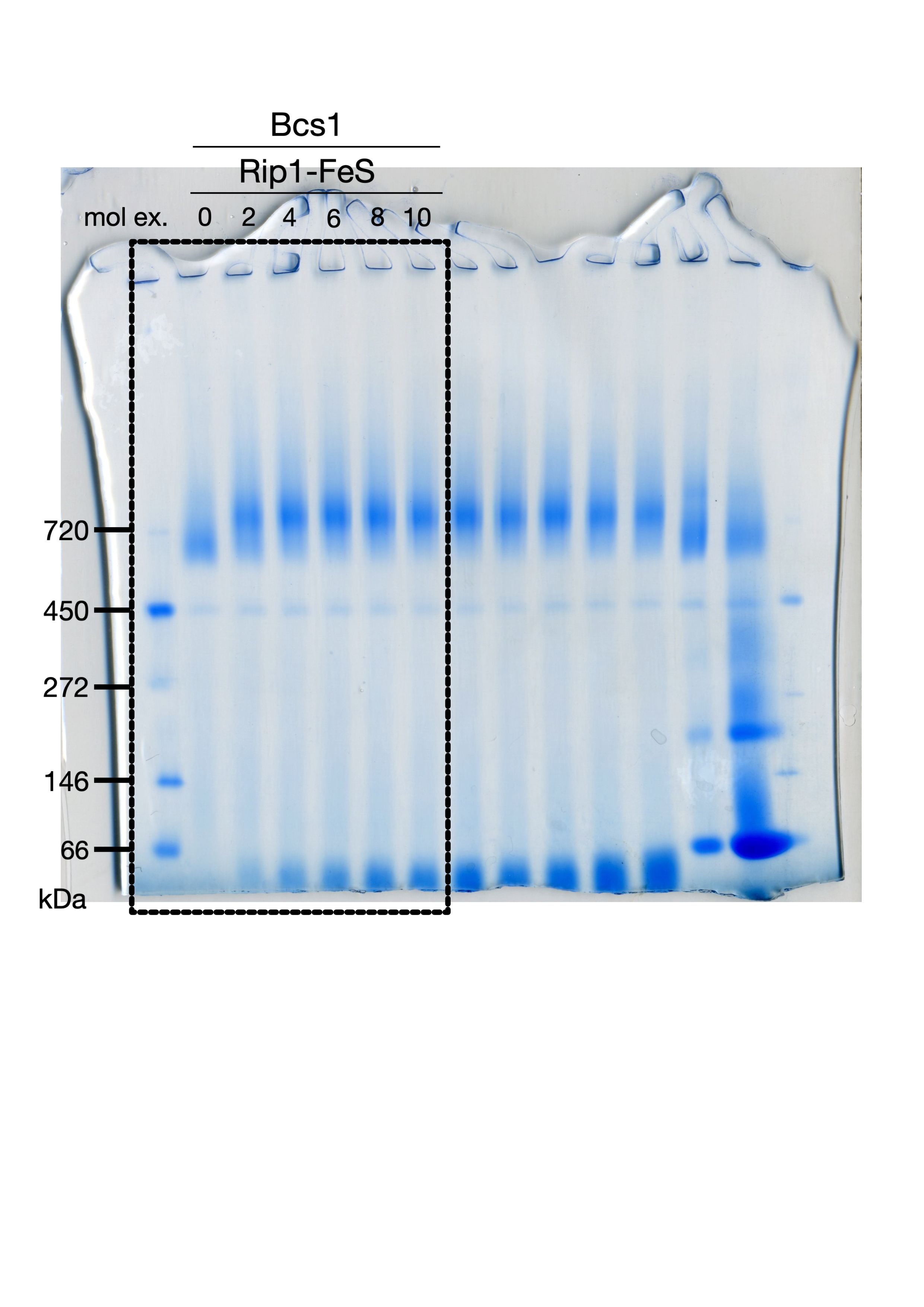

Supplement: Supplementary file 3 — Source data Fig. 1 [file 44318_2025_459_MOESM3_ESM.zip › Fig1/A/Fig1_source.png]

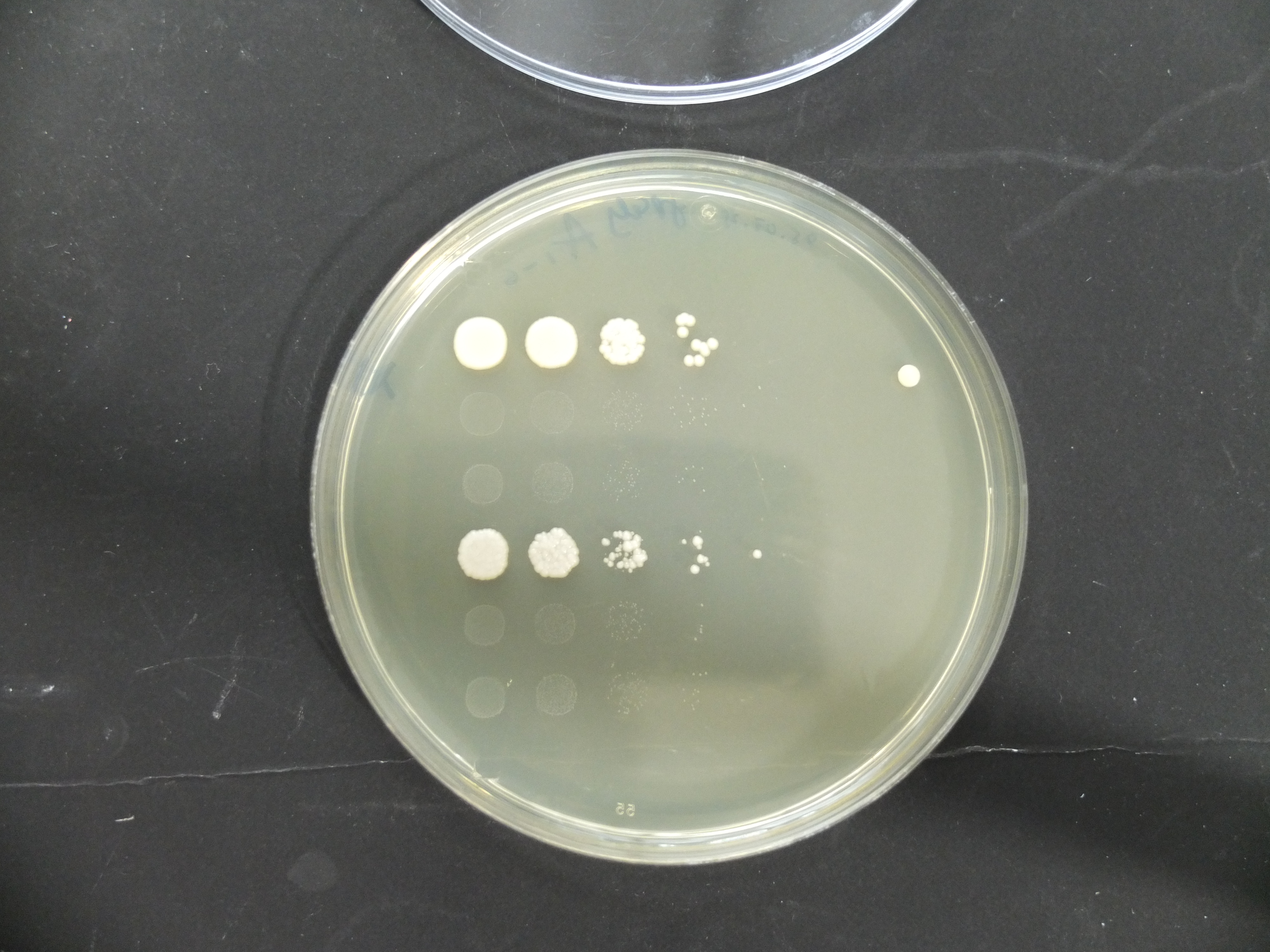

Supplement: Supplementary file 4 — Source data Fig. 2 [file 44318_2025_459_MOESM4_ESM.zip › Fig2/D/top/DSCF8245.JPG]

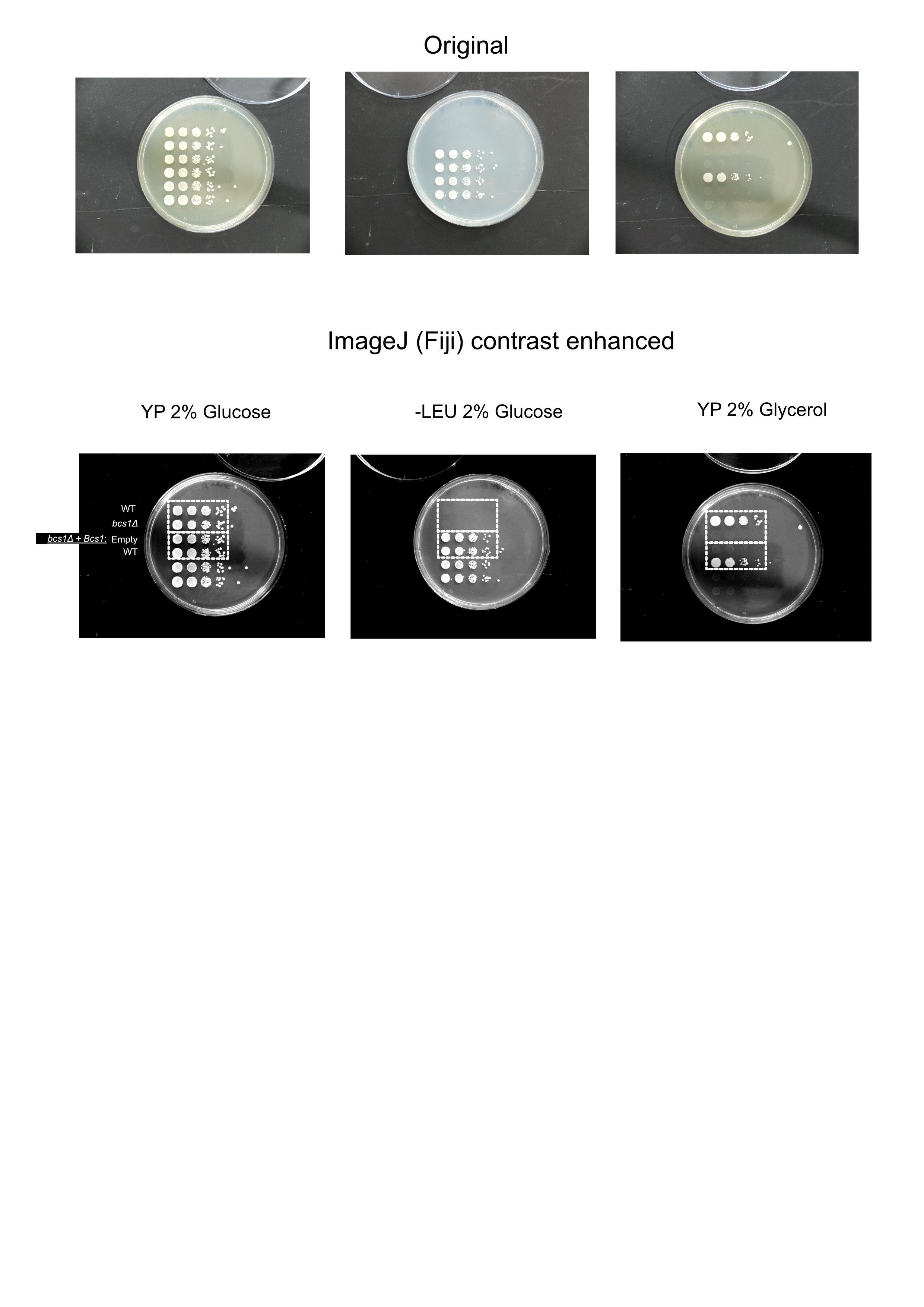

Supplement: Supplementary file 4 — Source data Fig. 2 [file 44318_2025_459_MOESM4_ESM.zip › Fig2/D/top/Fig2Dtop_source.png]

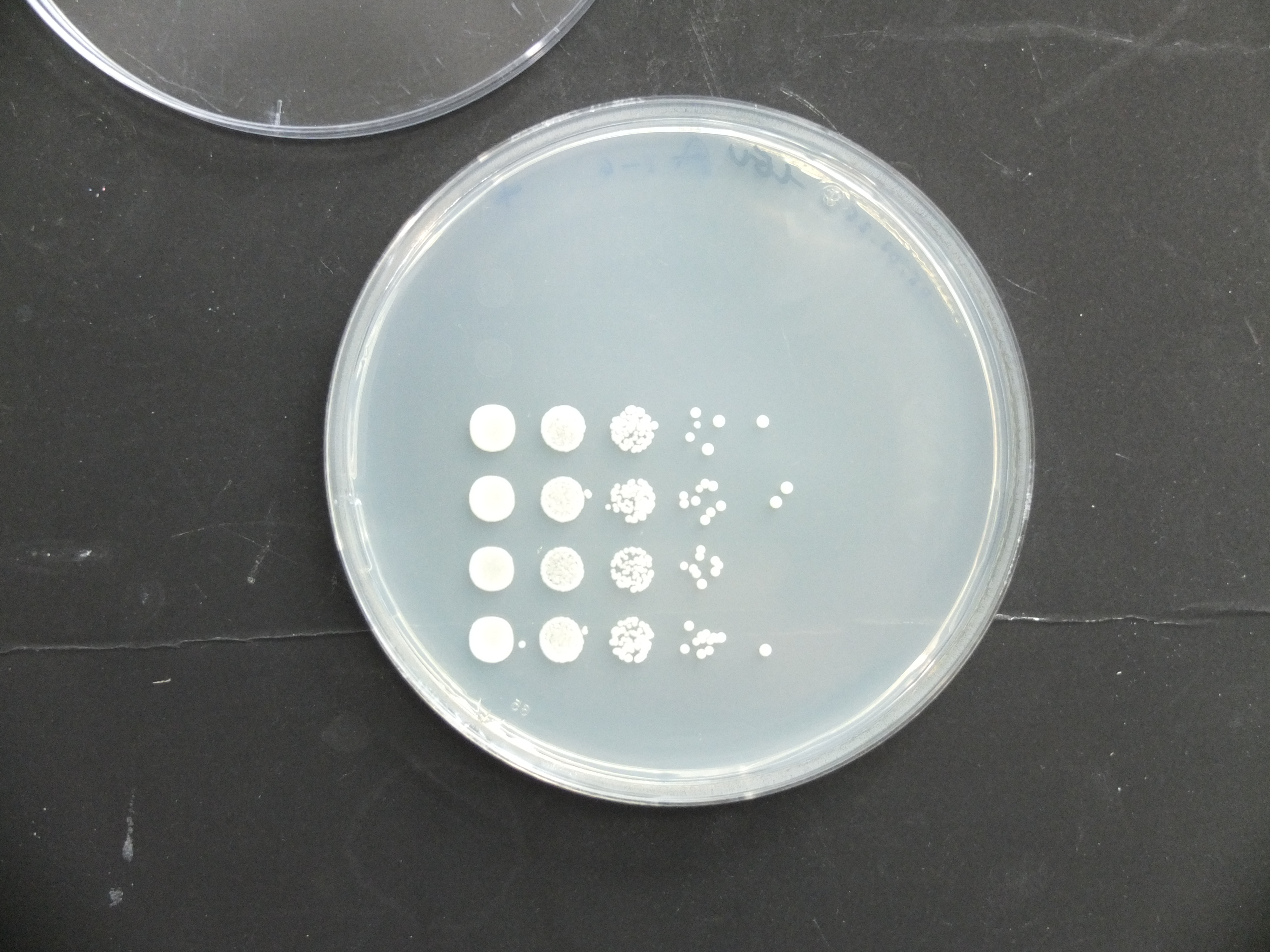

Supplement: Supplementary file 4 — Source data Fig. 2 [file 44318_2025_459_MOESM4_ESM.zip › Fig2/D/top/DSCF8227.JPG]

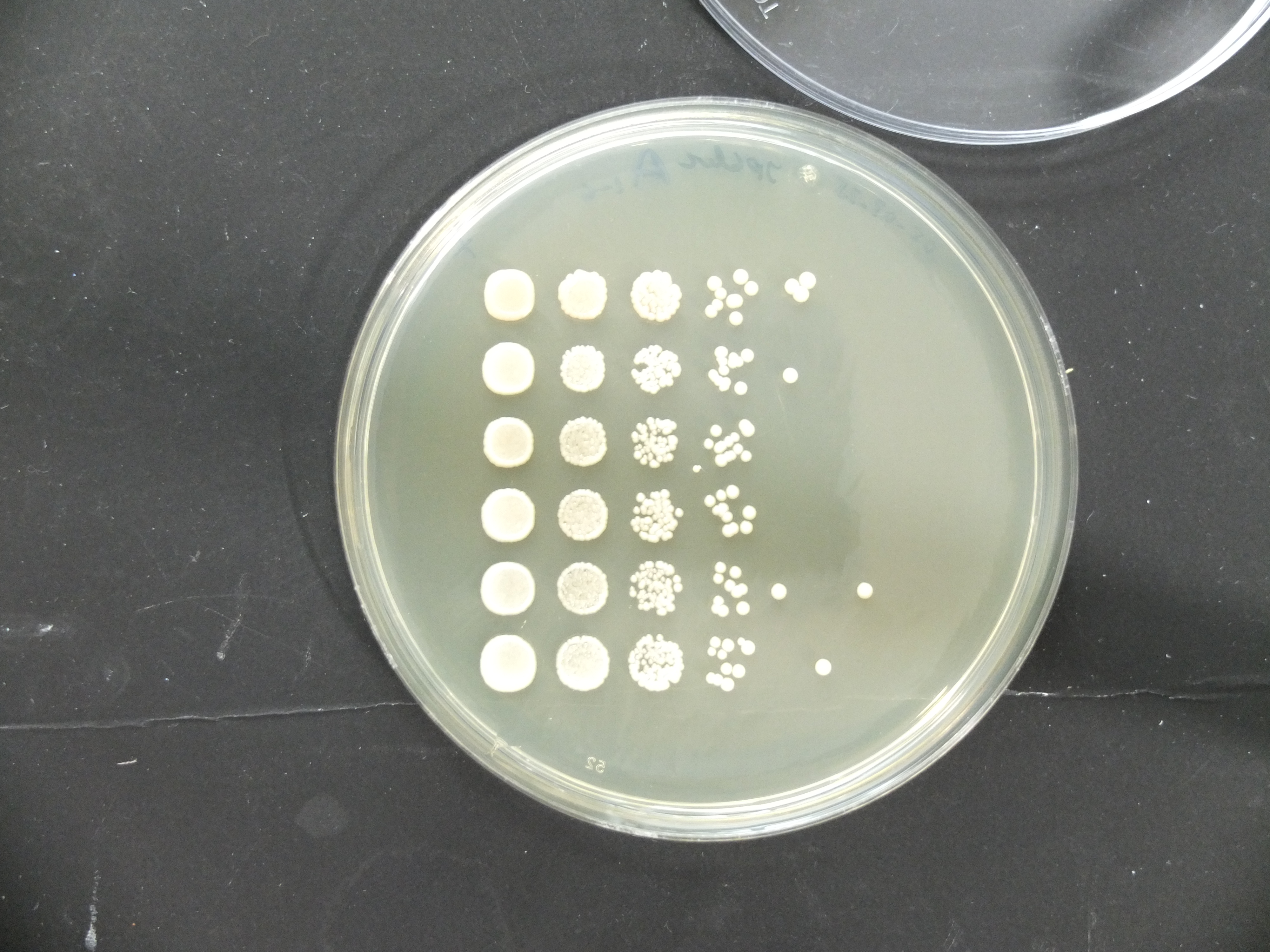

Supplement: Supplementary file 4 — Source data Fig. 2 [file 44318_2025_459_MOESM4_ESM.zip › Fig2/D/top/DSCF8205.JPG]

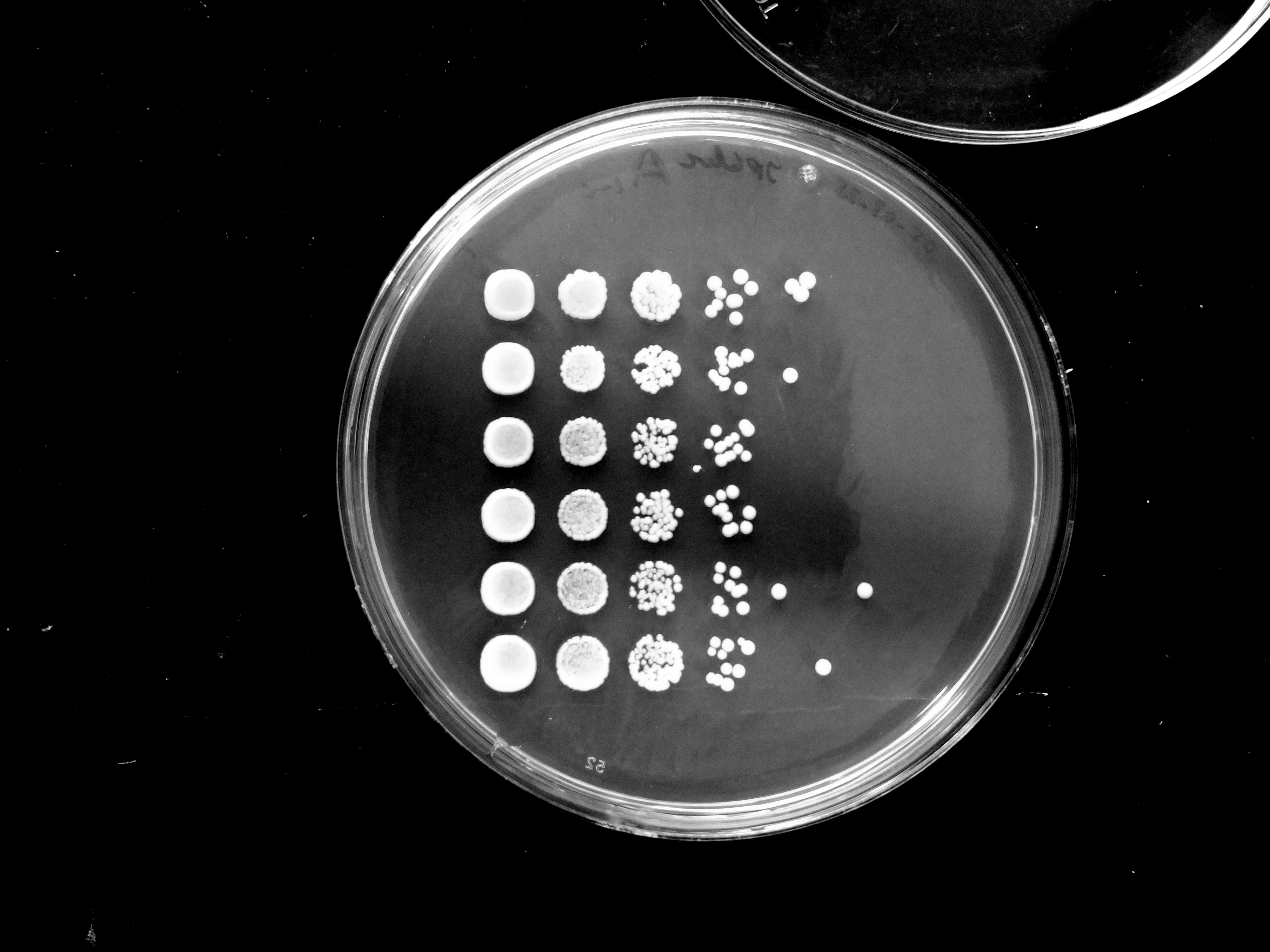

Supplement: Supplementary file 4 — Source data Fig. 2 [file 44318_2025_459_MOESM4_ESM.zip › Fig2/D/top/DSCF8205-2.jpg]

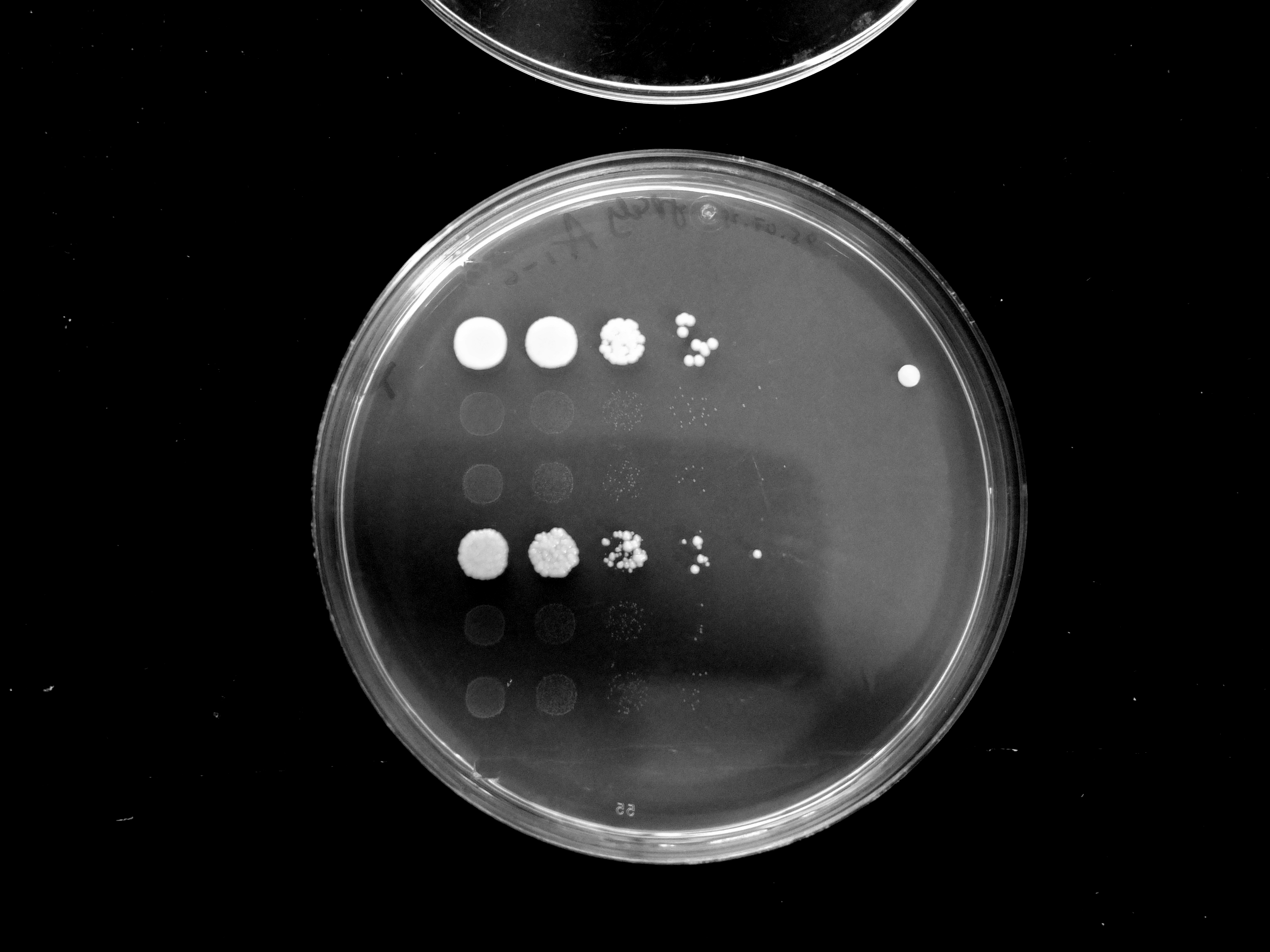

Supplement: Supplementary file 4 — Source data Fig. 2 [file 44318_2025_459_MOESM4_ESM.zip › Fig2/D/top/DSCF8245-2.tif]

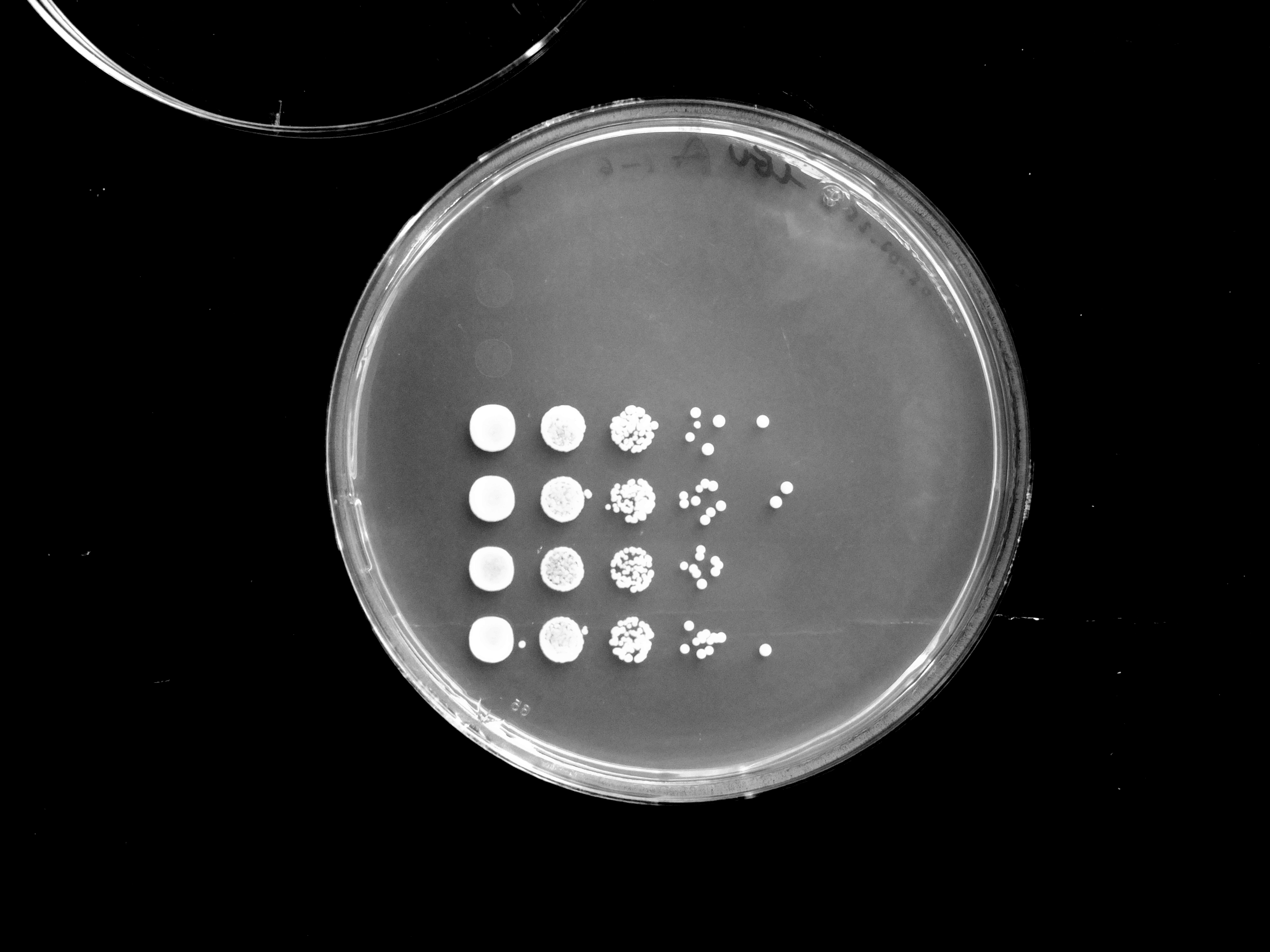

Supplement: Supplementary file 4 — Source data Fig. 2 [file 44318_2025_459_MOESM4_ESM.zip › Fig2/D/top/DSCF8227-2.tif]

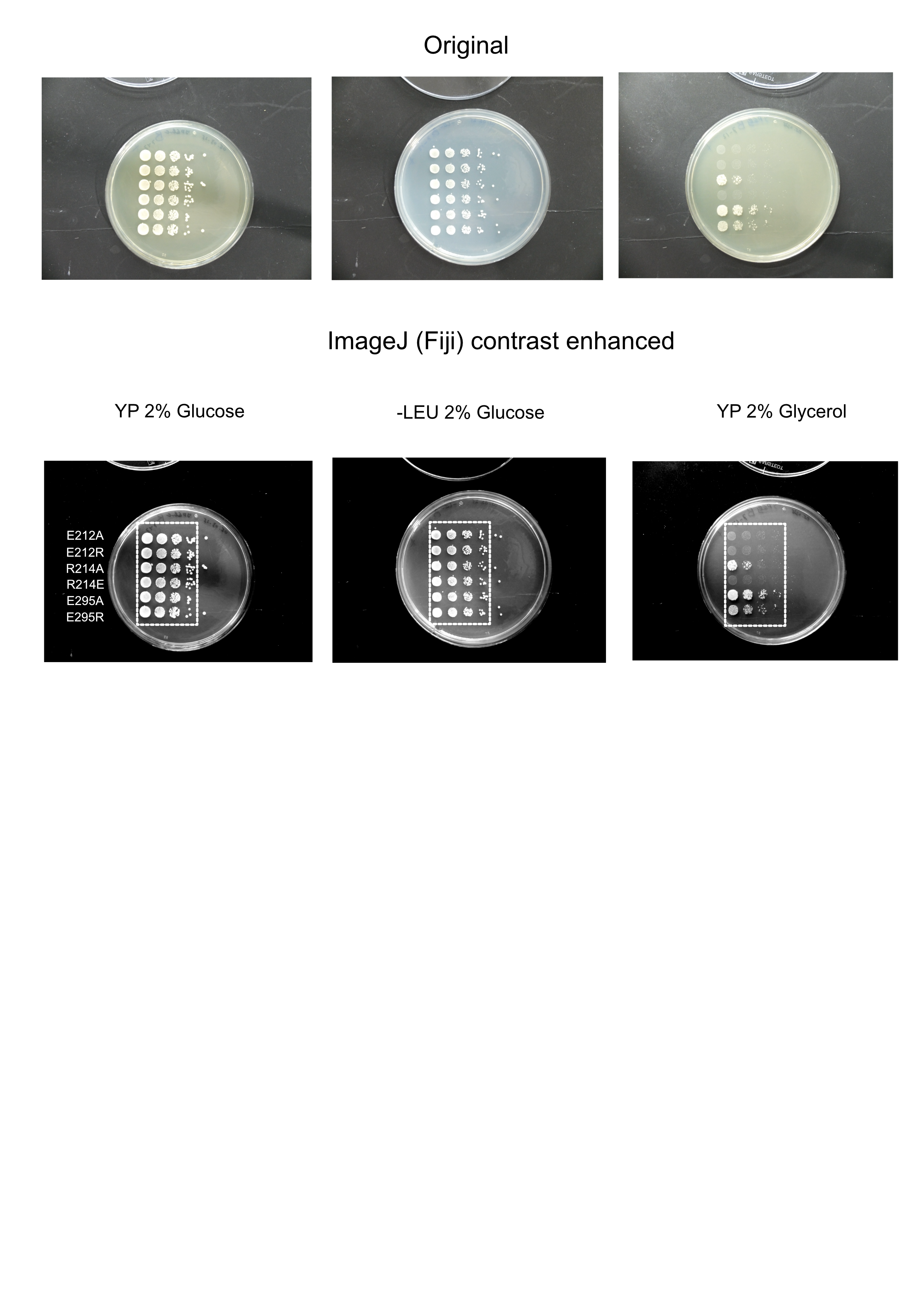

Supplement: Supplementary file 4 — Source data Fig. 2 [file 44318_2025_459_MOESM4_ESM.zip › Fig2/D/middle/Fig2Dmiddle_source.png]

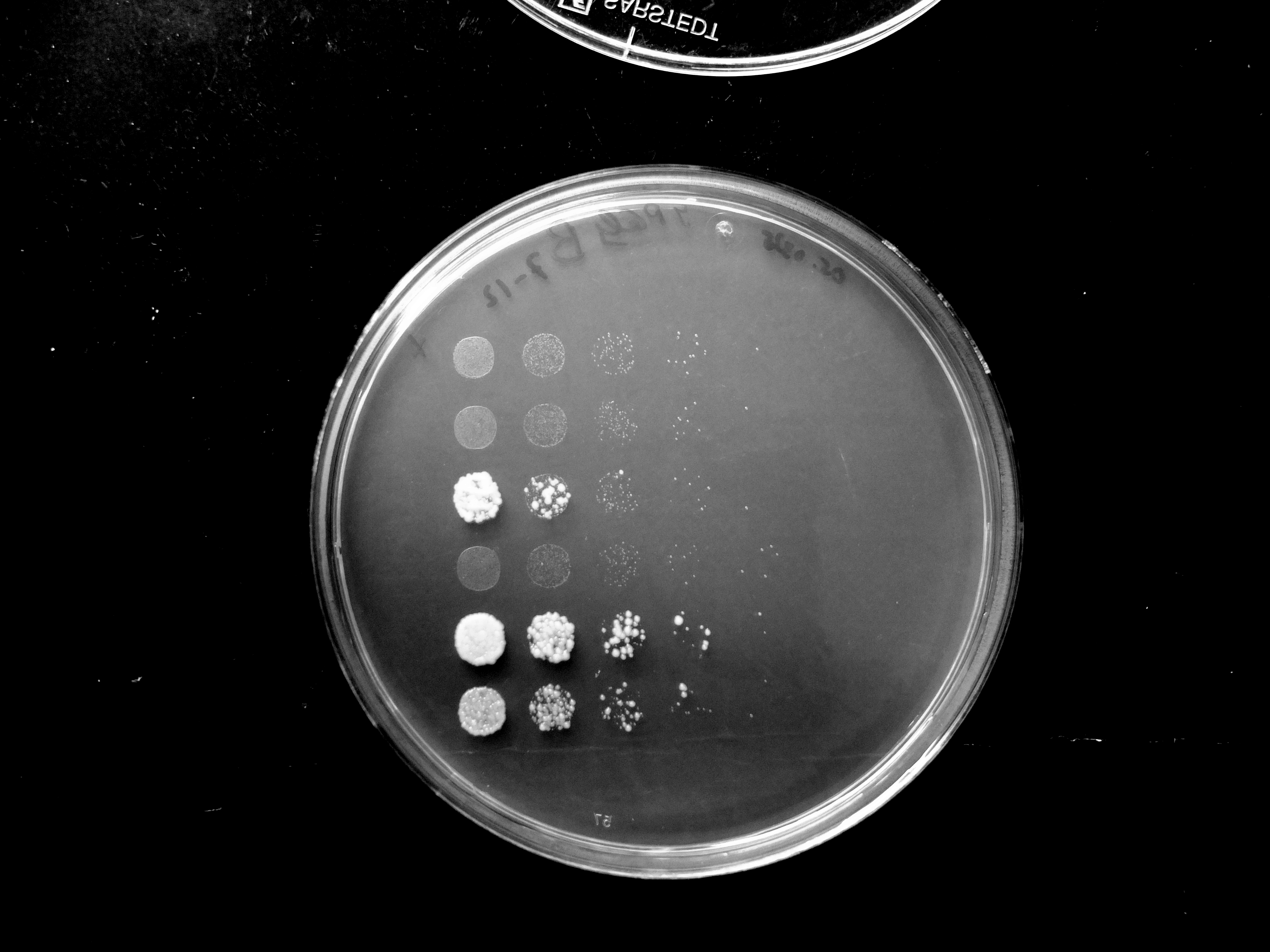

Supplement: Supplementary file 4 — Source data Fig. 2 [file 44318_2025_459_MOESM4_ESM.zip › Fig2/D/middle/DSCF8253-2.tif]

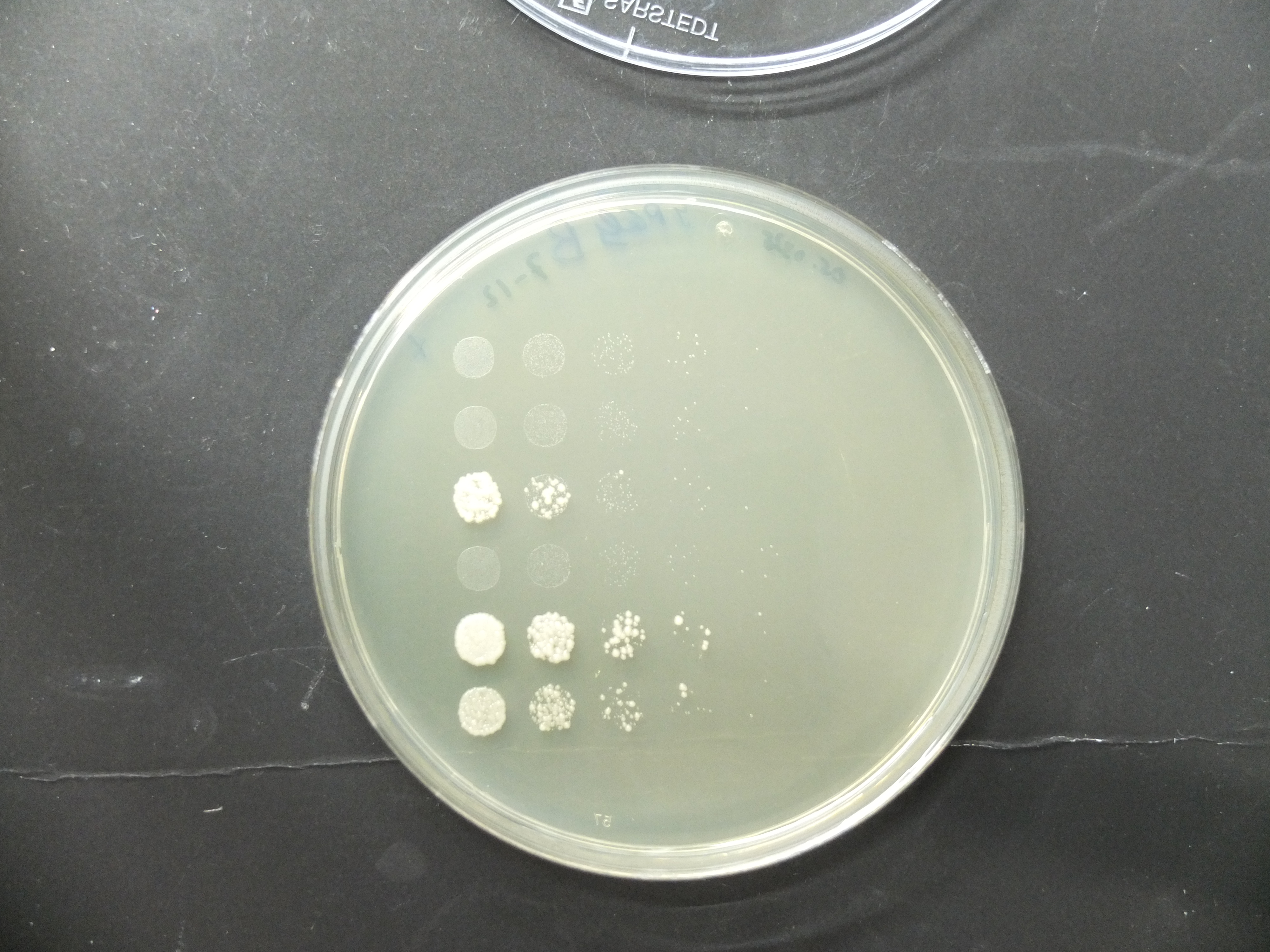

Supplement: Supplementary file 4 — Source data Fig. 2 [file 44318_2025_459_MOESM4_ESM.zip › Fig2/D/middle/DSCF8253.JPG]

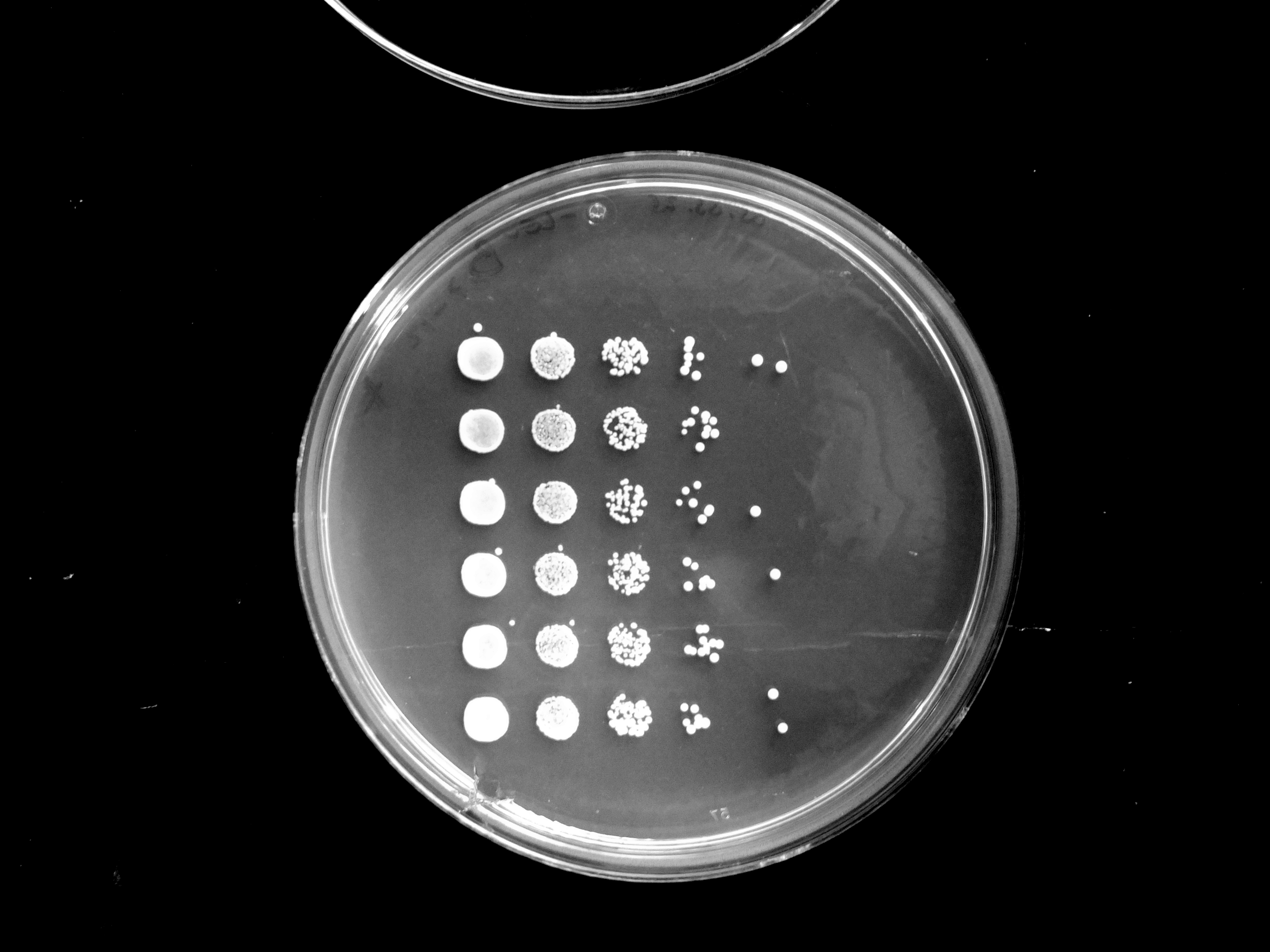

Supplement: Supplementary file 4 — Source data Fig. 2 [file 44318_2025_459_MOESM4_ESM.zip › Fig2/D/middle/DSCF8233-2.tif]

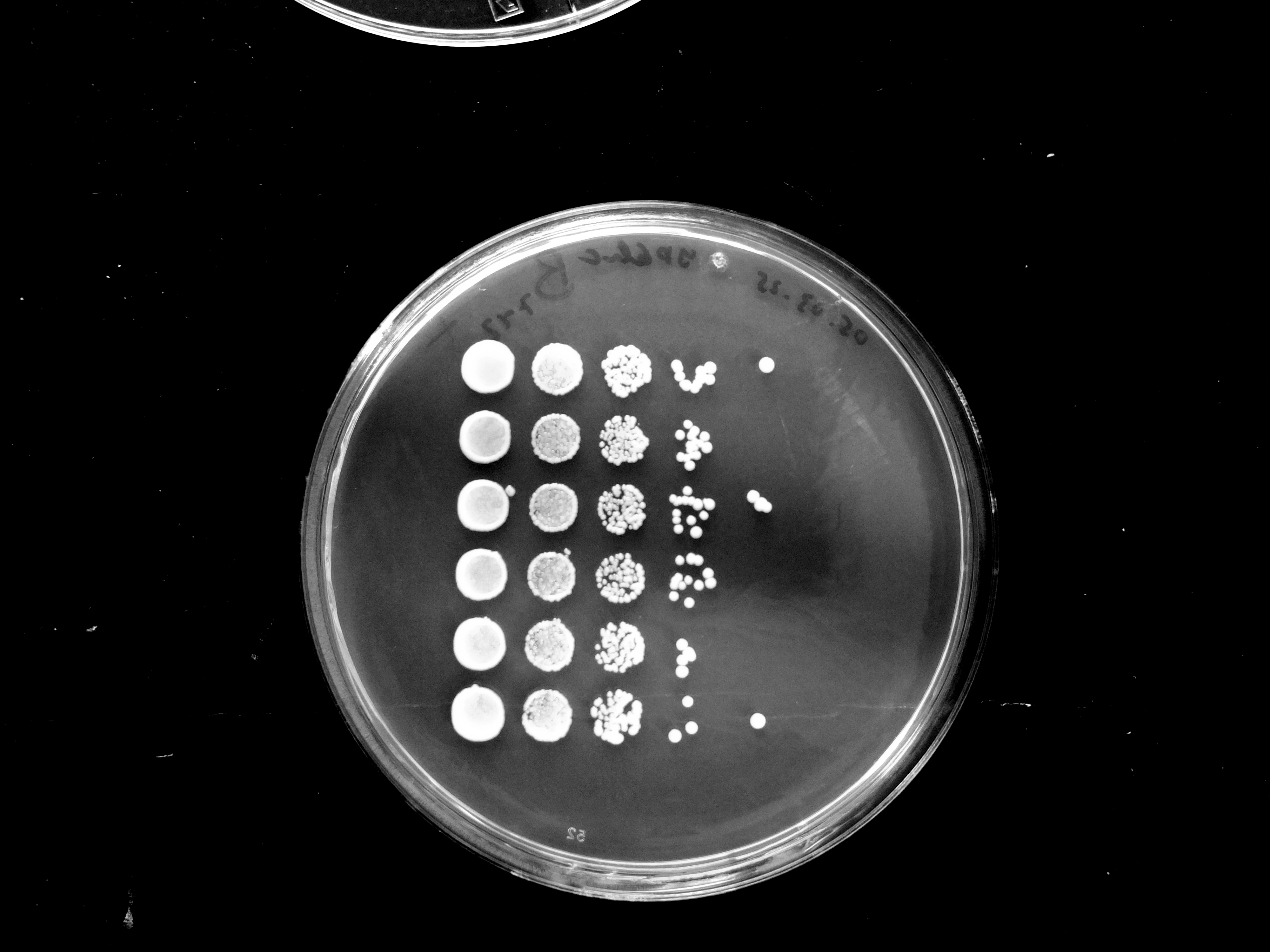

Supplement: Supplementary file 4 — Source data Fig. 2 [file 44318_2025_459_MOESM4_ESM.zip › Fig2/D/middle/DSCF8211-2.tif]

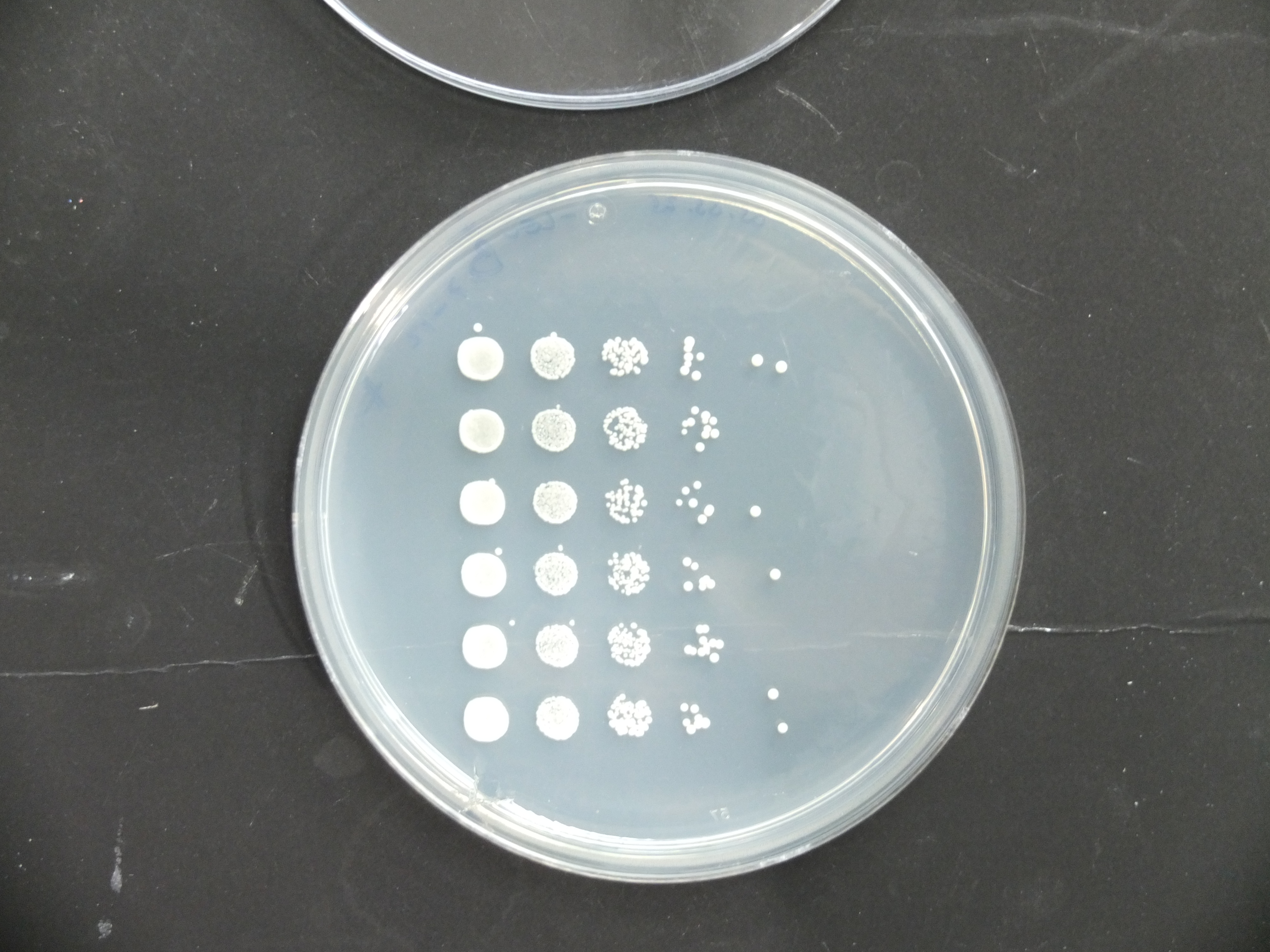

Supplement: Supplementary file 4 — Source data Fig. 2 [file 44318_2025_459_MOESM4_ESM.zip › Fig2/D/middle/DSCF8233.JPG]

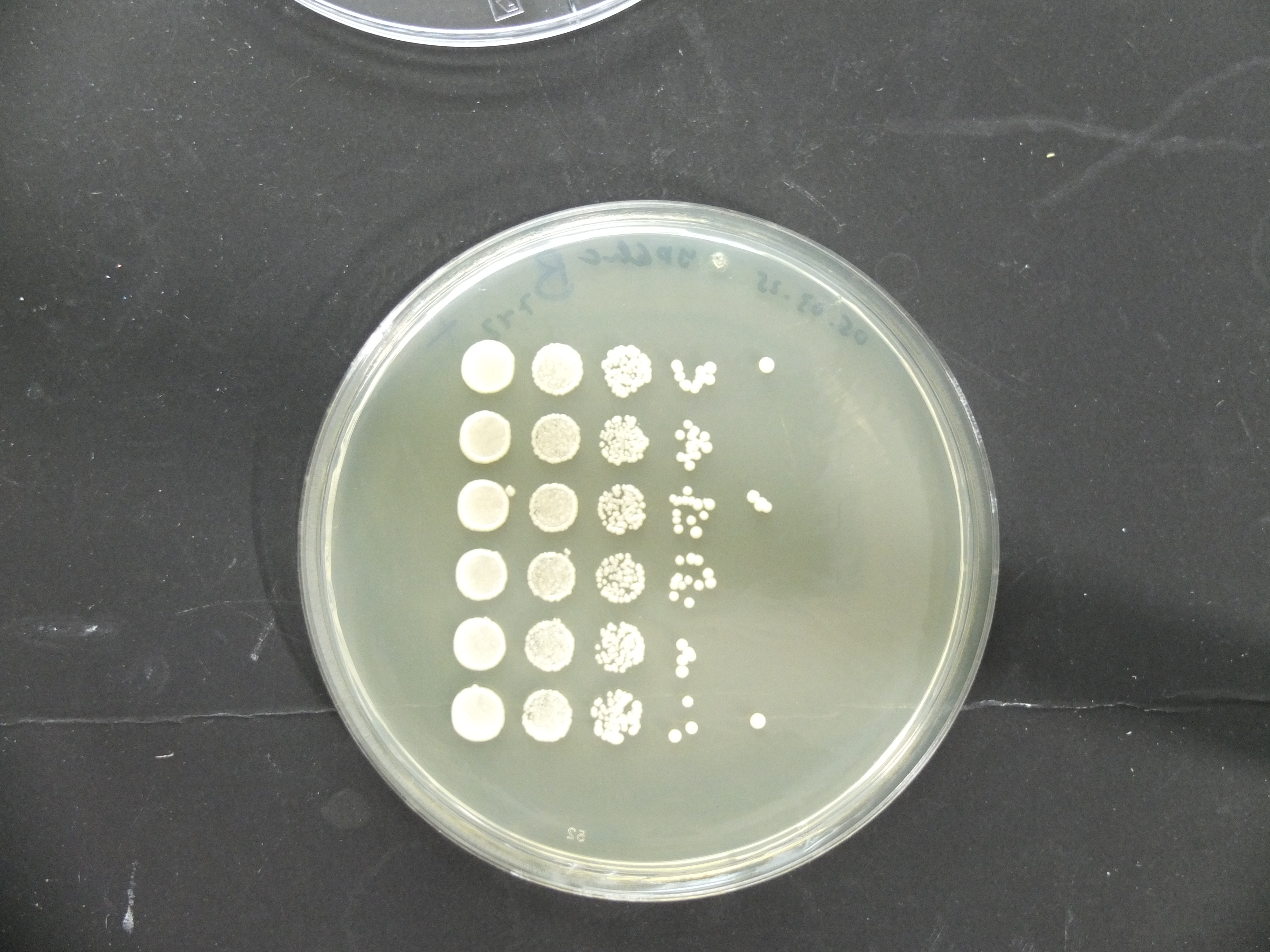

Supplement: Supplementary file 4 — Source data Fig. 2 [file 44318_2025_459_MOESM4_ESM.zip › Fig2/D/middle/DSCF8211.JPG]

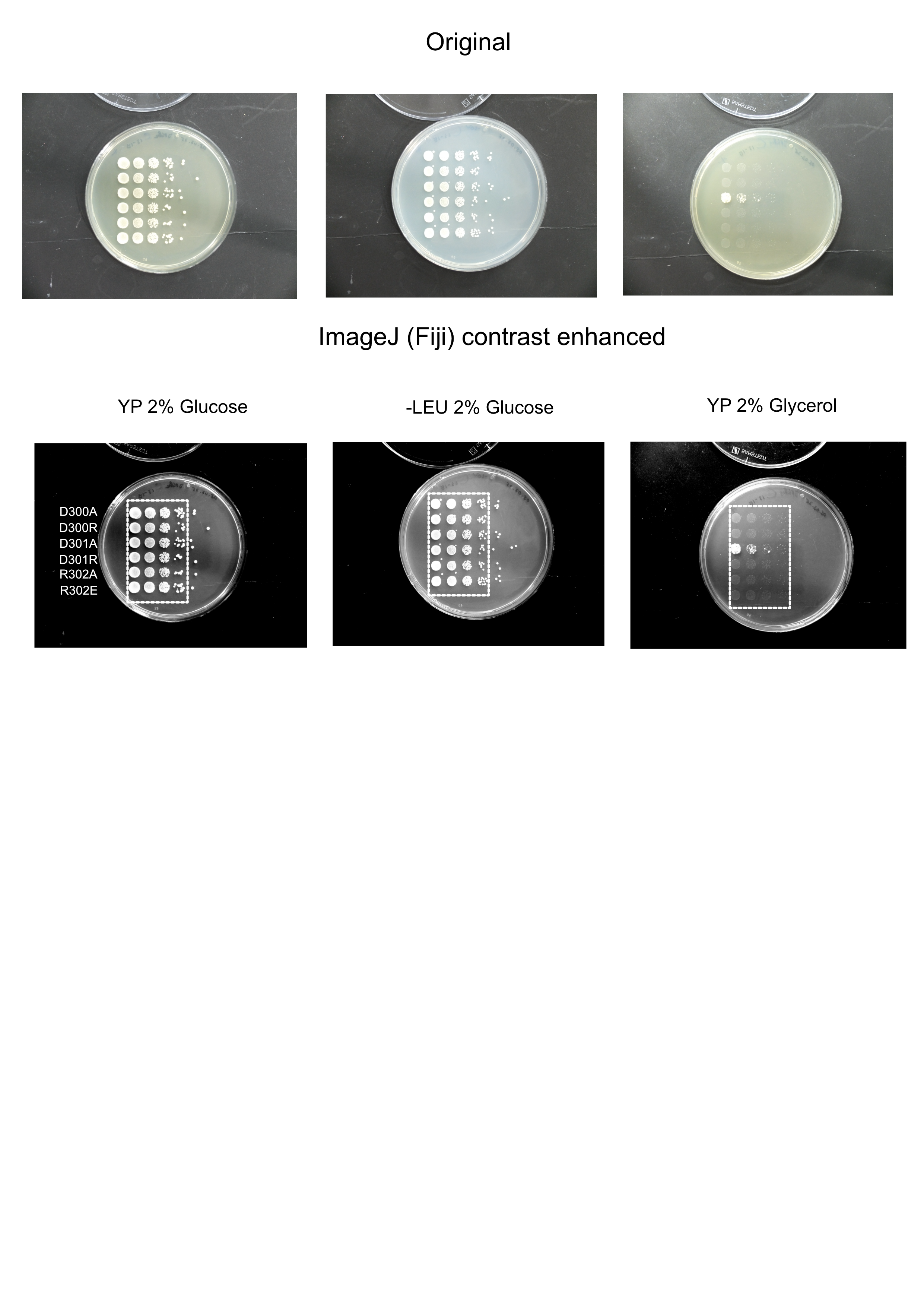

Supplement: Supplementary file 4 — Source data Fig. 2 [file 44318_2025_459_MOESM4_ESM.zip › Fig2/D/bottom/Fig2Dbottom_source.png]

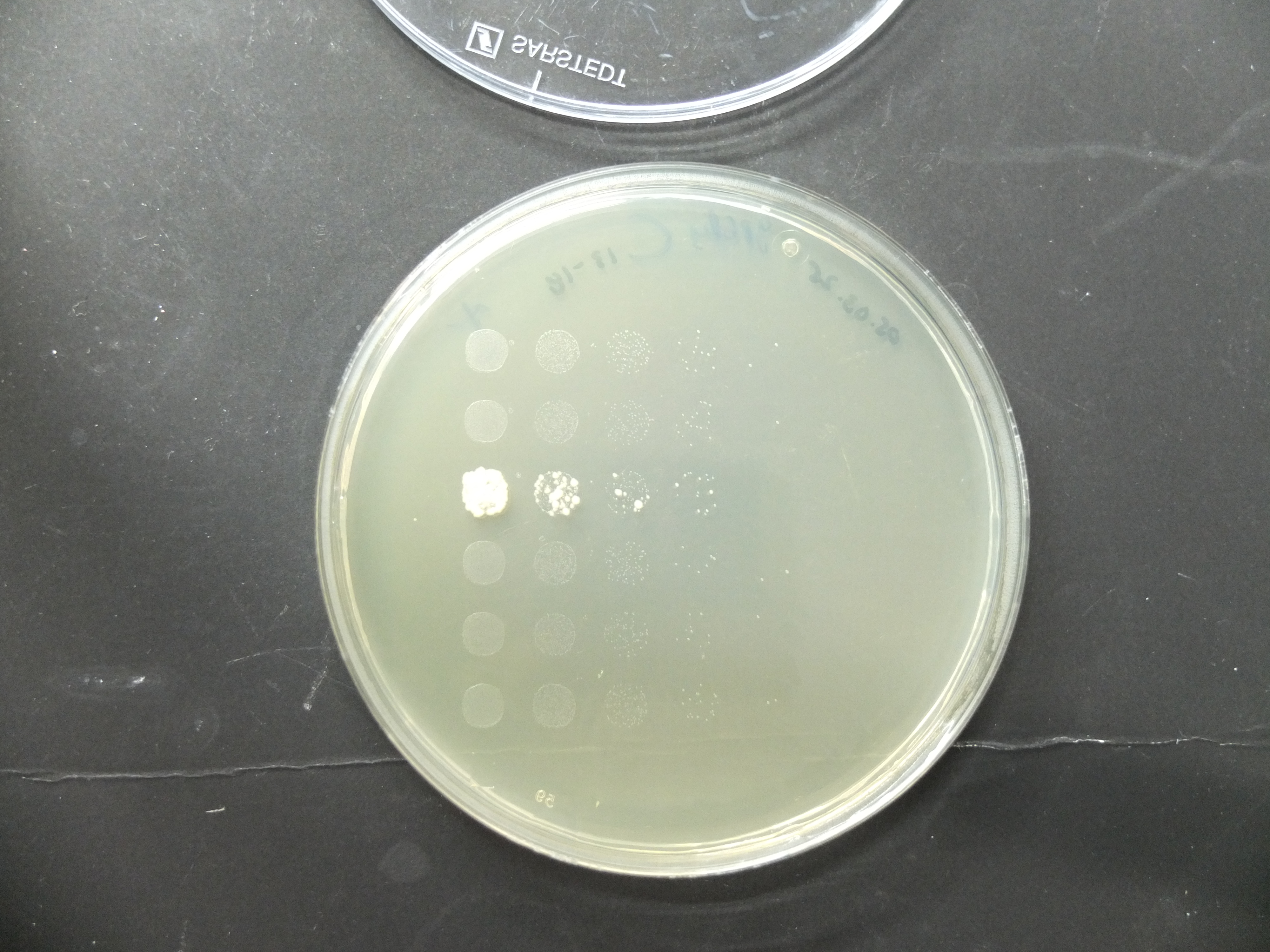

Supplement: Supplementary file 4 — Source data Fig. 2 [file 44318_2025_459_MOESM4_ESM.zip › Fig2/D/bottom/DSCF8257.JPG]

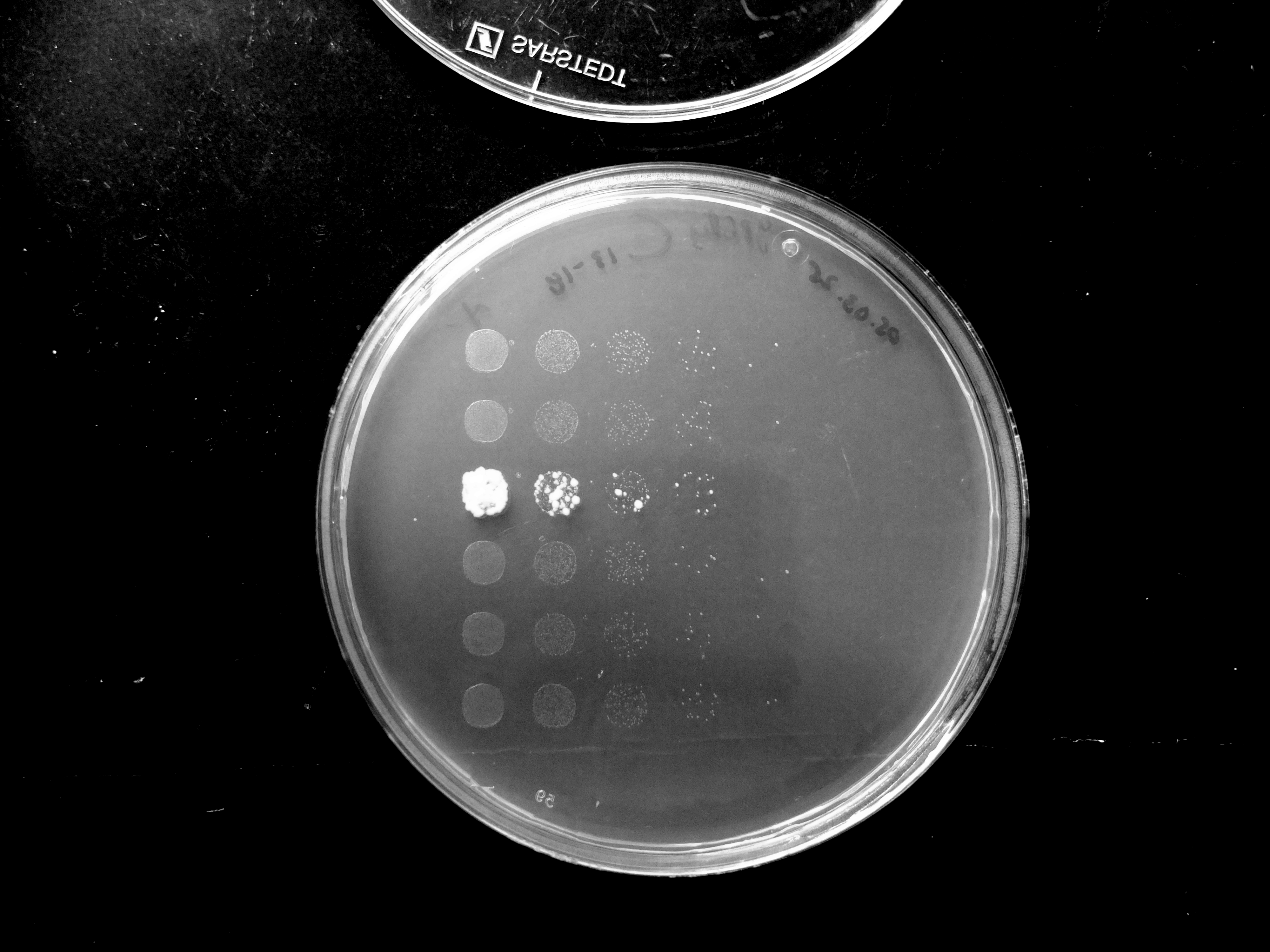

Supplement: Supplementary file 4 — Source data Fig. 2 [file 44318_2025_459_MOESM4_ESM.zip › Fig2/D/bottom/DSCF8257-2.tif]

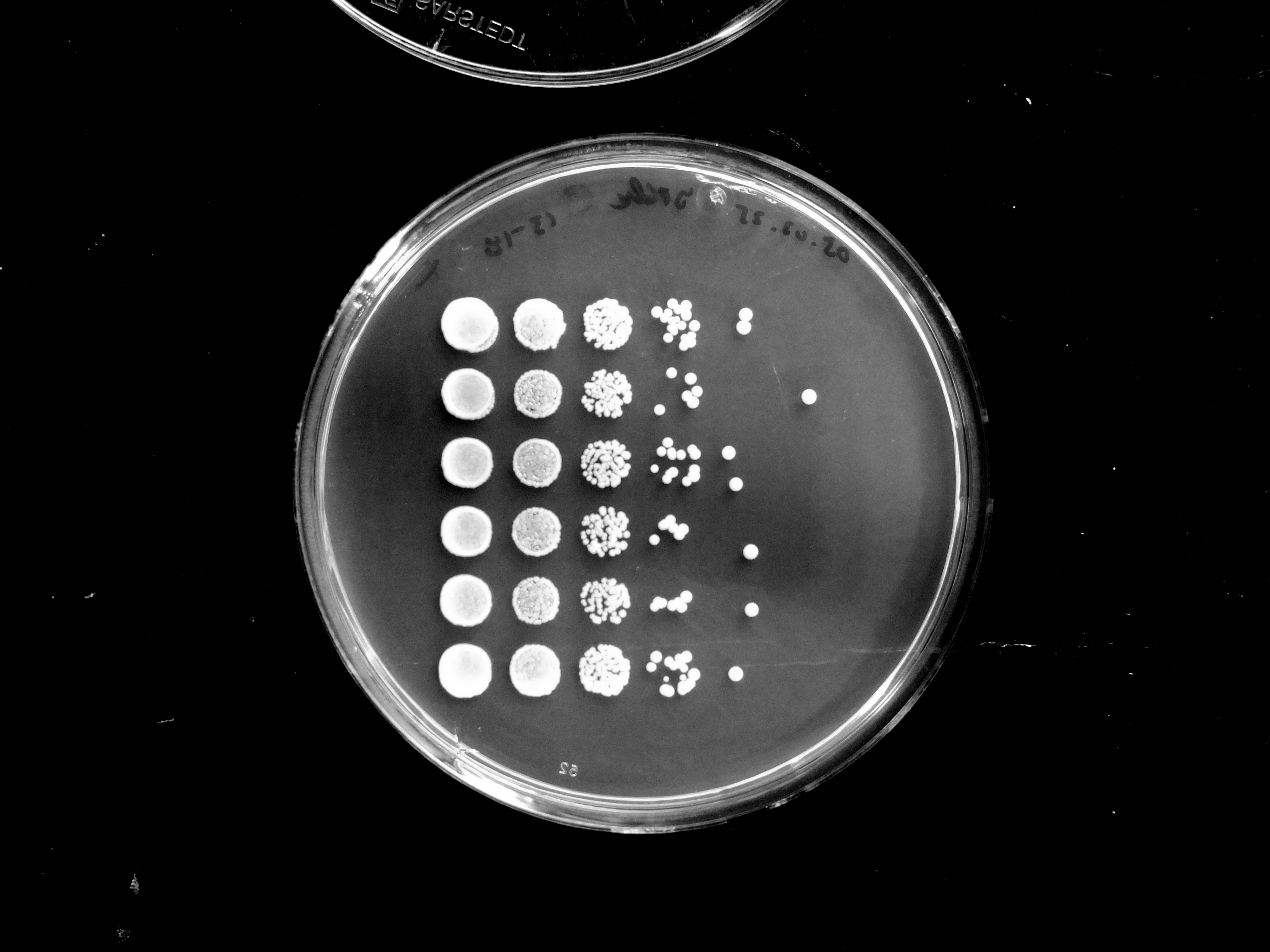

Supplement: Supplementary file 4 — Source data Fig. 2 [file 44318_2025_459_MOESM4_ESM.zip › Fig2/D/bottom/DSCF8217-2.tif]

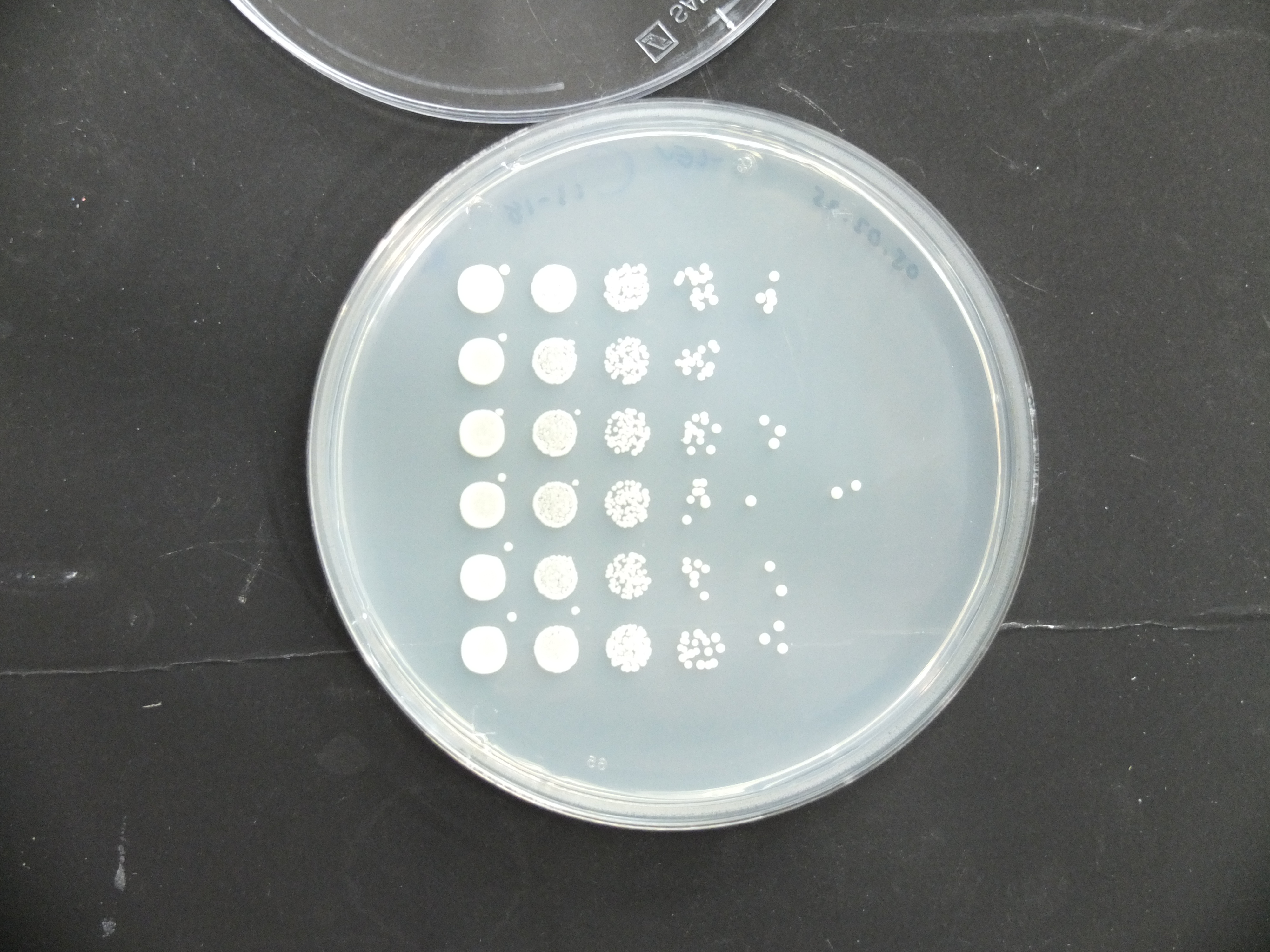

Supplement: Supplementary file 4 — Source data Fig. 2 [file 44318_2025_459_MOESM4_ESM.zip › Fig2/D/bottom/DSCF8235.JPG]

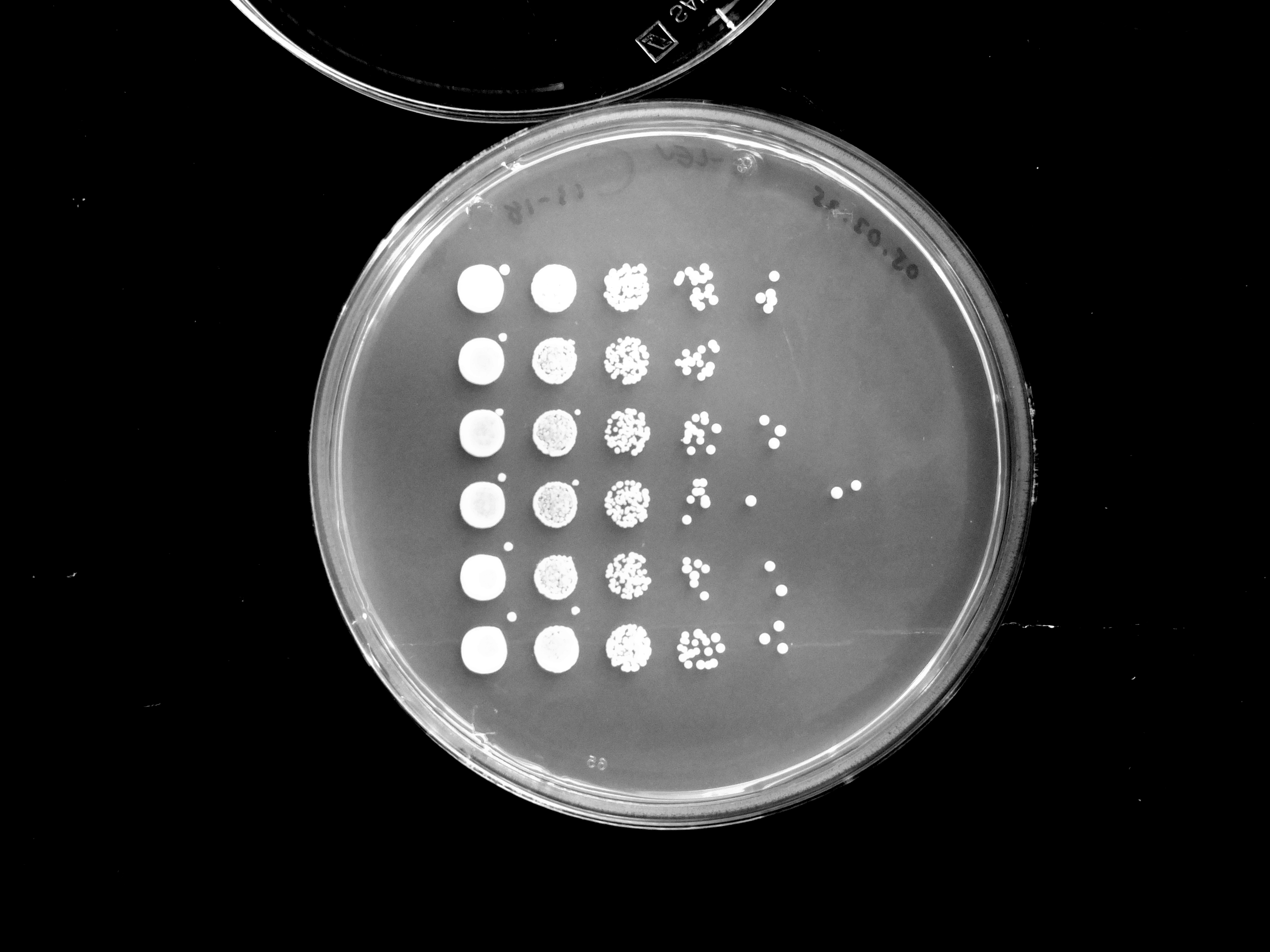

Supplement: Supplementary file 4 — Source data Fig. 2 [file 44318_2025_459_MOESM4_ESM.zip › Fig2/D/bottom/DSCF8235-2.tif]

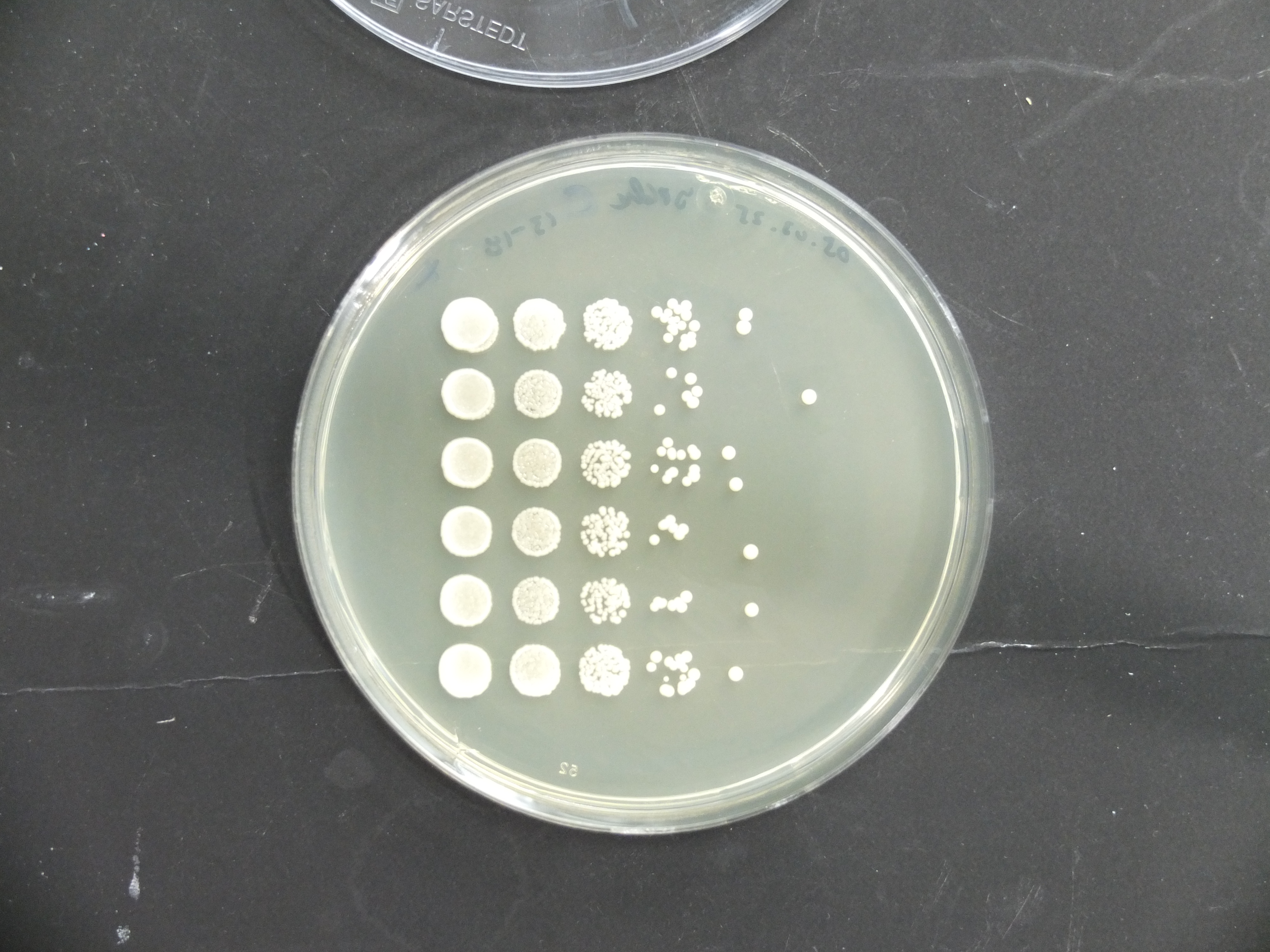

Supplement: Supplementary file 4 — Source data Fig. 2 [file 44318_2025_459_MOESM4_ESM.zip › Fig2/D/bottom/DSCF8217.JPG]

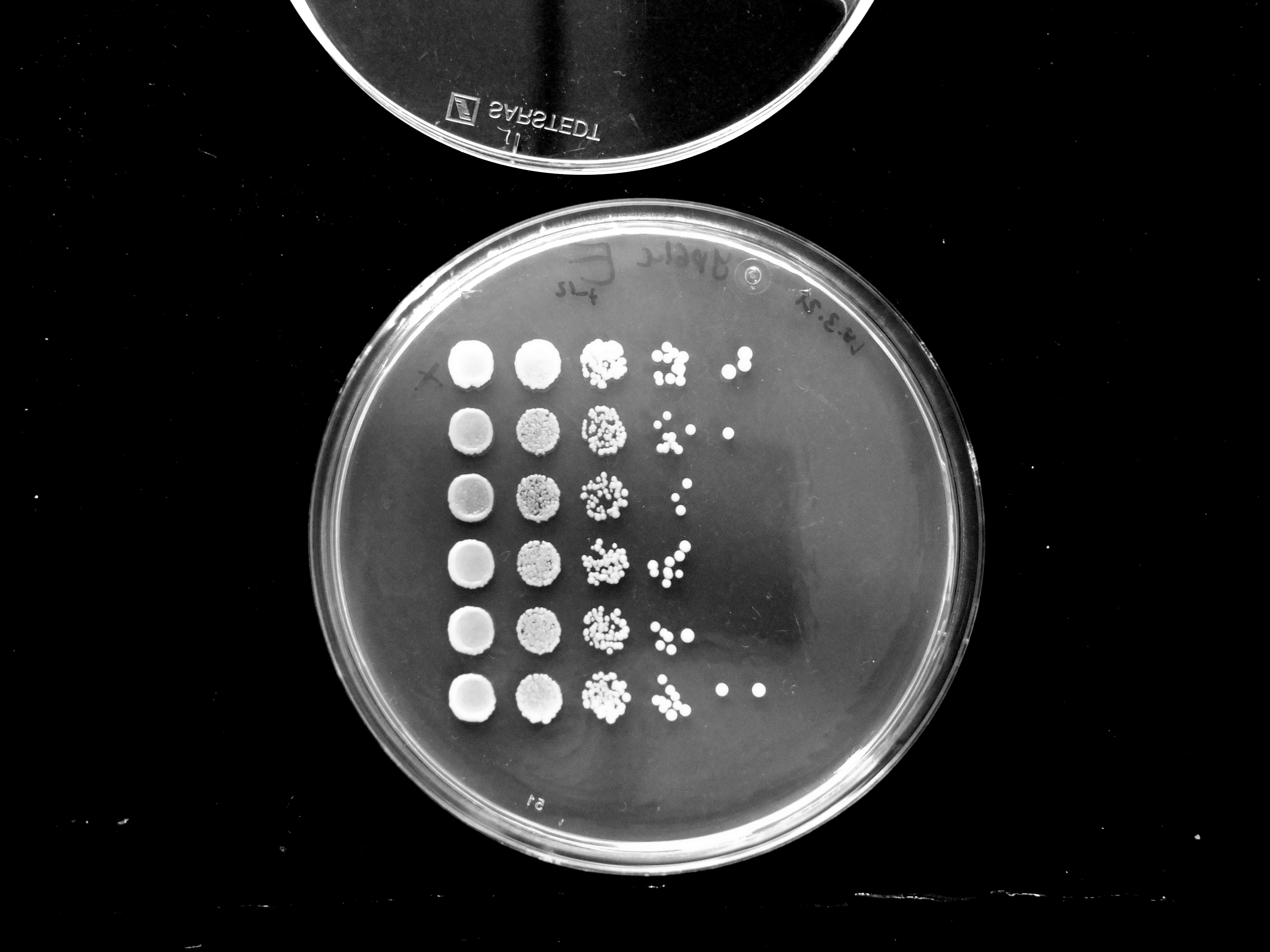

Supplement: Supplementary file 4 — Source data Fig. 2 [file 44318_2025_459_MOESM4_ESM.zip › Fig2/E/top/DSCF8291-2.tif]

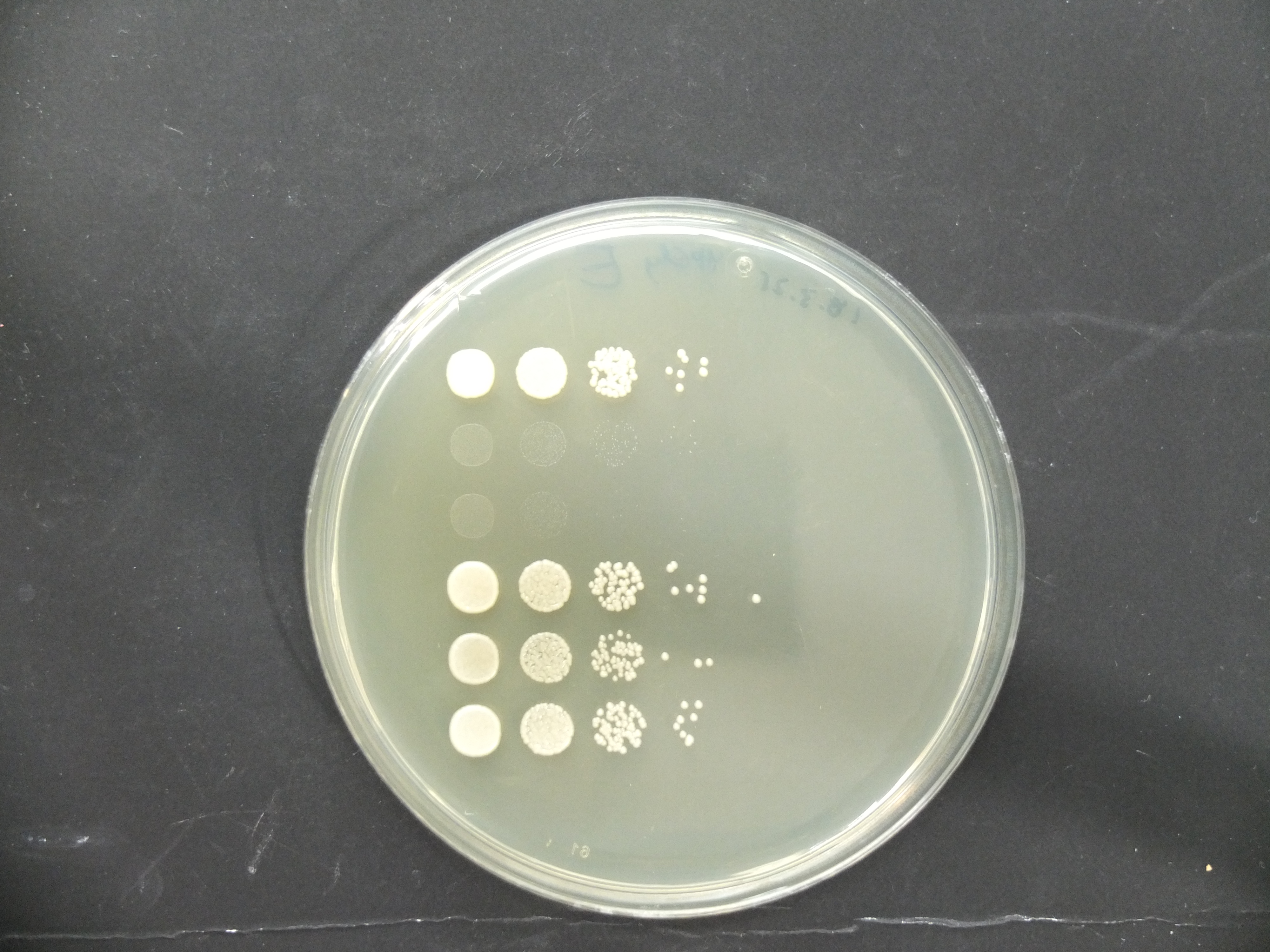

Supplement: Supplementary file 4 — Source data Fig. 2 [file 44318_2025_459_MOESM4_ESM.zip › Fig2/E/top/DSCF8322.JPG]

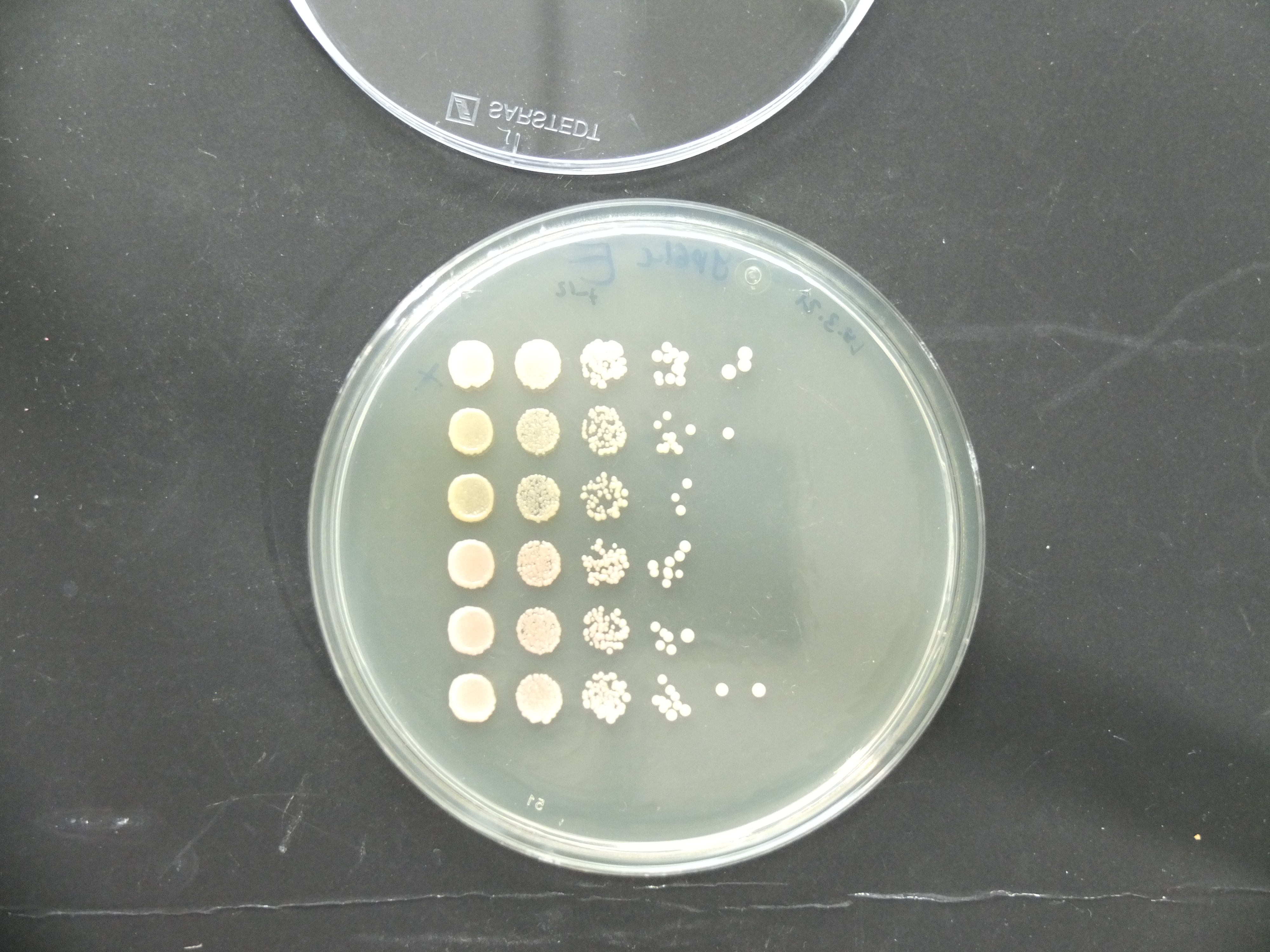

Supplement: Supplementary file 4 — Source data Fig. 2 [file 44318_2025_459_MOESM4_ESM.zip › Fig2/E/top/DSCF8291.JPG]

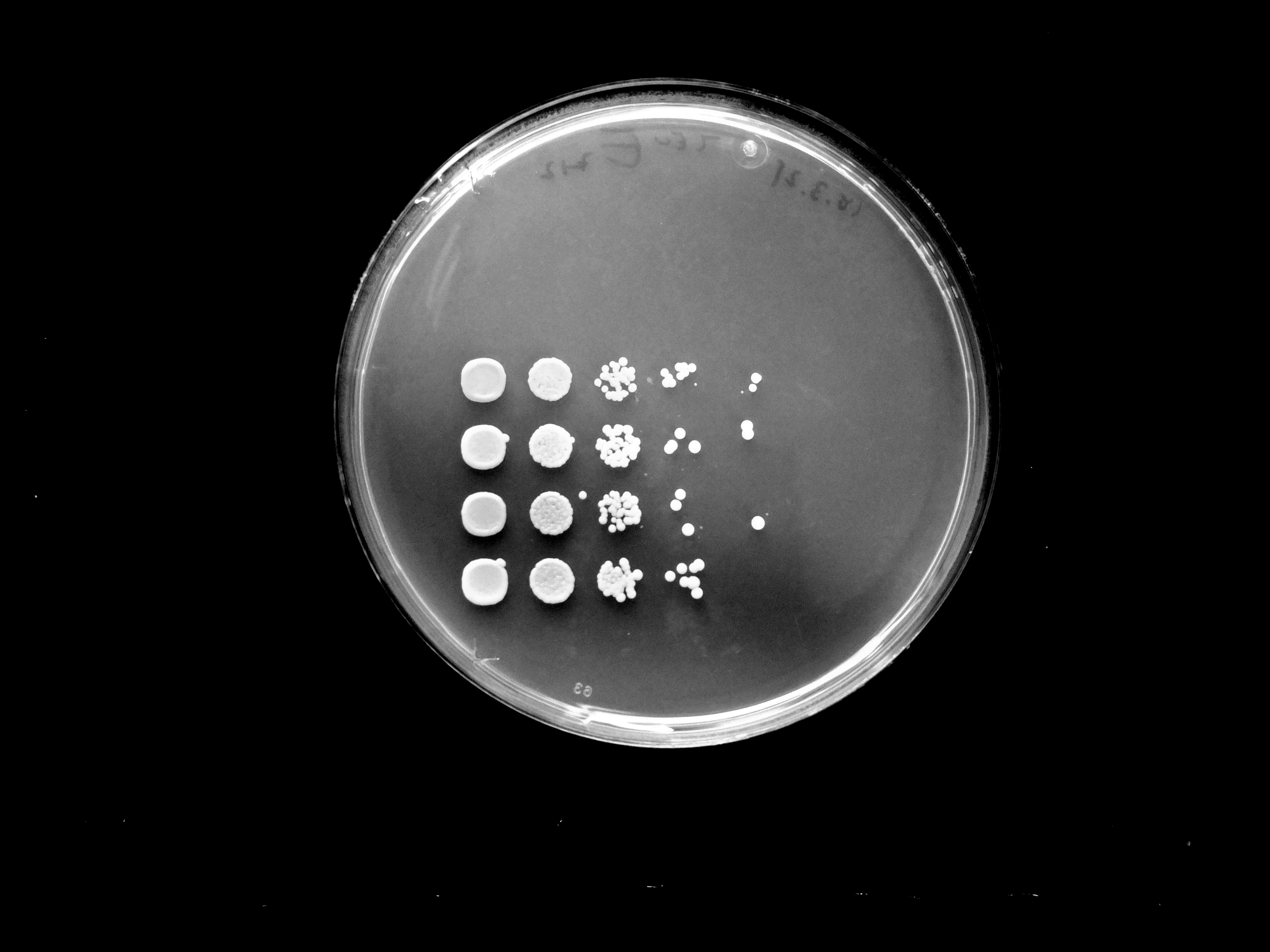

Supplement: Supplementary file 4 — Source data Fig. 2 [file 44318_2025_459_MOESM4_ESM.zip › Fig2/E/top/DSCF8302-2.tif]

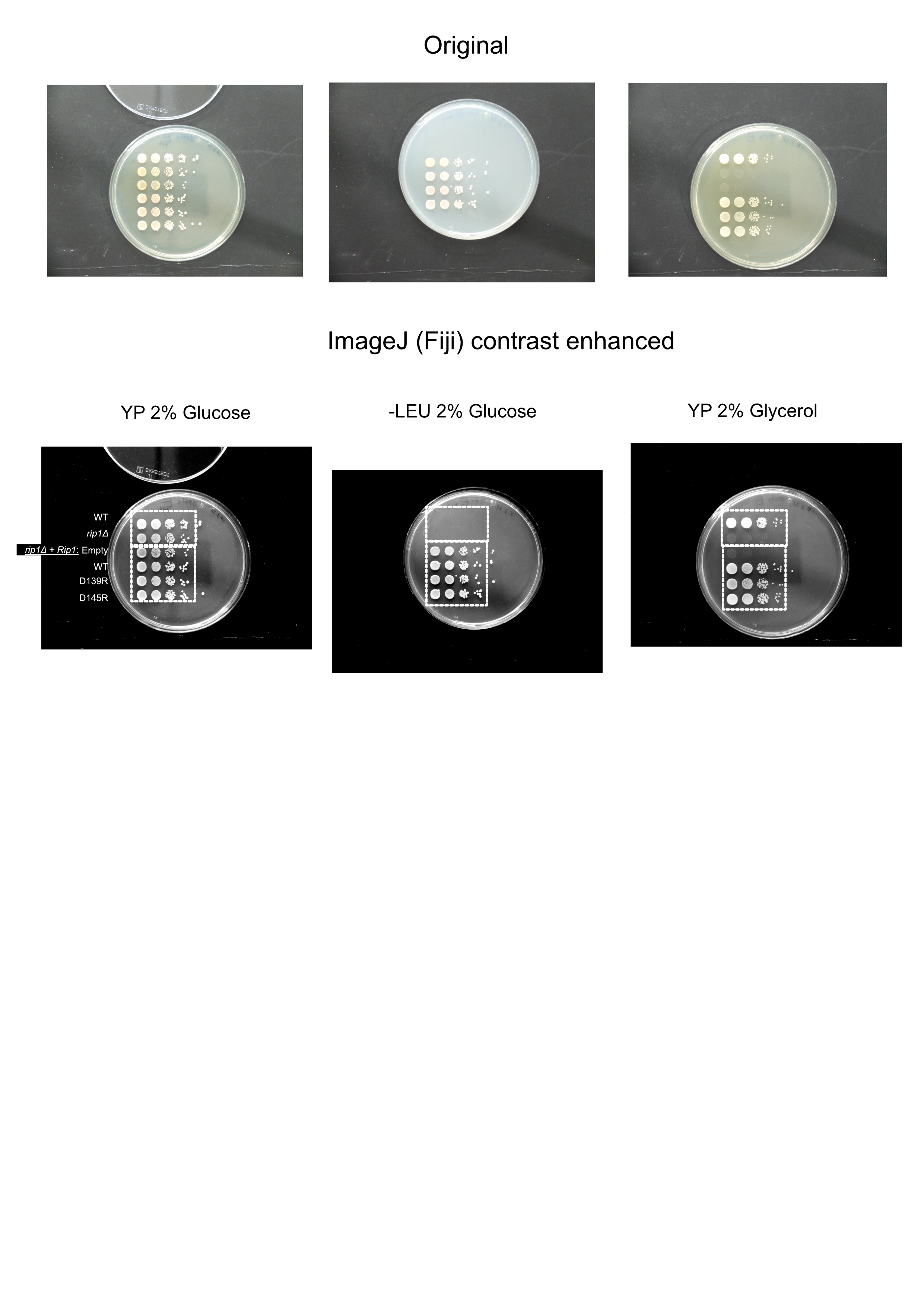

Supplement: Supplementary file 4 — Source data Fig. 2 [file 44318_2025_459_MOESM4_ESM.zip › Fig2/E/top/Fig2Etop_source.png]

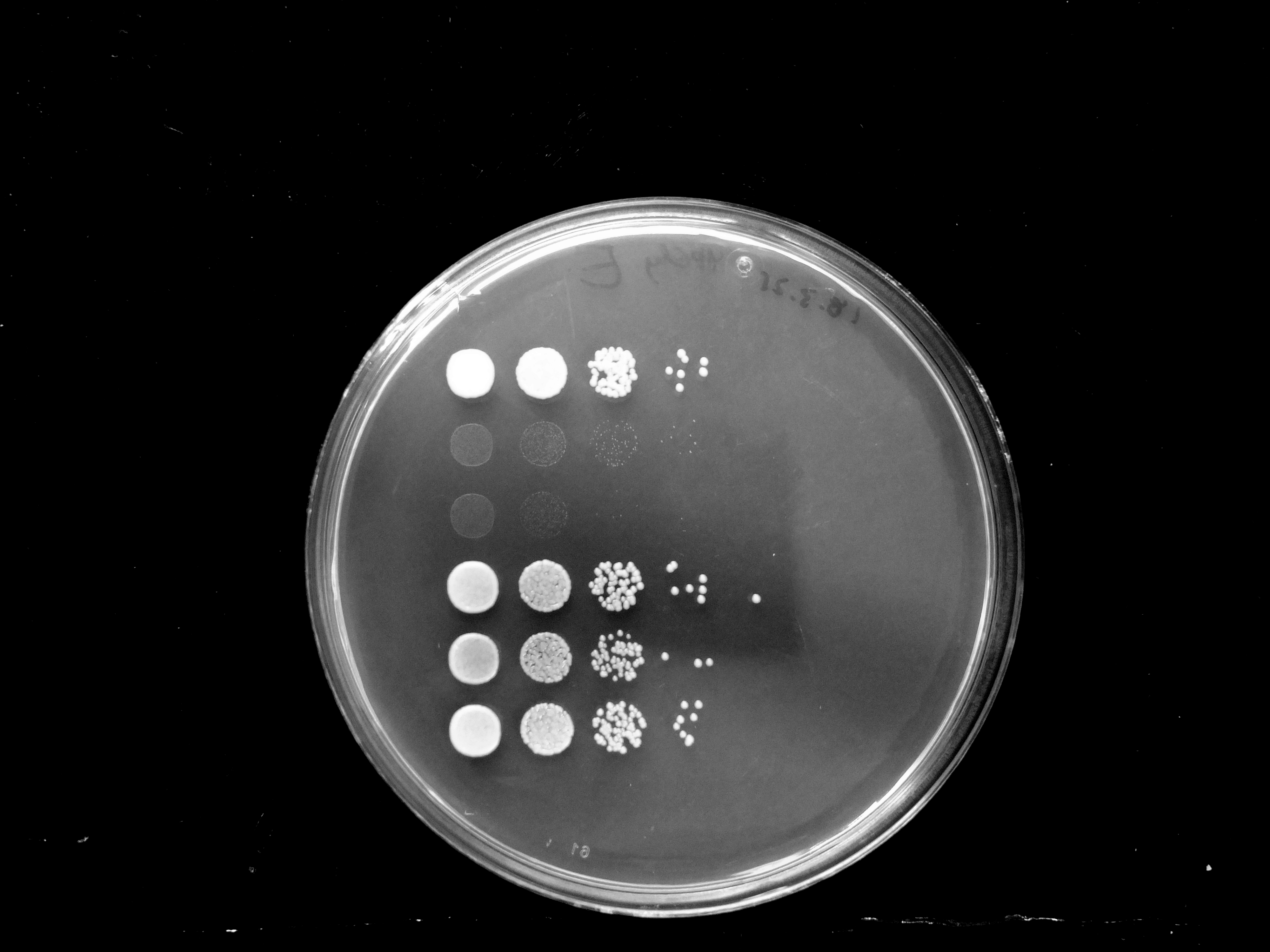

Supplement: Supplementary file 4 — Source data Fig. 2 [file 44318_2025_459_MOESM4_ESM.zip › Fig2/E/top/DSCF8322-2.tif]

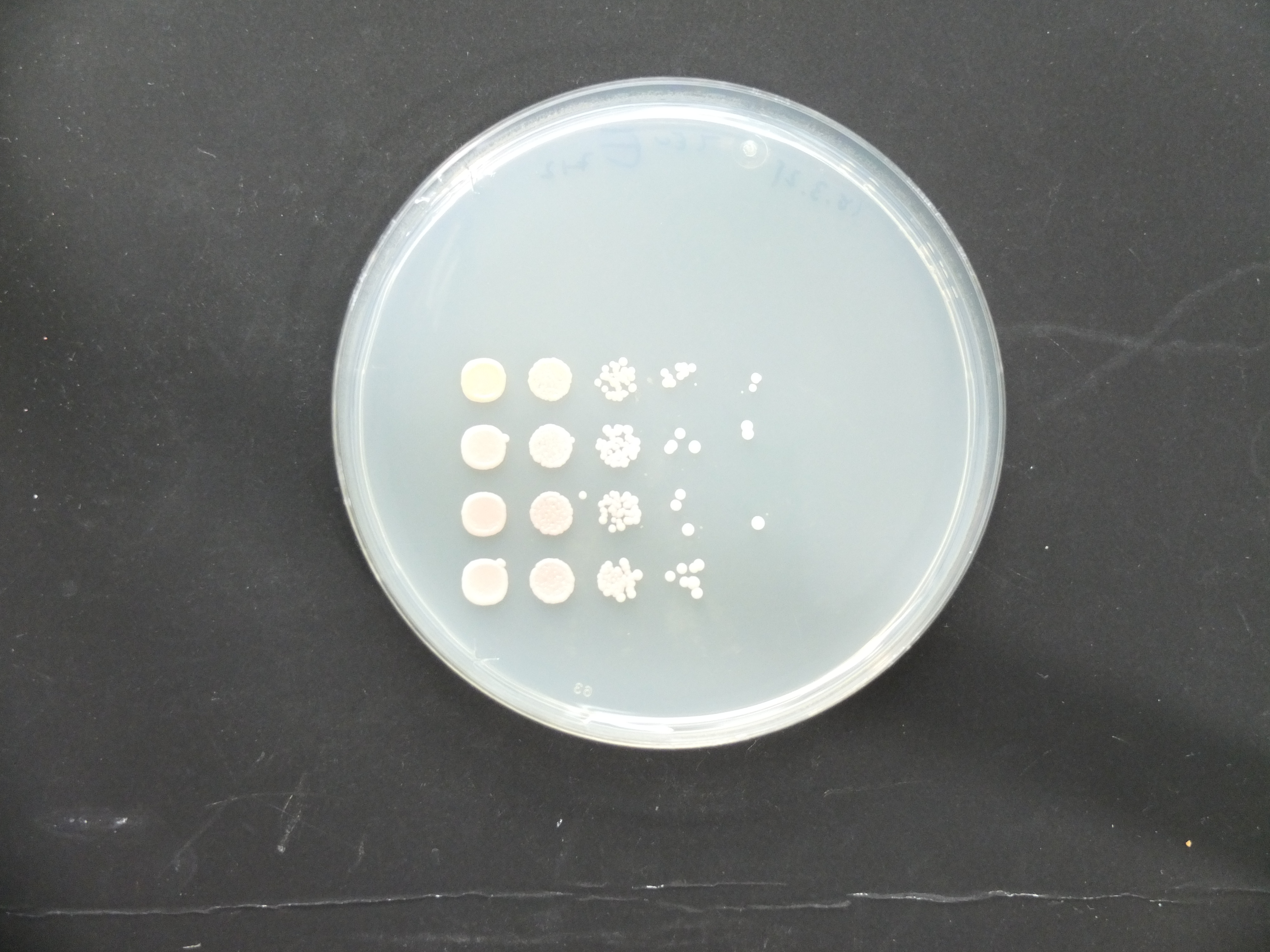

Supplement: Supplementary file 4 — Source data Fig. 2 [file 44318_2025_459_MOESM4_ESM.zip › Fig2/E/top/DSCF8302.JPG]

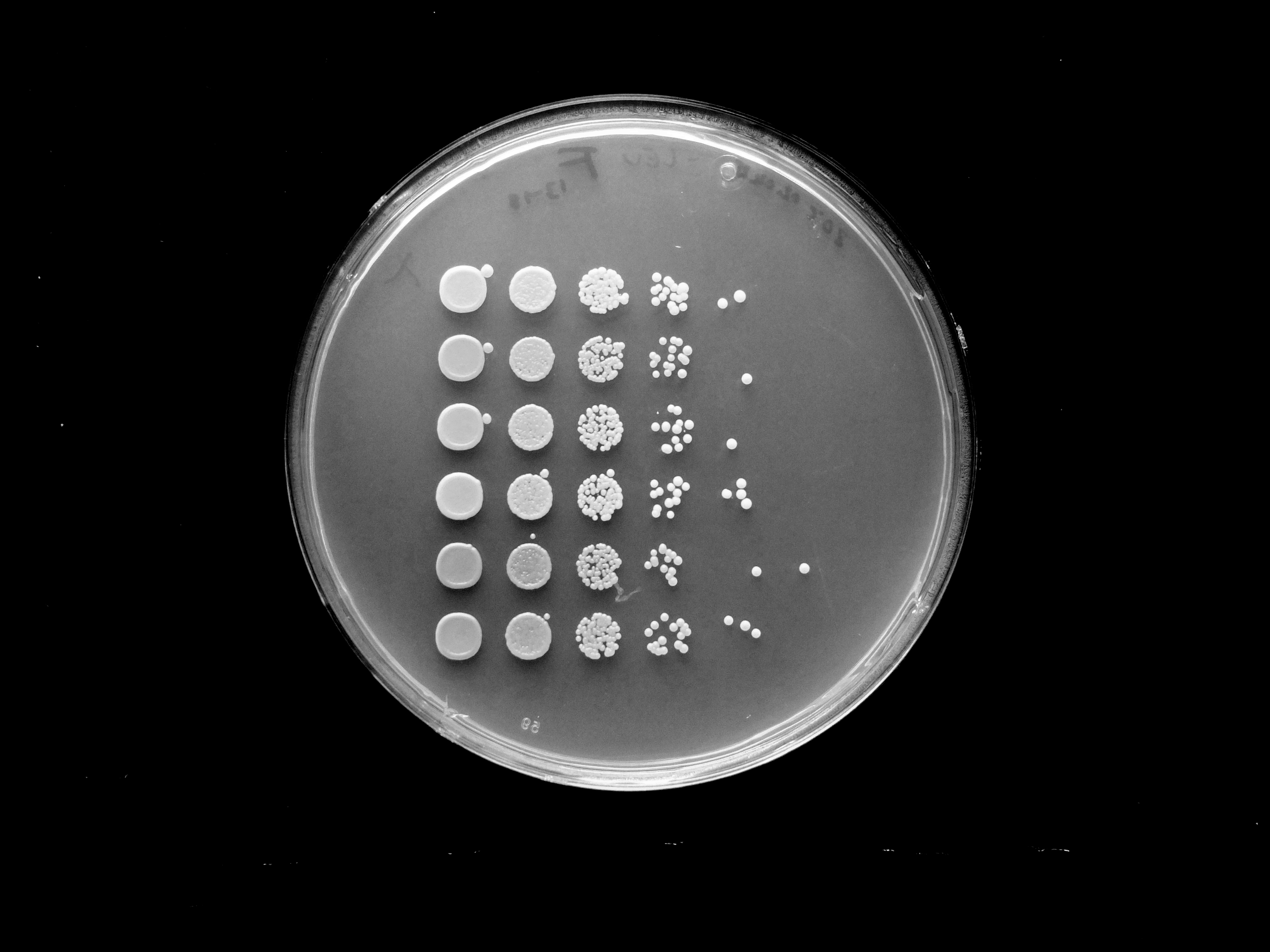

Supplement: Supplementary file 4 — Source data Fig. 2 [file 44318_2025_459_MOESM4_ESM.zip › Fig2/E/middle/DSCF8353.tif]

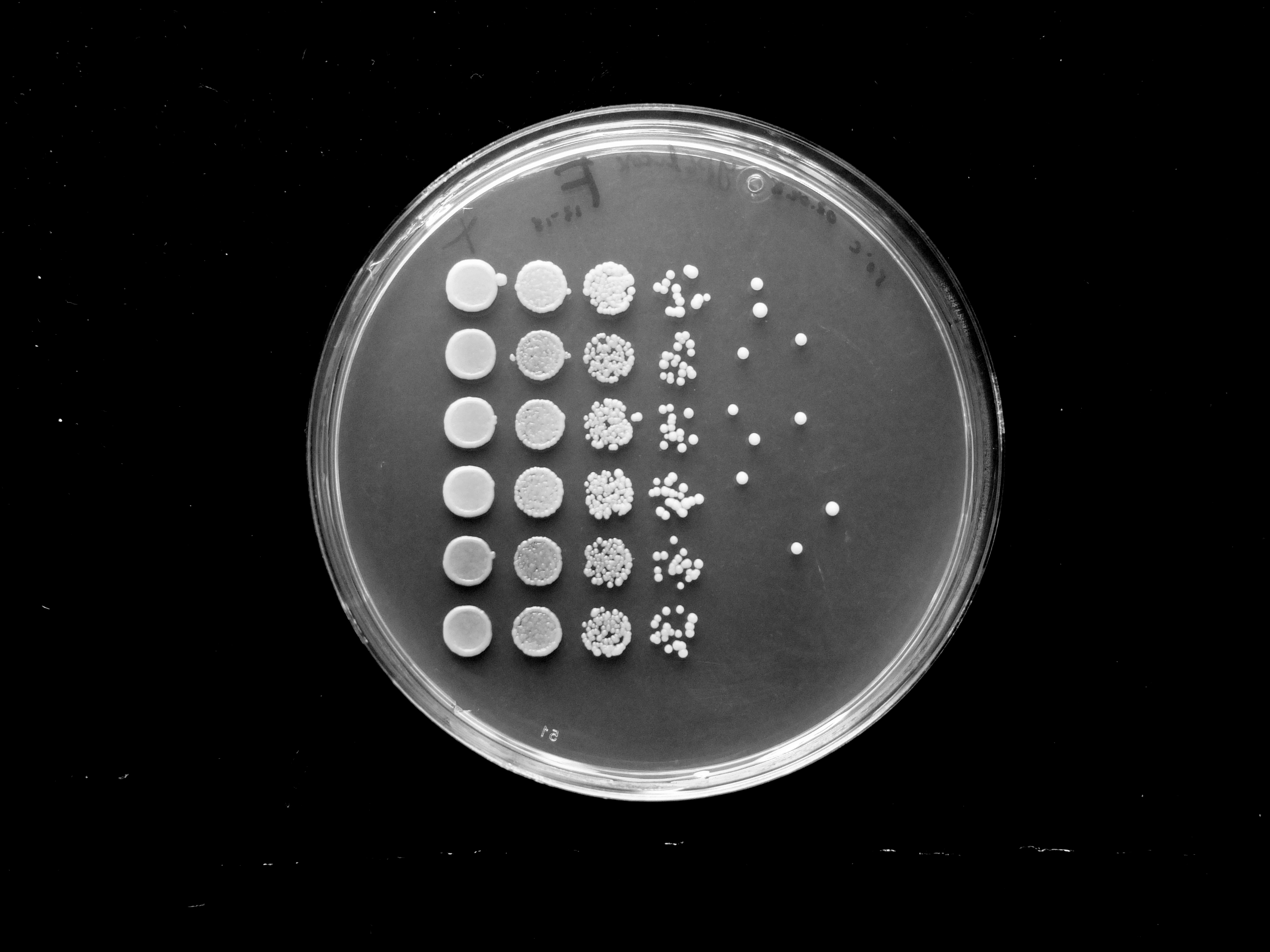

Supplement: Supplementary file 4 — Source data Fig. 2 [file 44318_2025_459_MOESM4_ESM.zip › Fig2/E/middle/DSCF8343.tif]

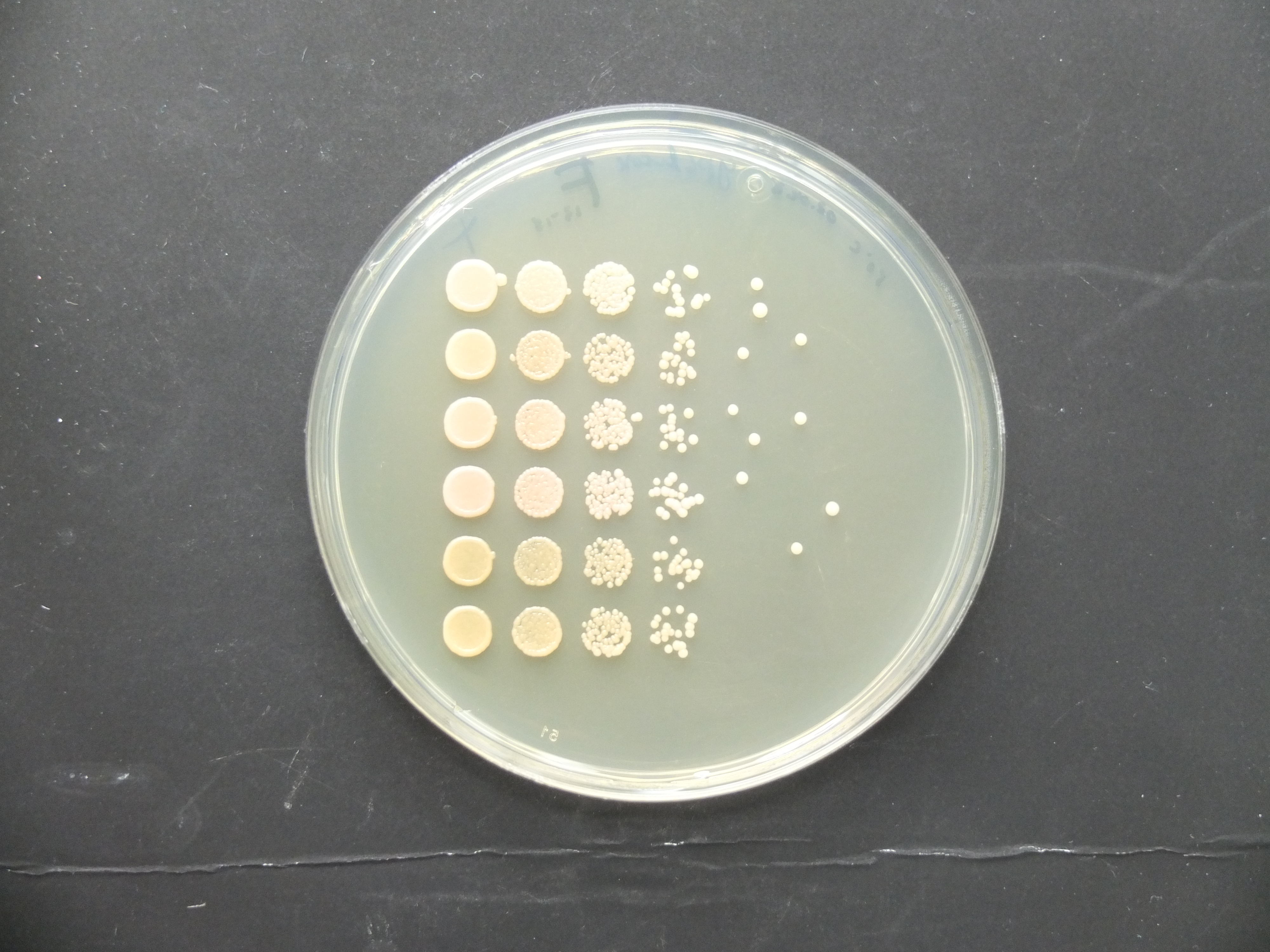

Supplement: Supplementary file 4 — Source data Fig. 2 [file 44318_2025_459_MOESM4_ESM.zip › Fig2/E/middle/DSCF8343.JPG]

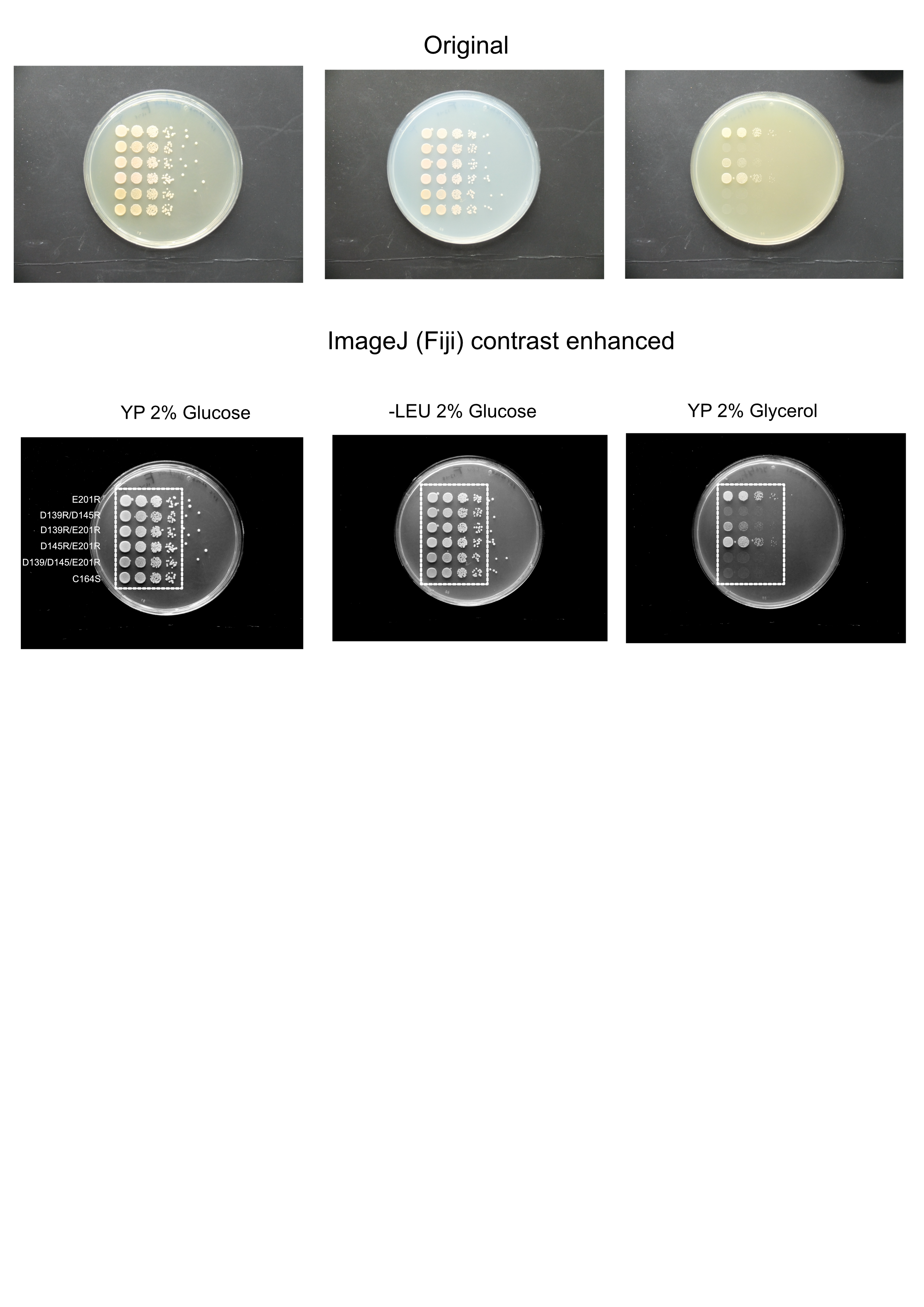

Supplement: Supplementary file 4 — Source data Fig. 2 [file 44318_2025_459_MOESM4_ESM.zip › Fig2/E/middle/Fig2Emiddle_source.png]

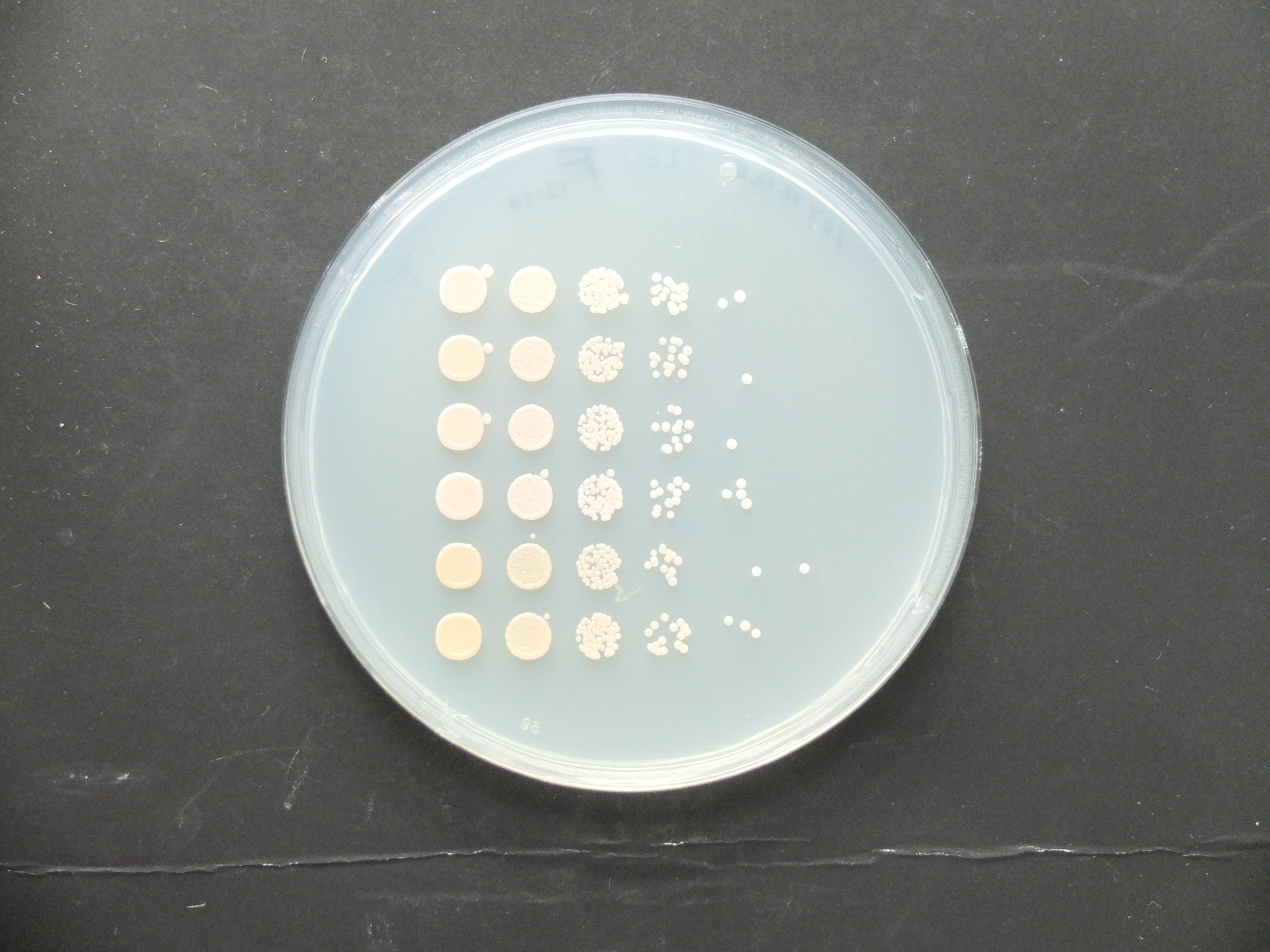

Supplement: Supplementary file 4 — Source data Fig. 2 [file 44318_2025_459_MOESM4_ESM.zip › Fig2/E/middle/DSCF8353.JPG]

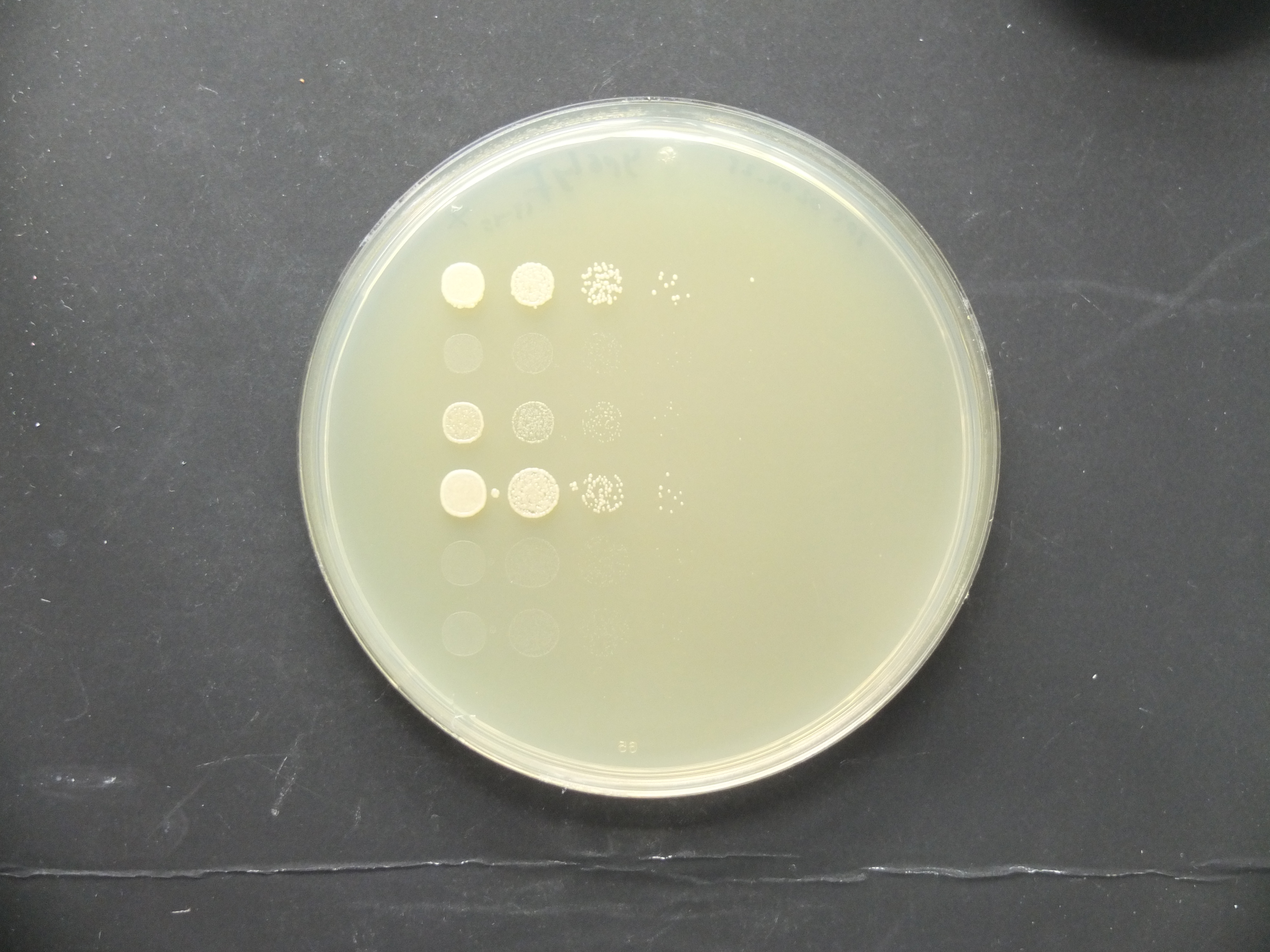

Supplement: Supplementary file 4 — Source data Fig. 2 [file 44318_2025_459_MOESM4_ESM.zip › Fig2/E/middle/DSCF8389.JPG]

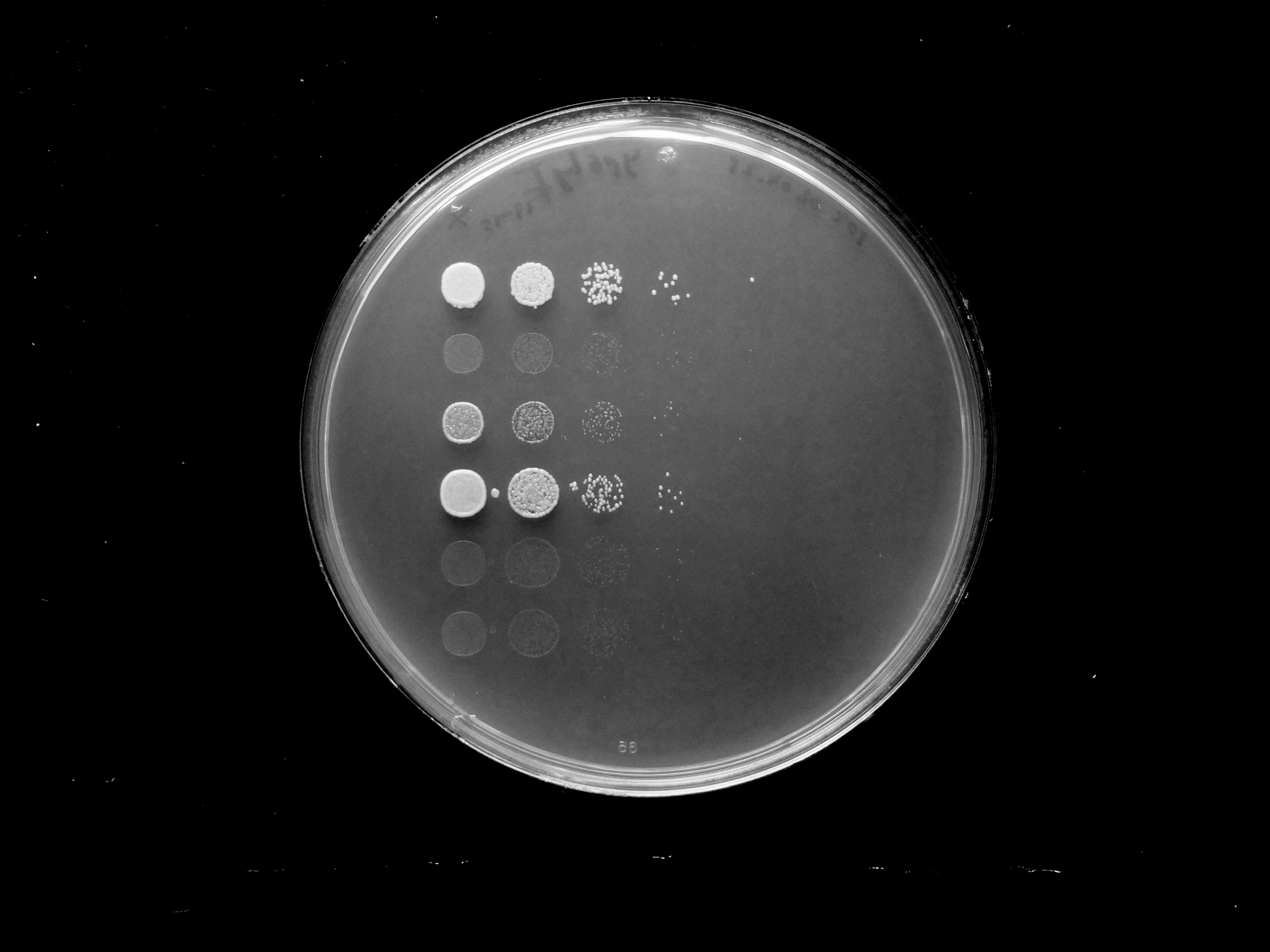

Supplement: Supplementary file 4 — Source data Fig. 2 [file 44318_2025_459_MOESM4_ESM.zip › Fig2/E/middle/DSCF8389.tif]

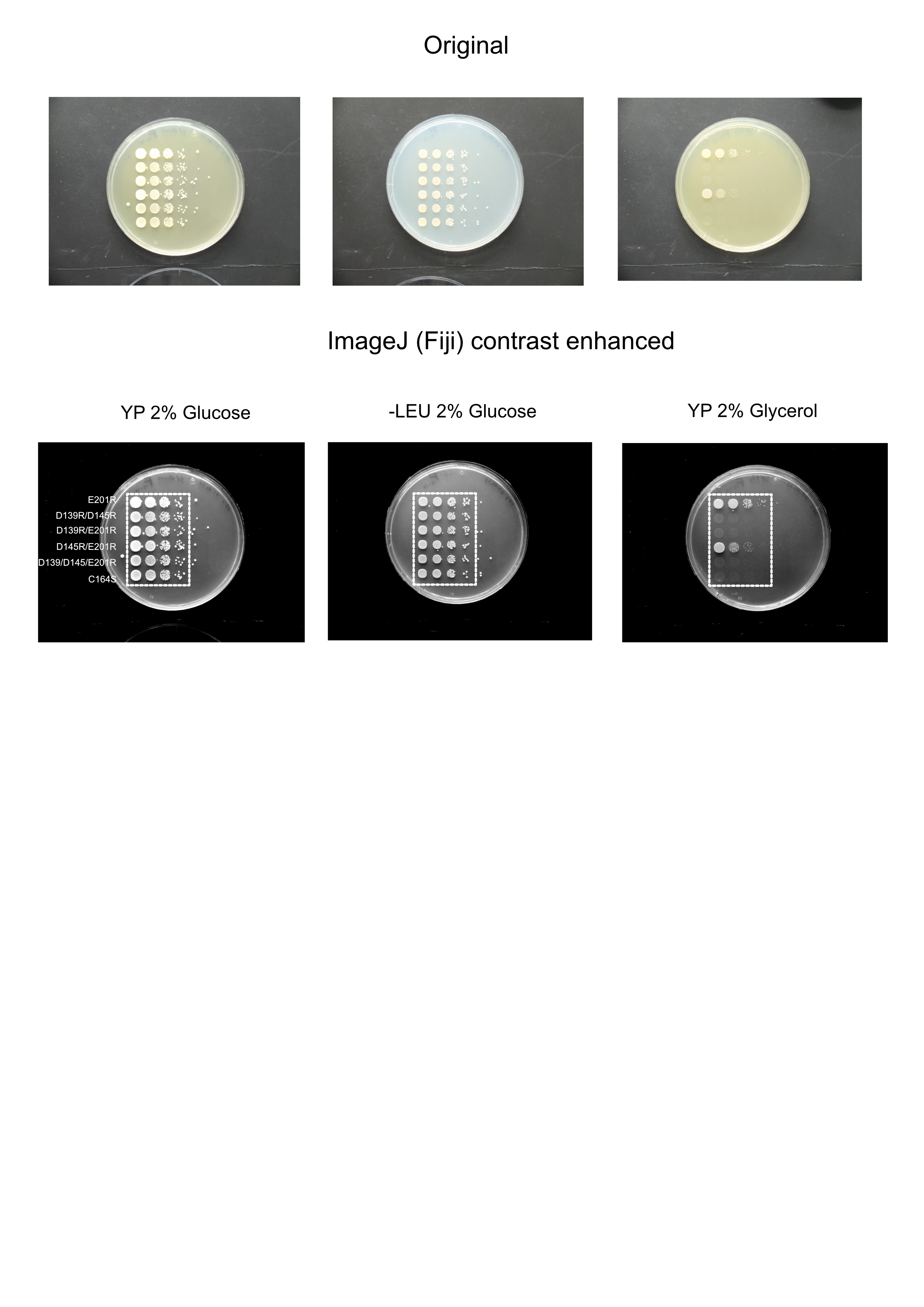

Supplement: Supplementary file 4 — Source data Fig. 2 [file 44318_2025_459_MOESM4_ESM.zip › Fig2/E/bottom/Fig2Ebottom_source.png]

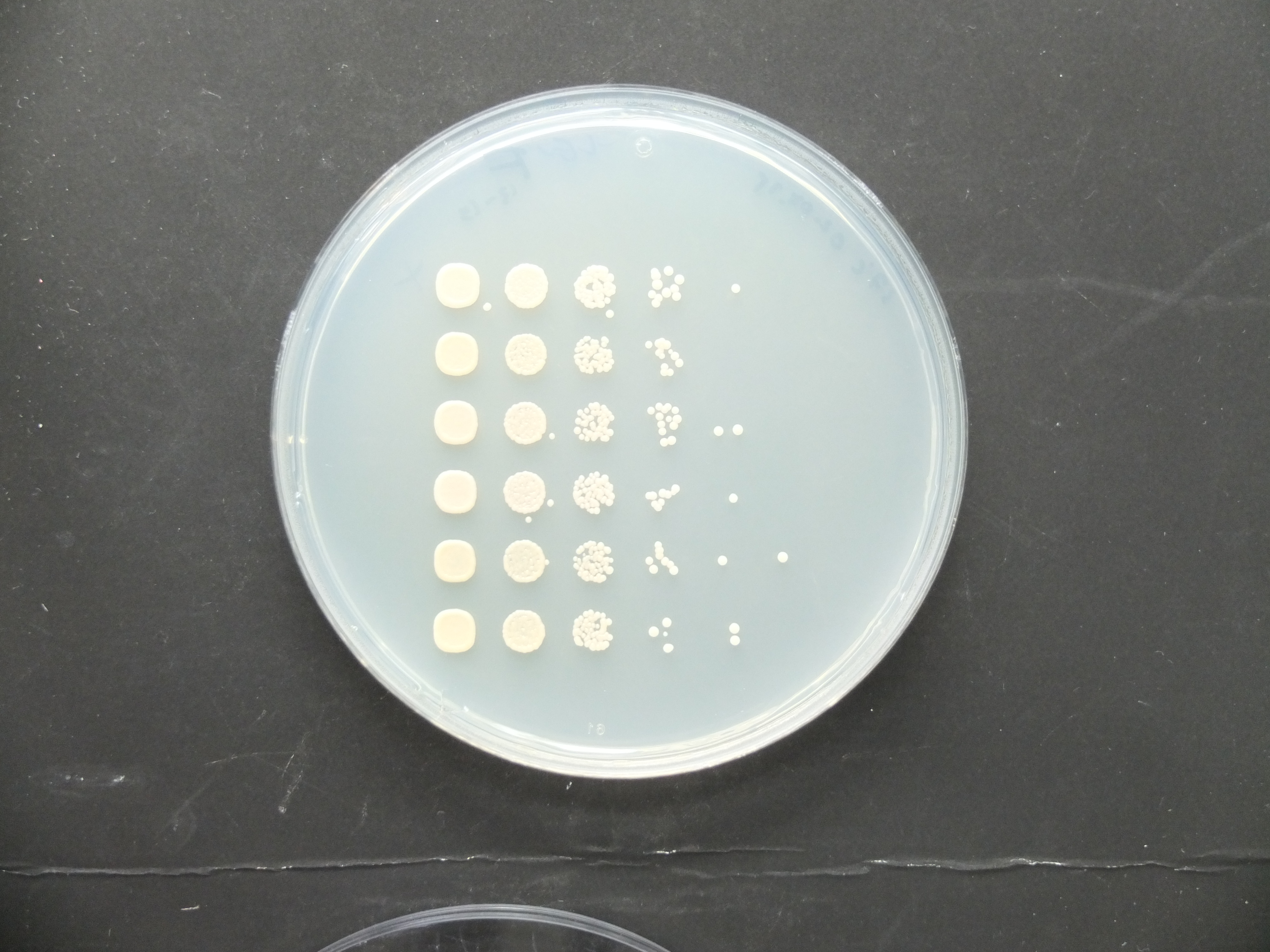

Supplement: Supplementary file 4 — Source data Fig. 2 [file 44318_2025_459_MOESM4_ESM.zip › Fig2/E/bottom/DSCF8377.JPG]

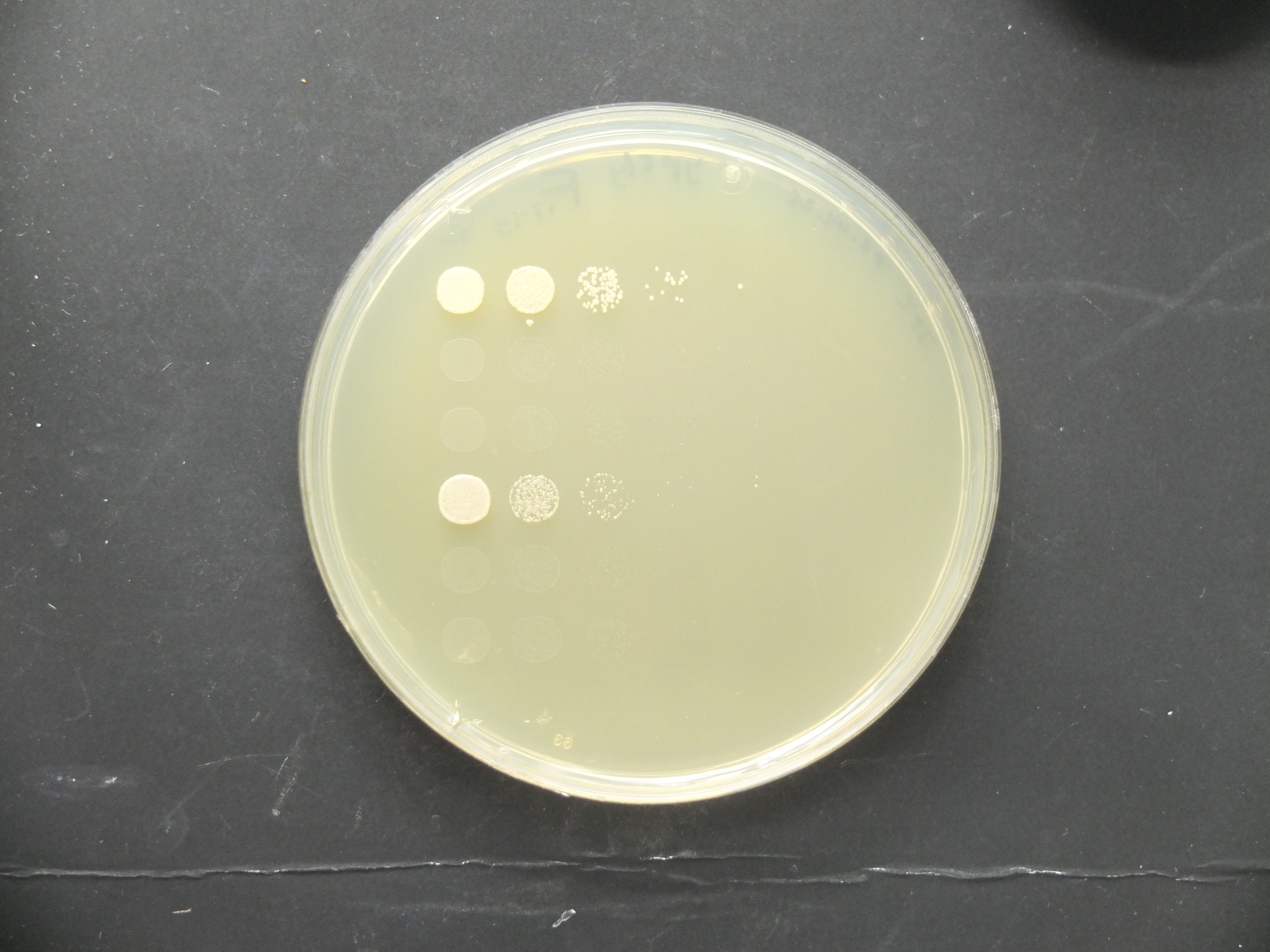

Supplement: Supplementary file 4 — Source data Fig. 2 [file 44318_2025_459_MOESM4_ESM.zip › Fig2/E/bottom/DSCF8403.JPG]

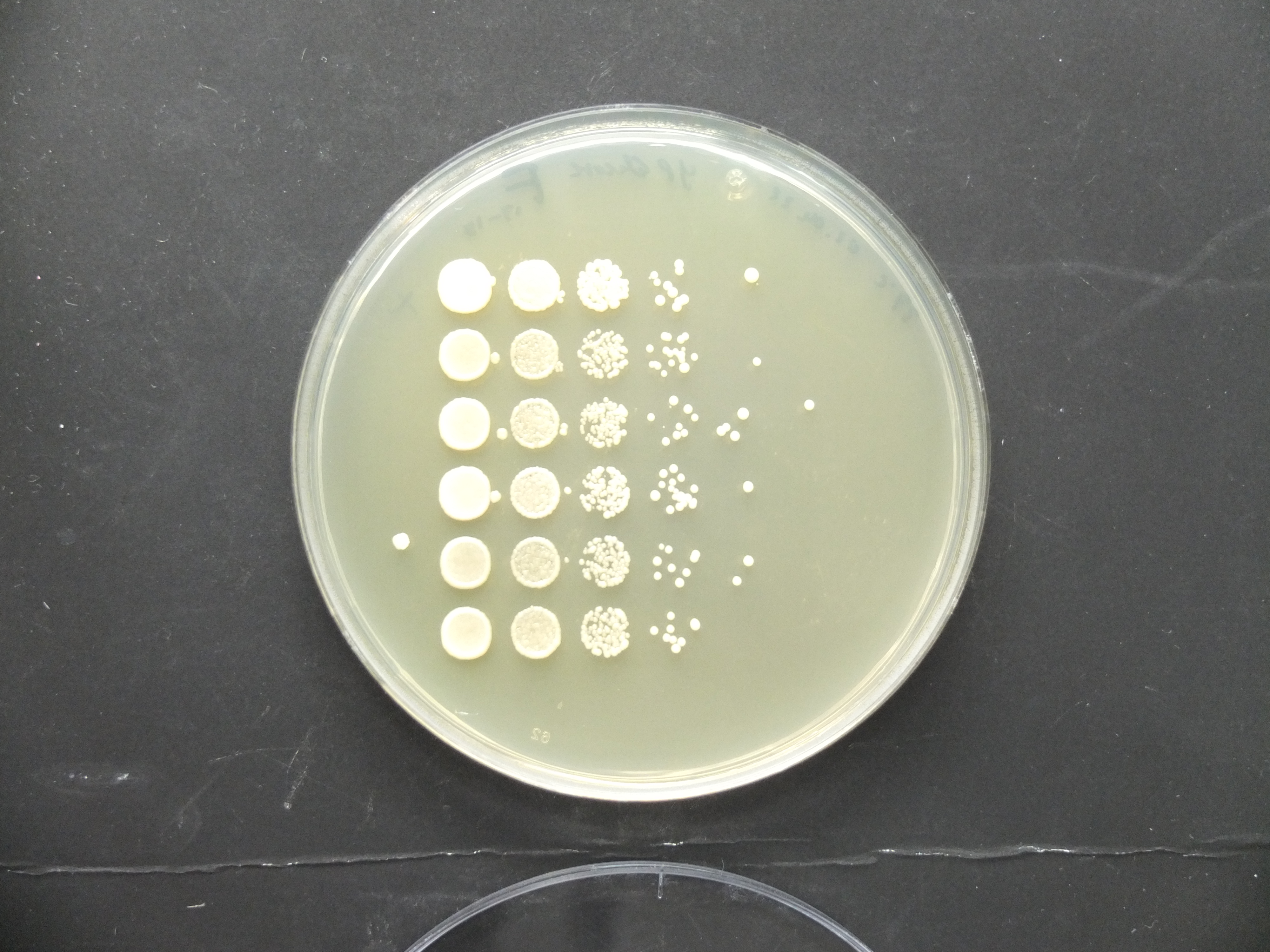

Supplement: Supplementary file 4 — Source data Fig. 2 [file 44318_2025_459_MOESM4_ESM.zip › Fig2/E/bottom/DSCF8365.JPG]

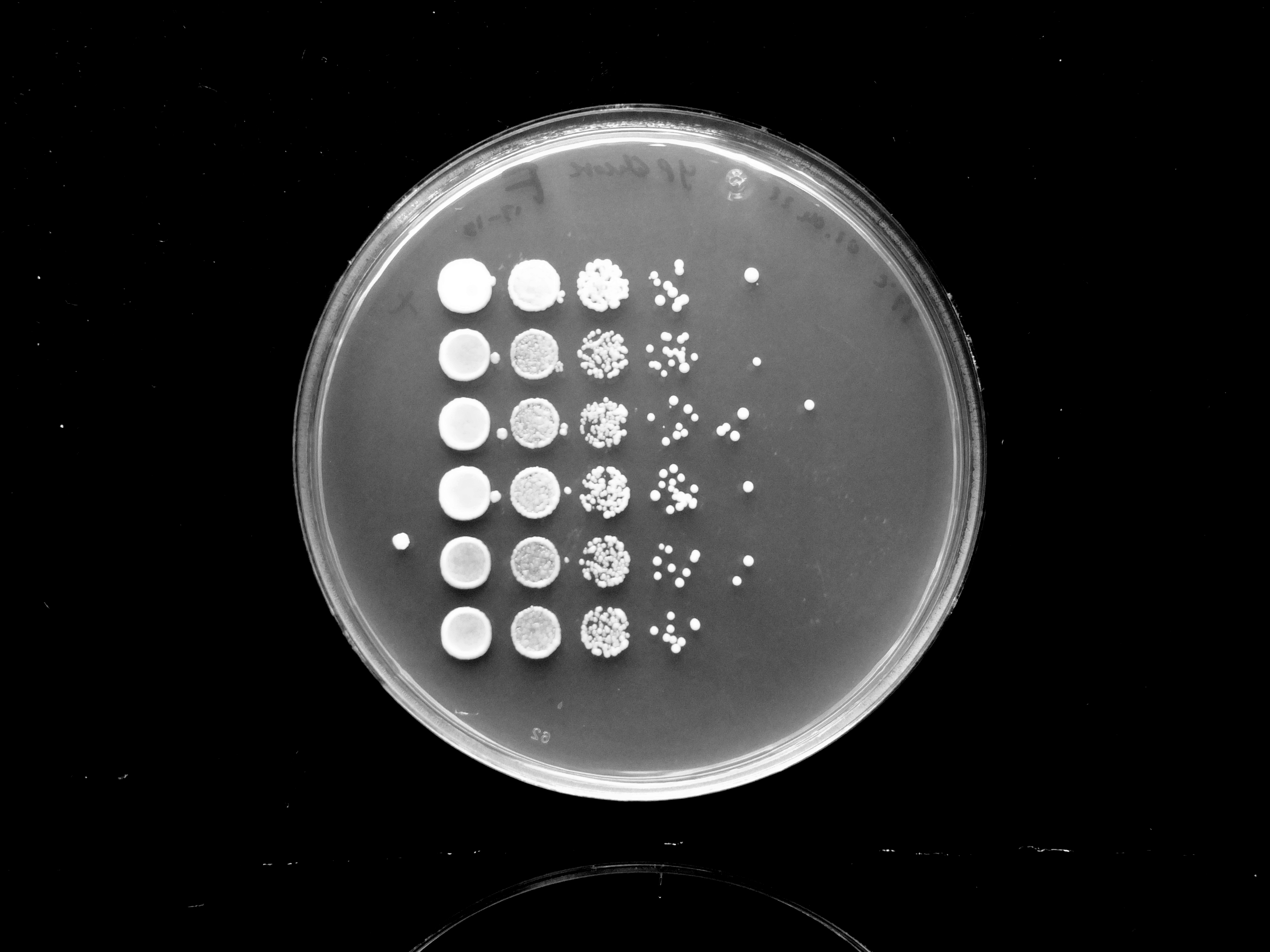

Supplement: Supplementary file 4 — Source data Fig. 2 [file 44318_2025_459_MOESM4_ESM.zip › Fig2/E/bottom/DSCF8365.tif]

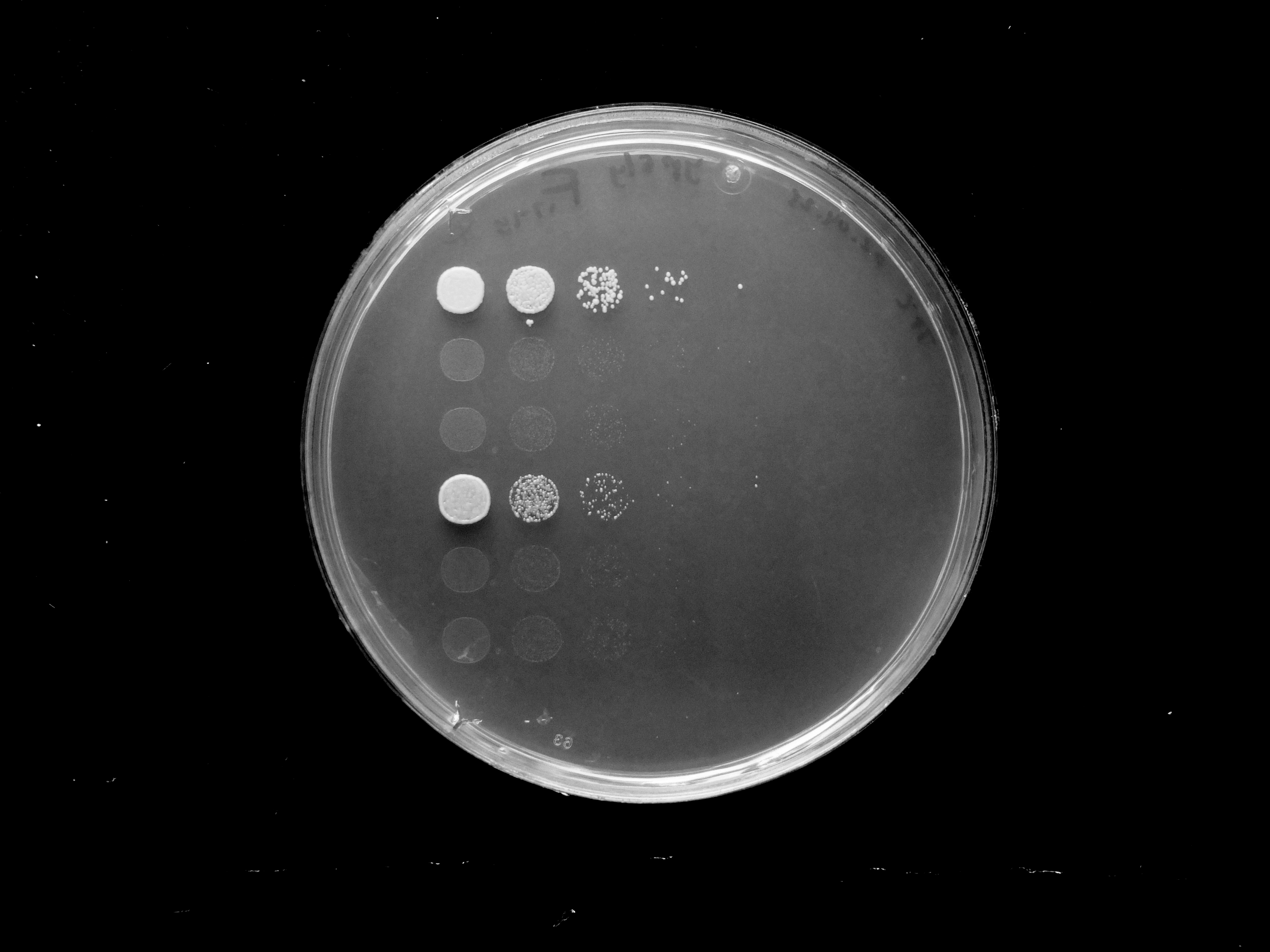

Supplement: Supplementary file 4 — Source data Fig. 2 [file 44318_2025_459_MOESM4_ESM.zip › Fig2/E/bottom/DSCF8403.tif]

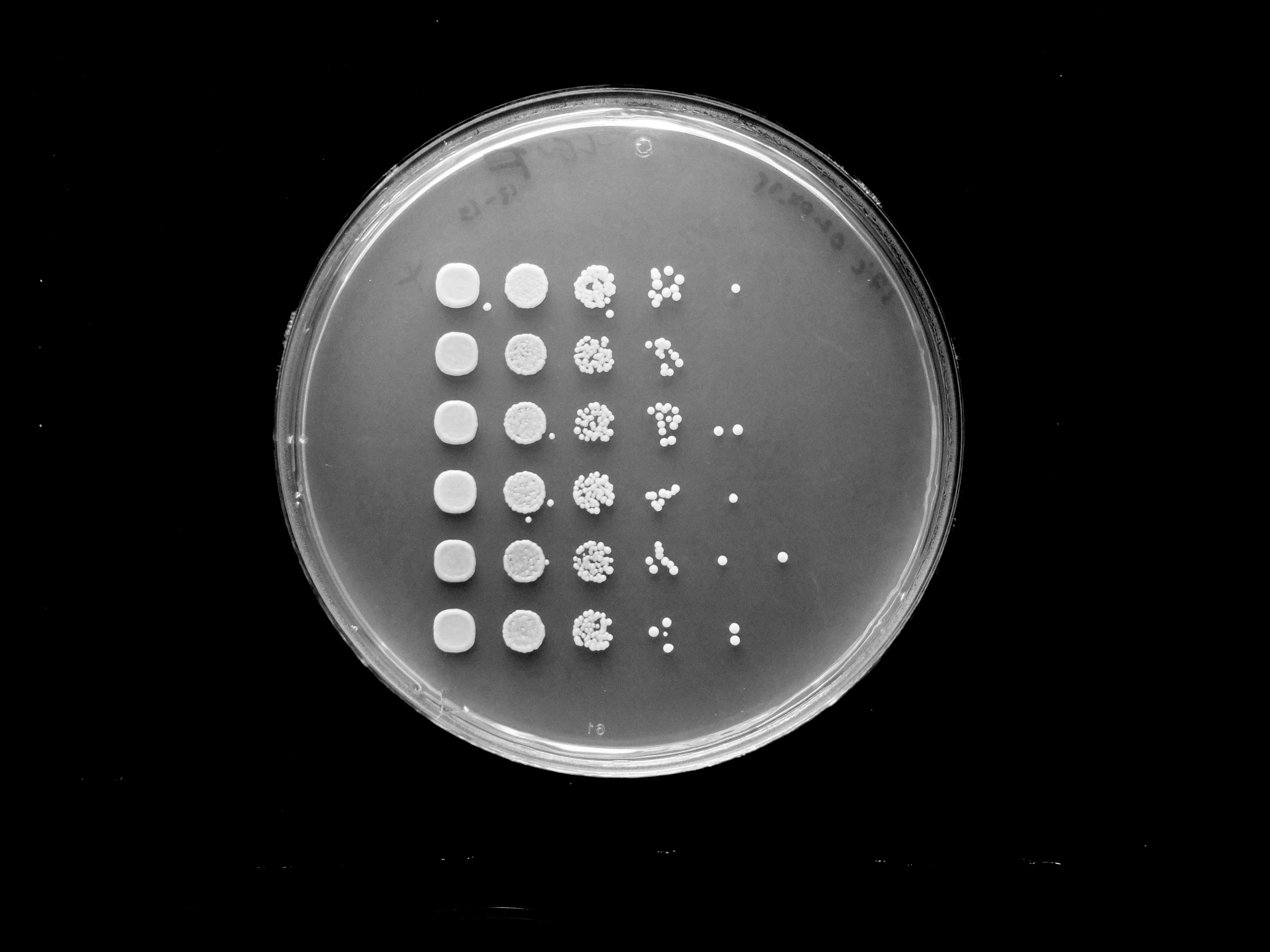

Supplement: Supplementary file 4 — Source data Fig. 2 [file 44318_2025_459_MOESM4_ESM.zip › Fig2/E/bottom/DSCF8377.tif]

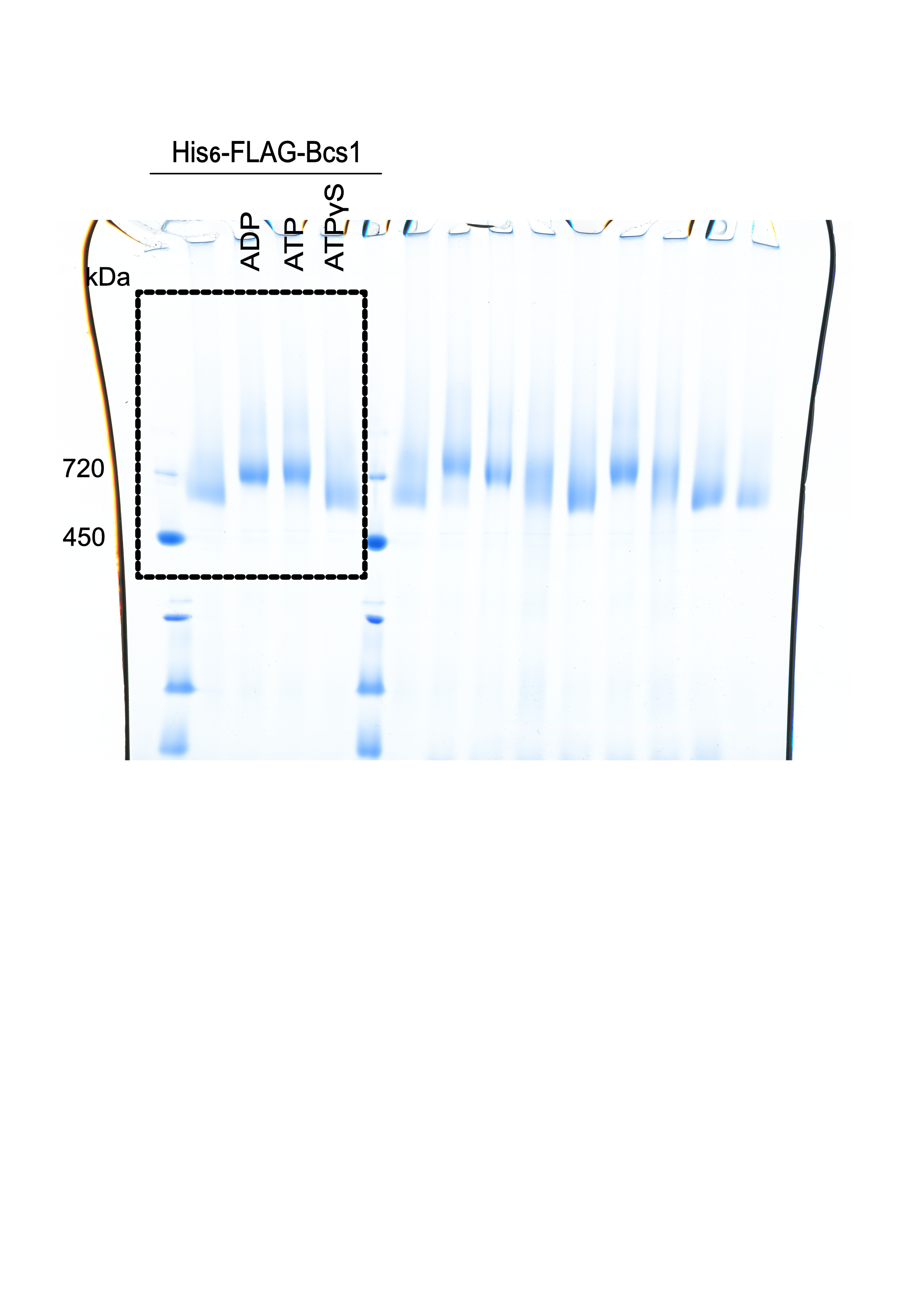

Supplement: Supplementary file 5 — Source data Fig. 3 [file 44318_2025_459_MOESM5_ESM.zip › Fig3/A/Fig3_source.png]

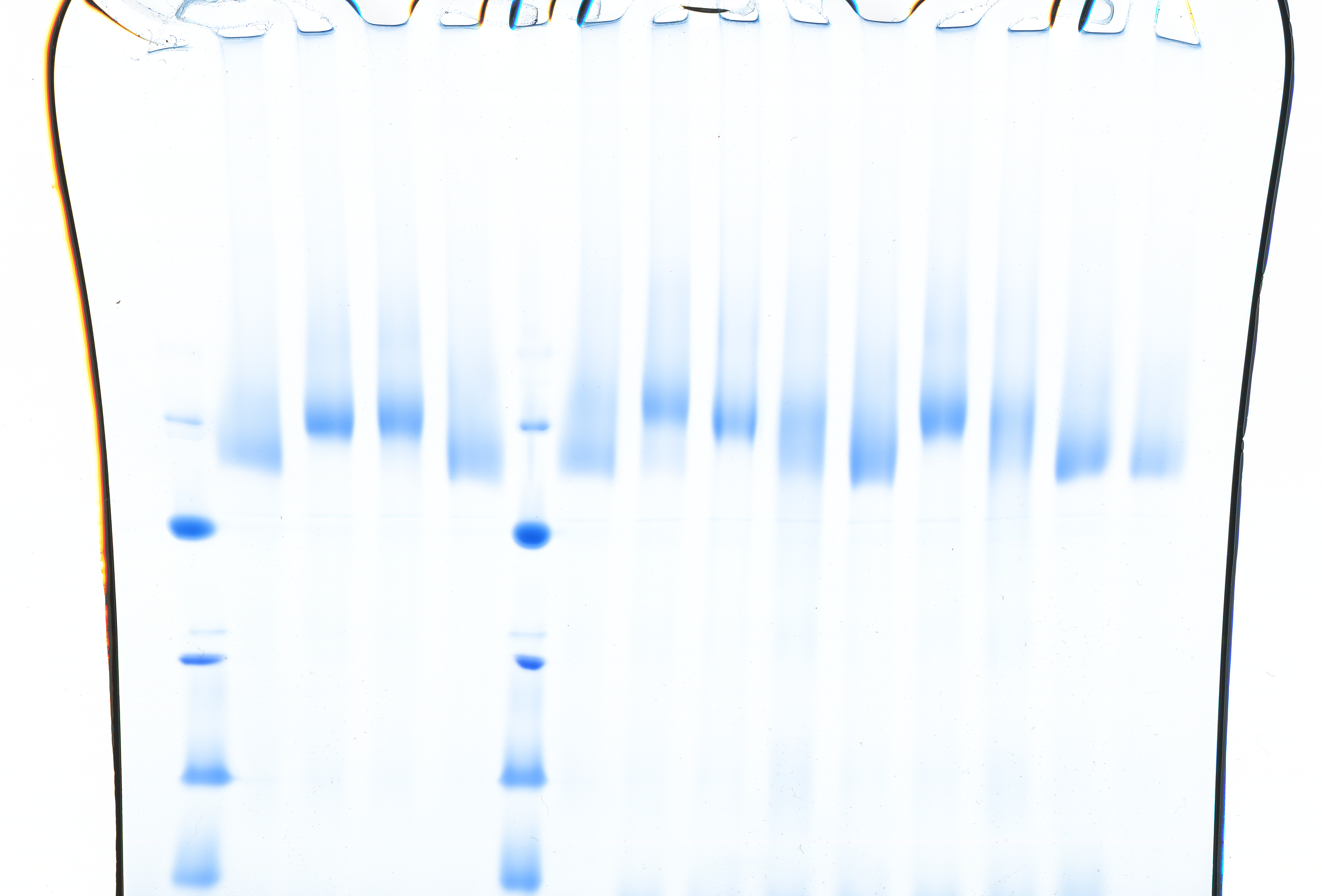

Supplement: Supplementary file 5 — Source data Fig. 3 [file 44318_2025_459_MOESM5_ESM.zip › Fig3/A/240907 Bcs1BN012.tif]

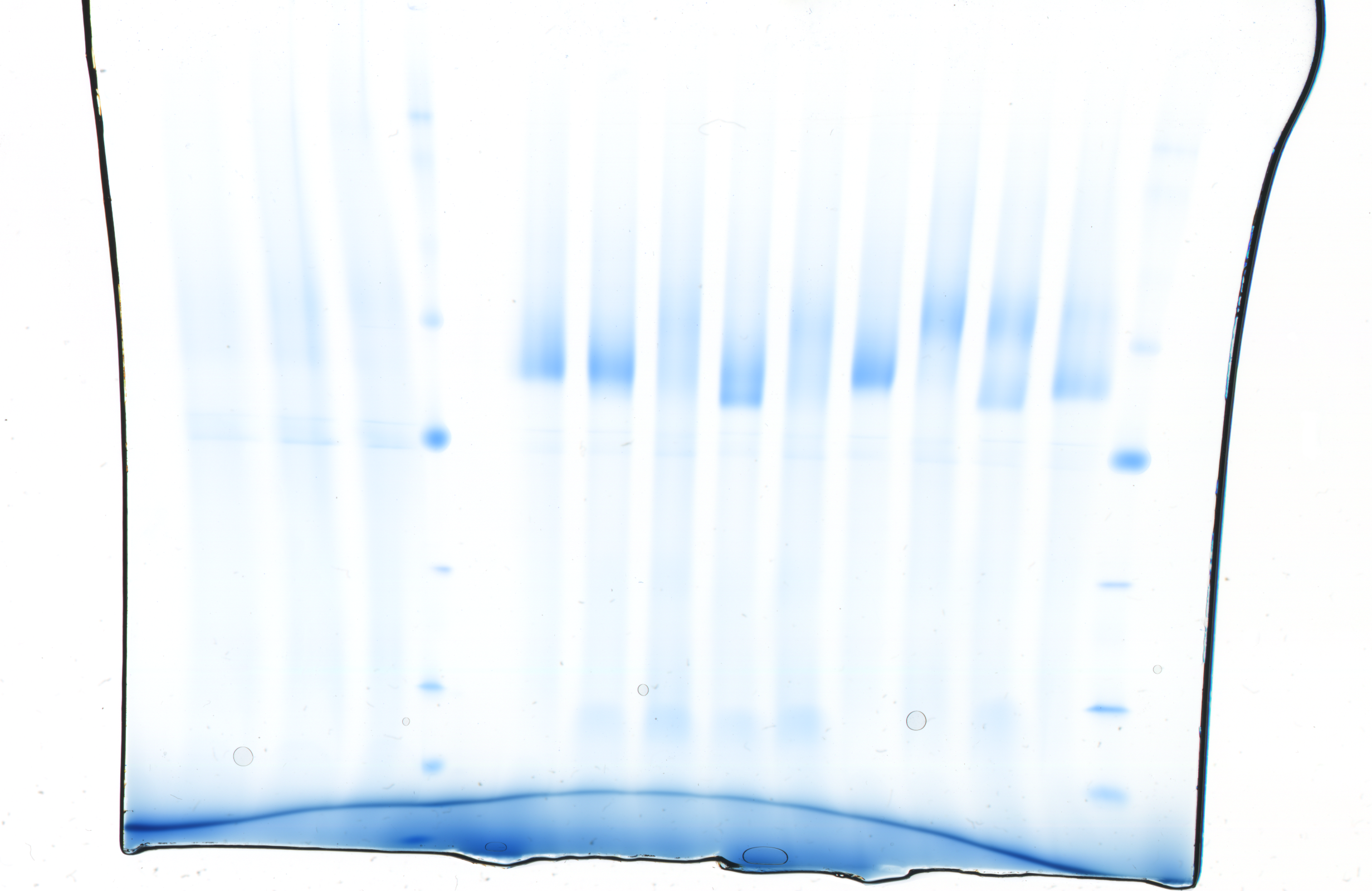

Supplement: Supplementary file 6 — Source data Fig. 4 [file 44318_2025_459_MOESM6_ESM.zip › Fig4/A/231124 BNBcs1001.tif]

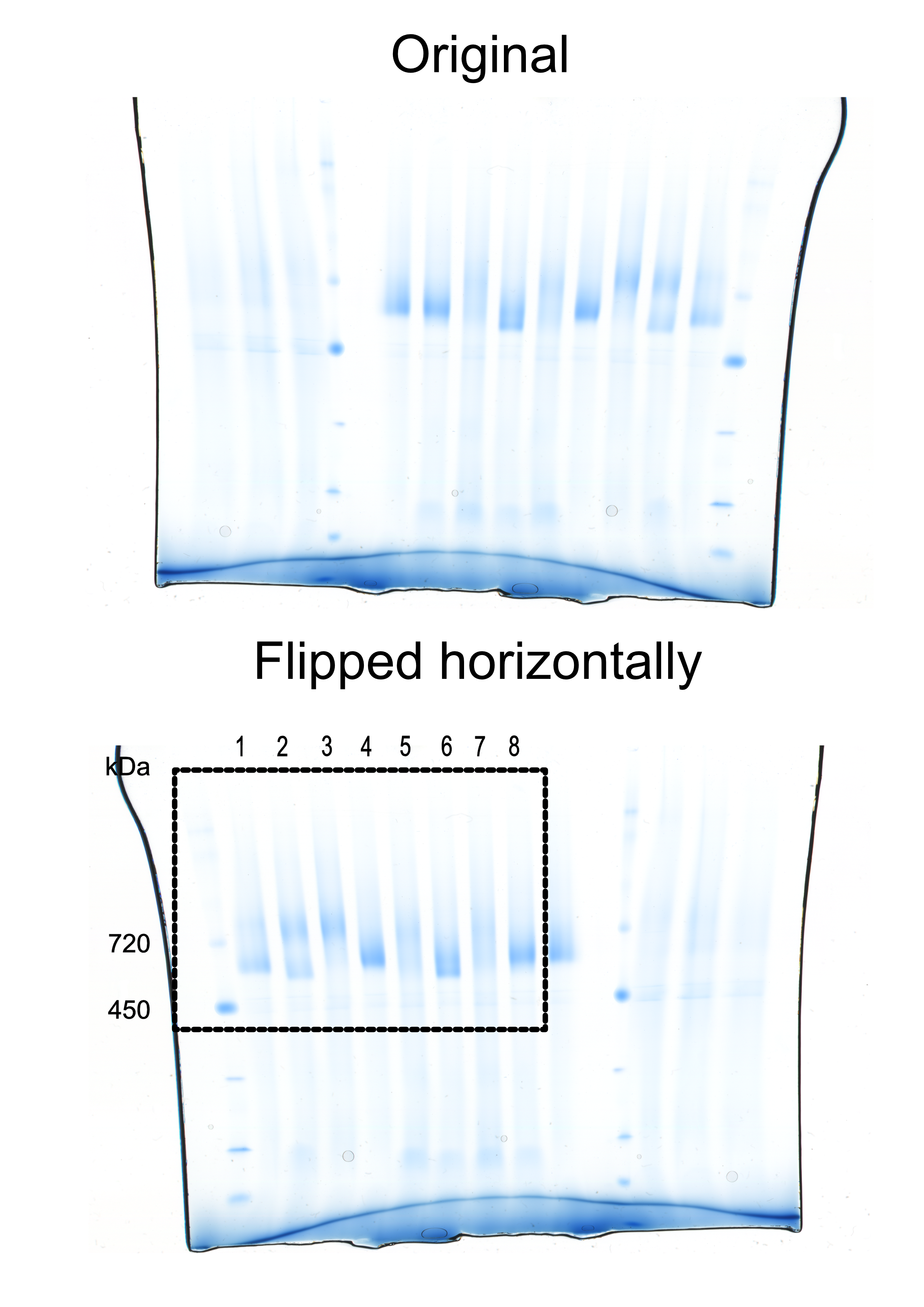

Supplement: Supplementary file 6 — Source data Fig. 4 [file 44318_2025_459_MOESM6_ESM.zip › Fig4/A/Fig4_source.png]

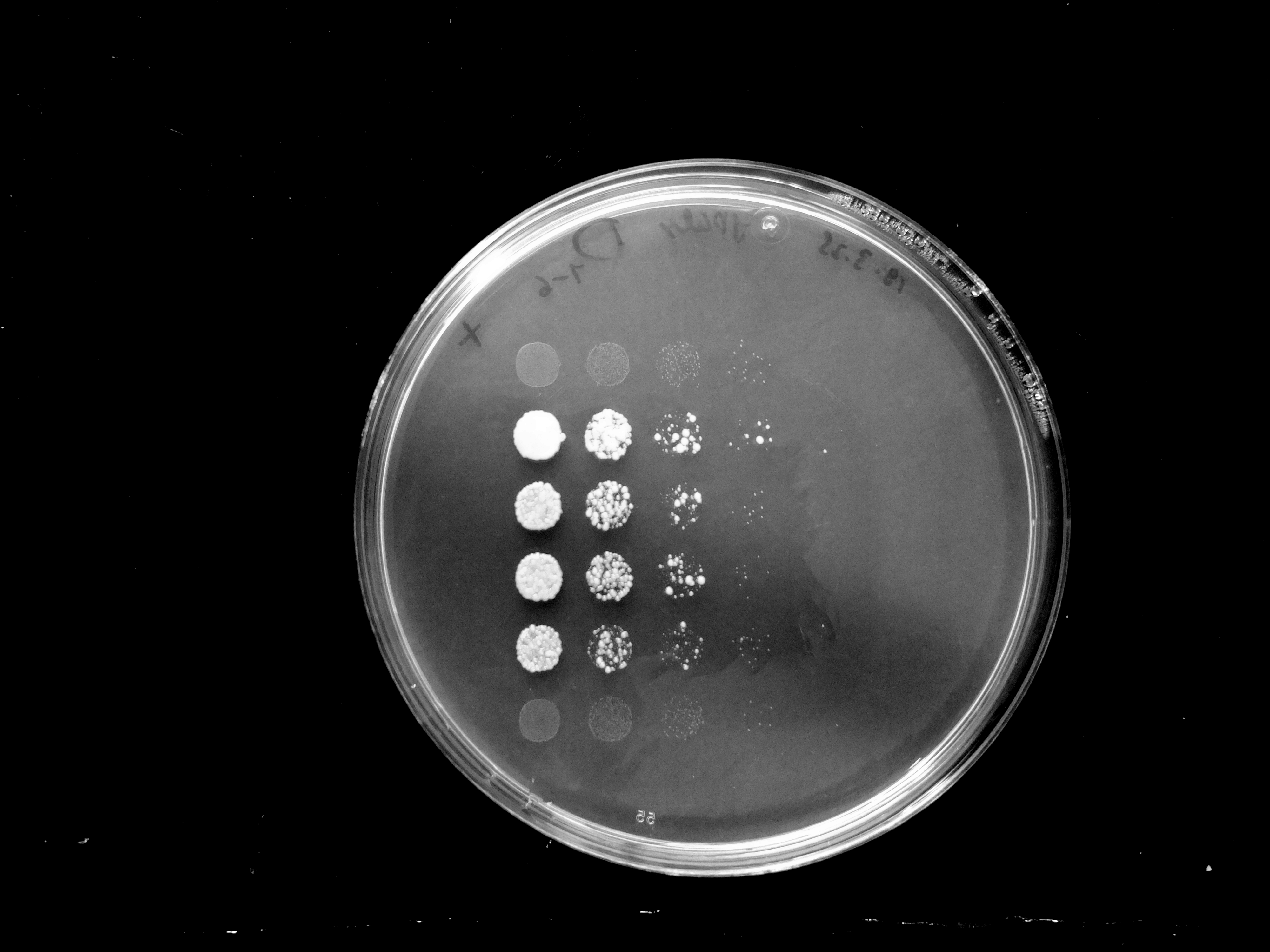

Supplement: Supplementary file 6 — Source data Fig. 4 [file 44318_2025_459_MOESM6_ESM.zip › Fig4/E/DSCF8316-2.tif]

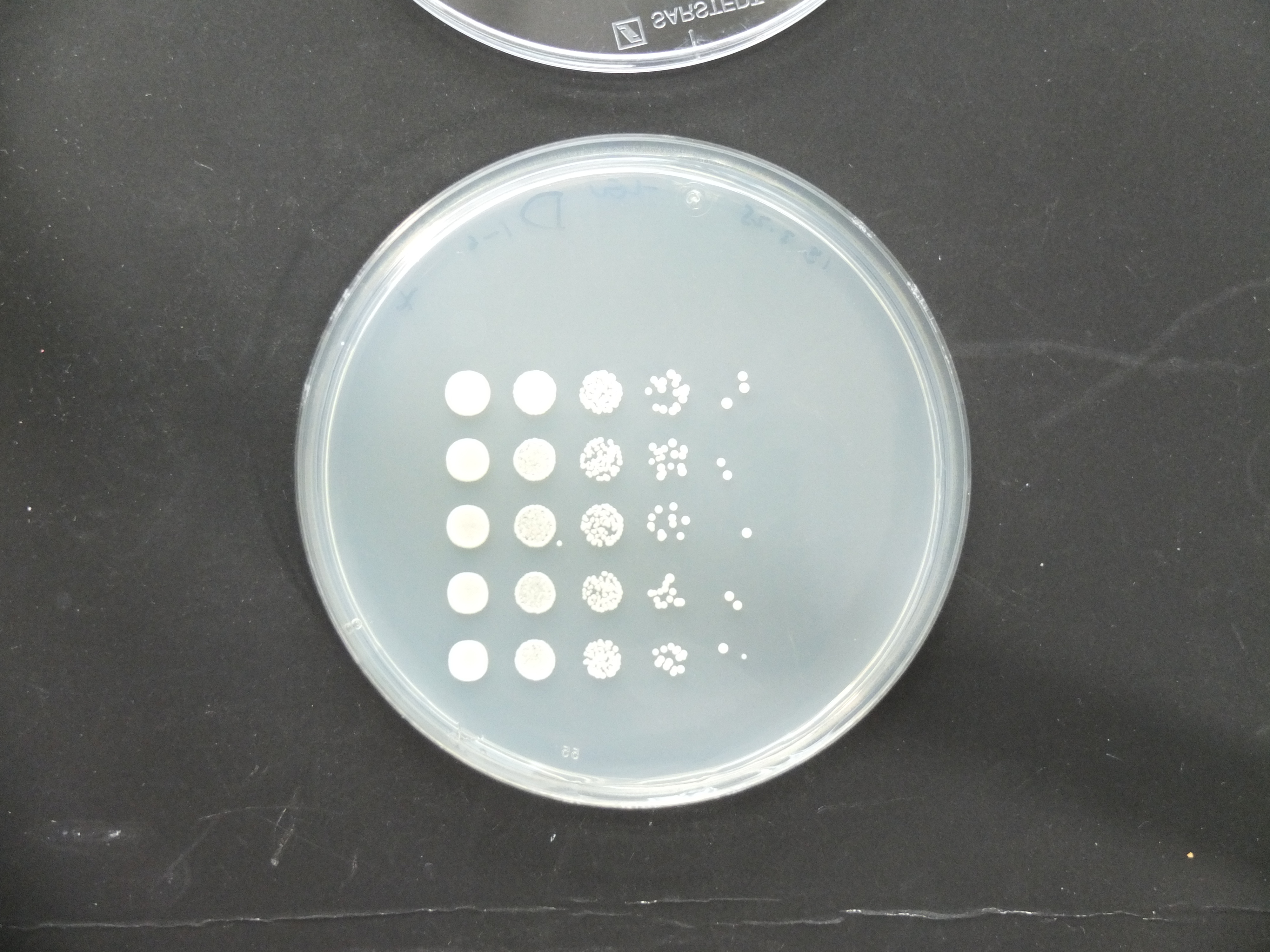

Supplement: Supplementary file 6 — Source data Fig. 4 [file 44318_2025_459_MOESM6_ESM.zip › Fig4/E/DSCF8287.JPG]

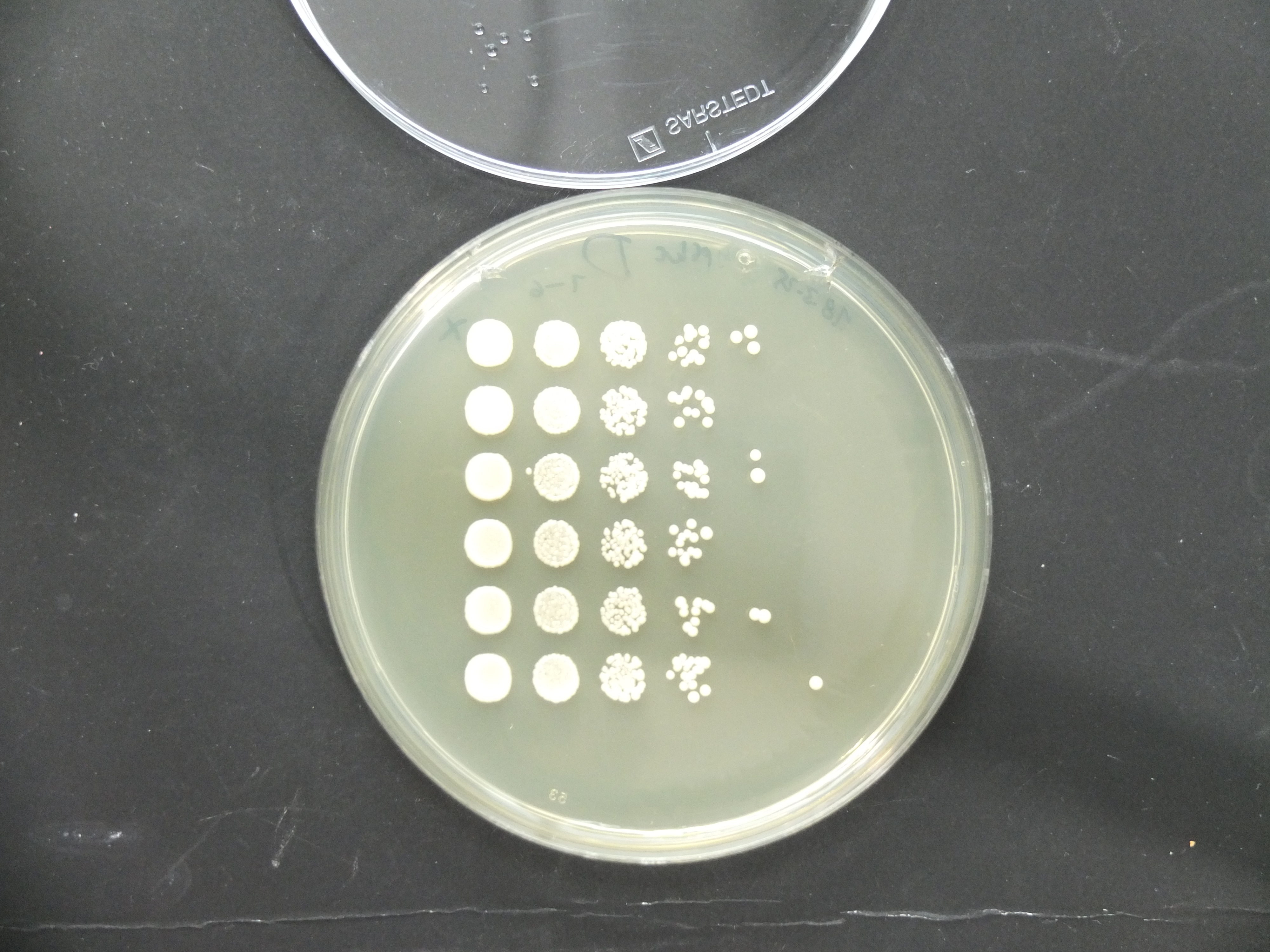

Supplement: Supplementary file 6 — Source data Fig. 4 [file 44318_2025_459_MOESM6_ESM.zip › Fig4/E/DSCF8279.JPG]

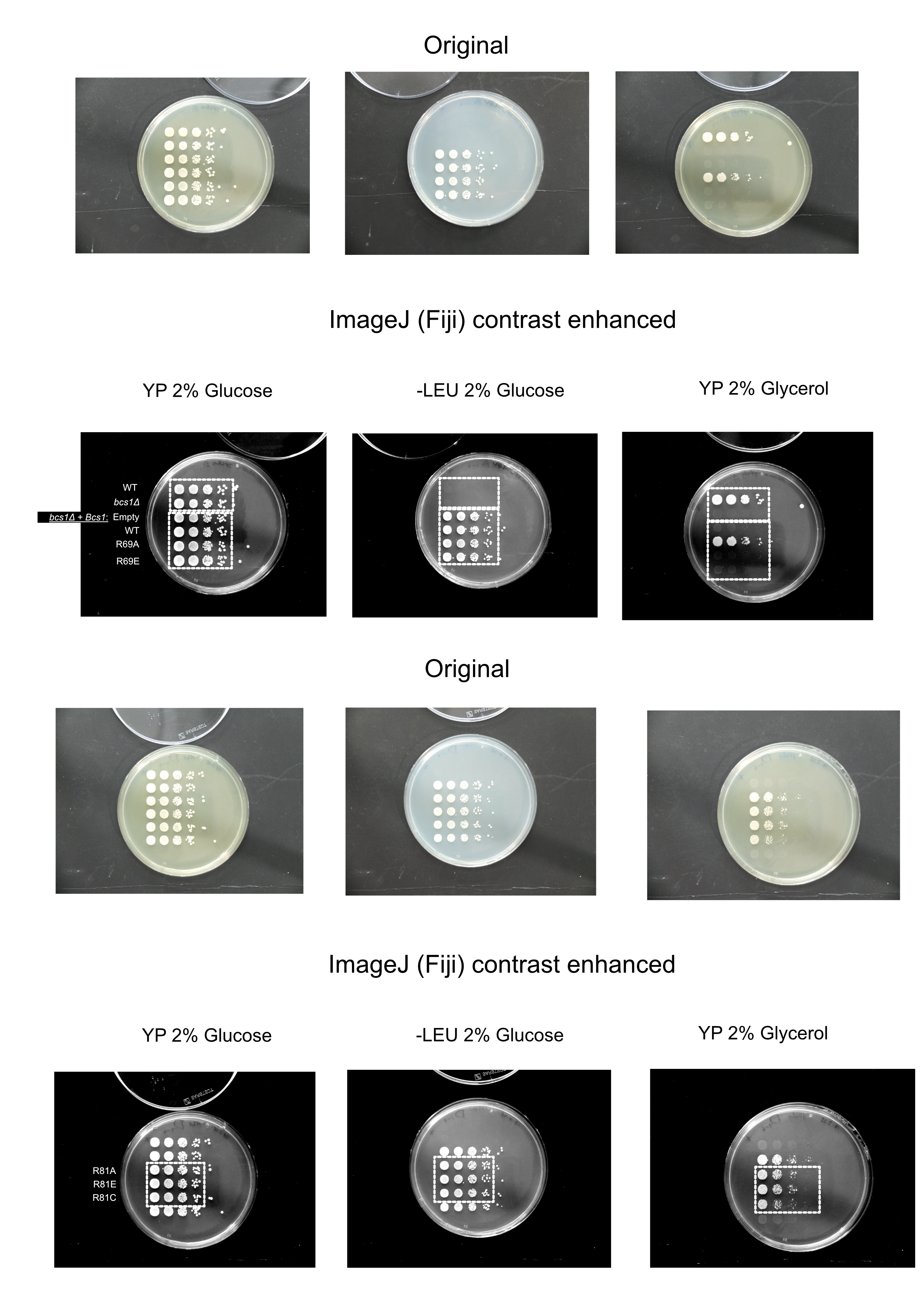

Supplement: Supplementary file 6 — Source data Fig. 4 [file 44318_2025_459_MOESM6_ESM.zip › Fig4/E/Fig4E_source.png]

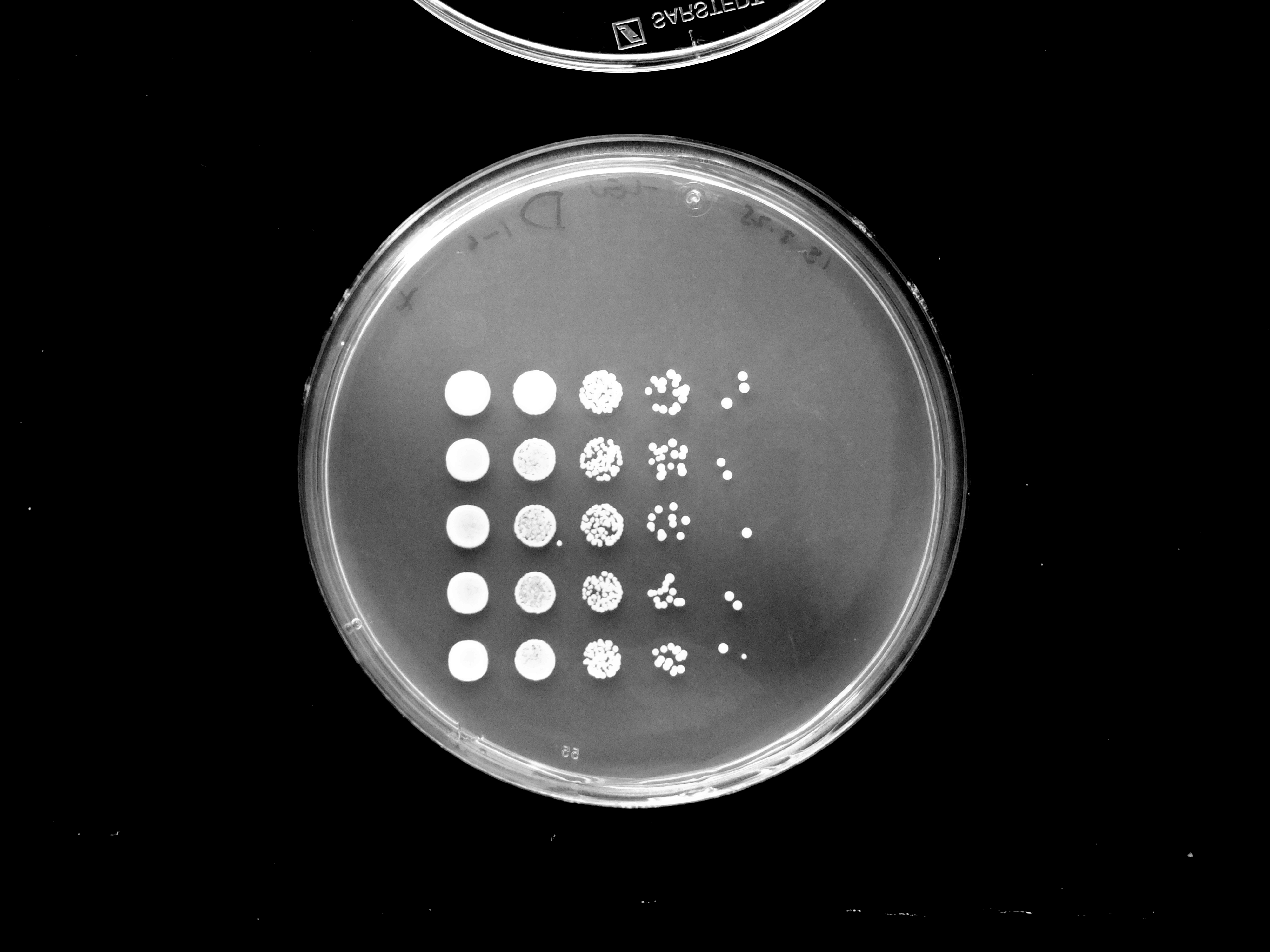

Supplement: Supplementary file 6 — Source data Fig. 4 [file 44318_2025_459_MOESM6_ESM.zip › Fig4/E/DSCF8287-2.tif]

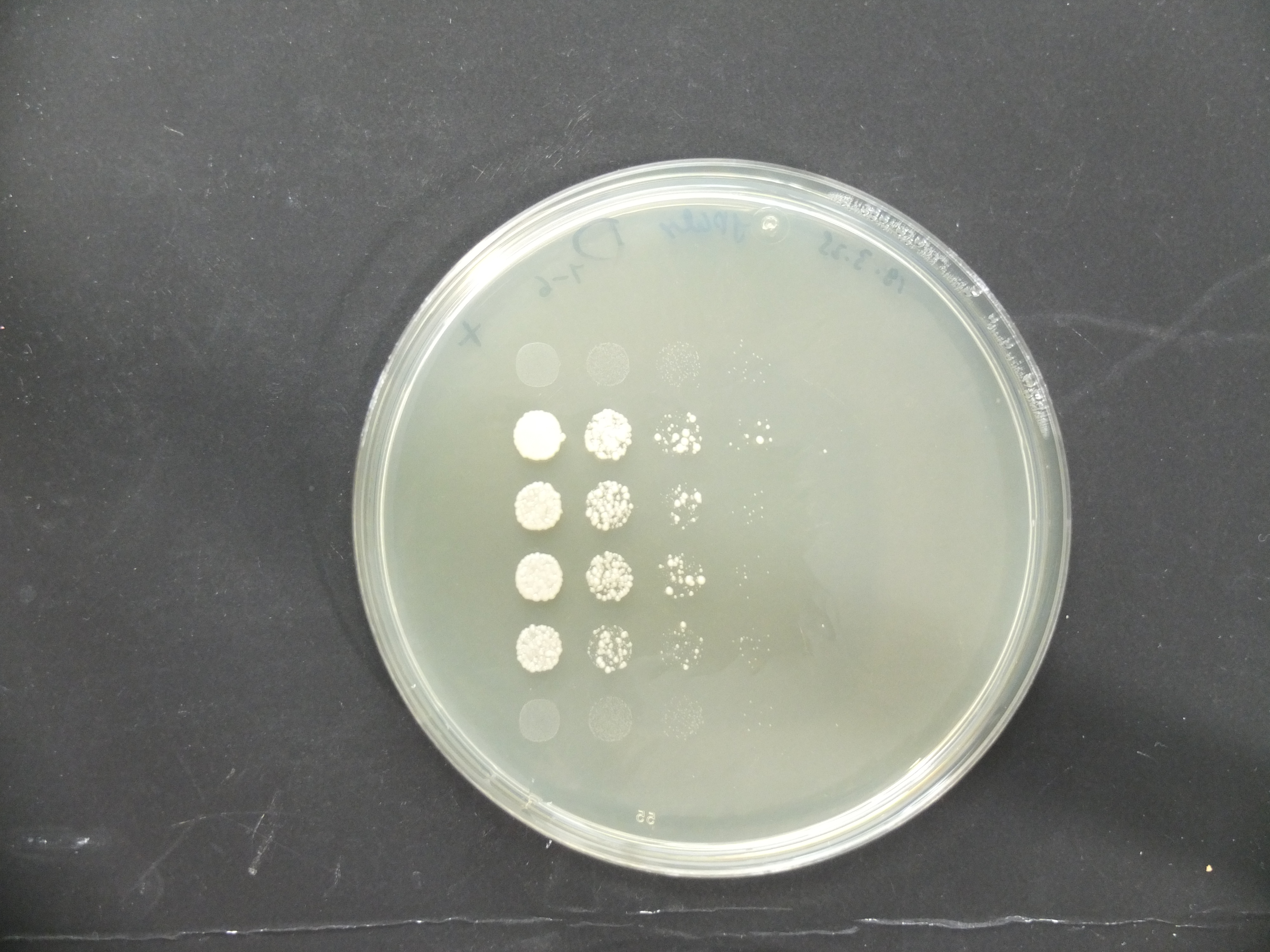

Supplement: Supplementary file 6 — Source data Fig. 4 [file 44318_2025_459_MOESM6_ESM.zip › Fig4/E/DSCF8316.JPG]

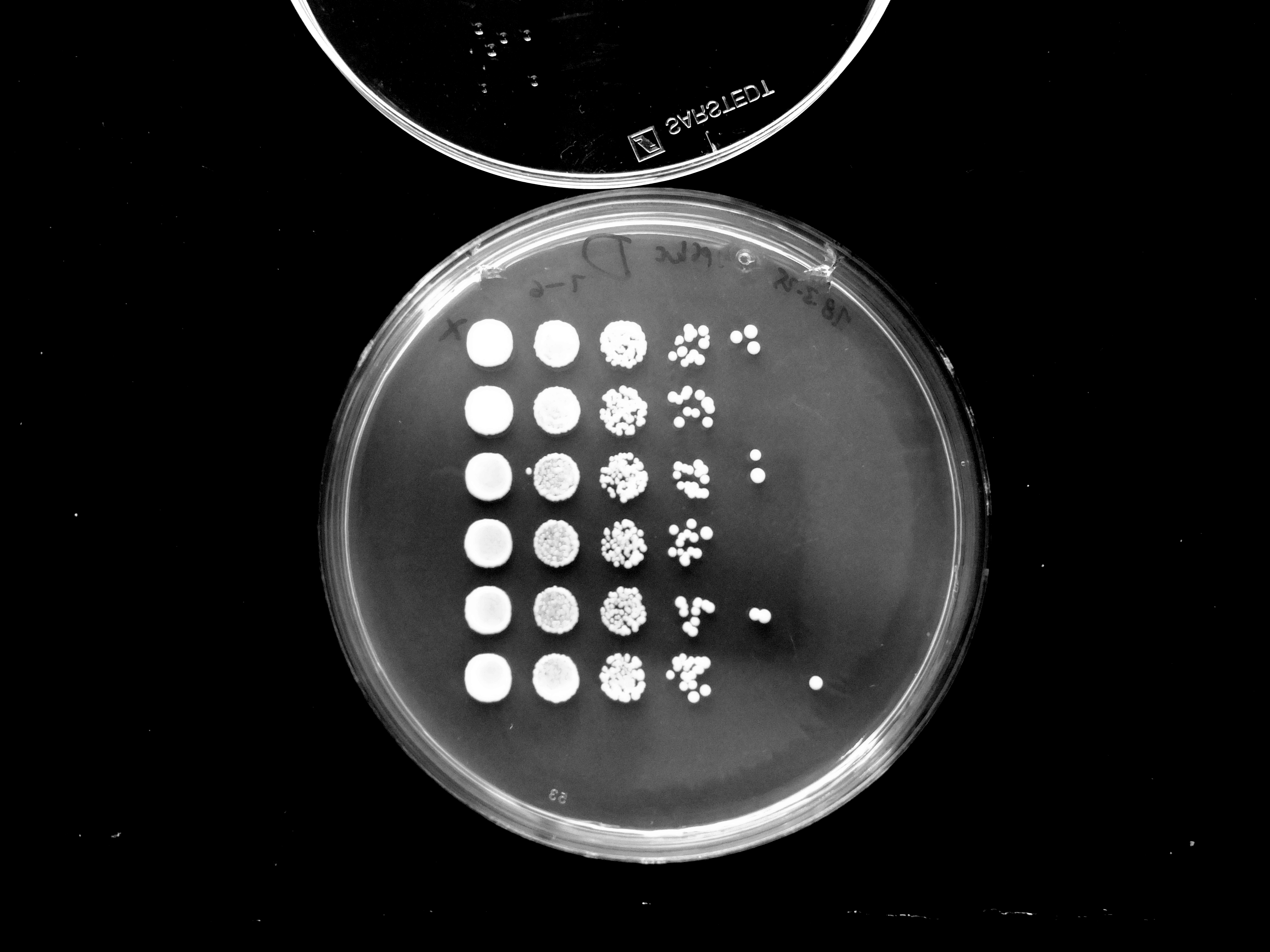

Supplement: Supplementary file 6 — Source data Fig. 4 [file 44318_2025_459_MOESM6_ESM.zip › Fig4/E/DSCF8279-2.tif]

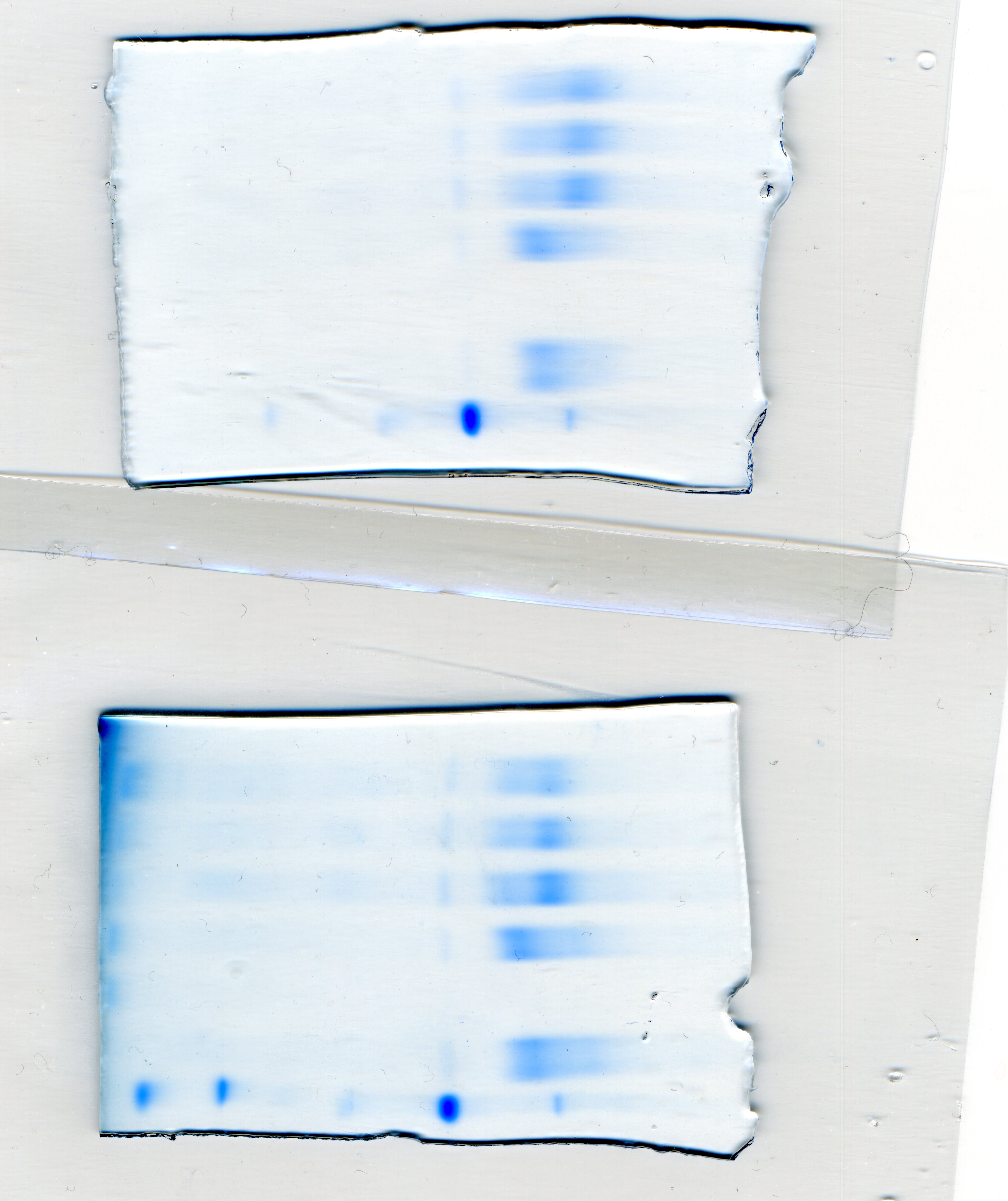

Supplement: Supplementary file 7 — Figure EV3 Source Data [file 44318_2025_459_MOESM7_ESM.zip › EV3/B/left panel/221216 BN strep82026.jpg]

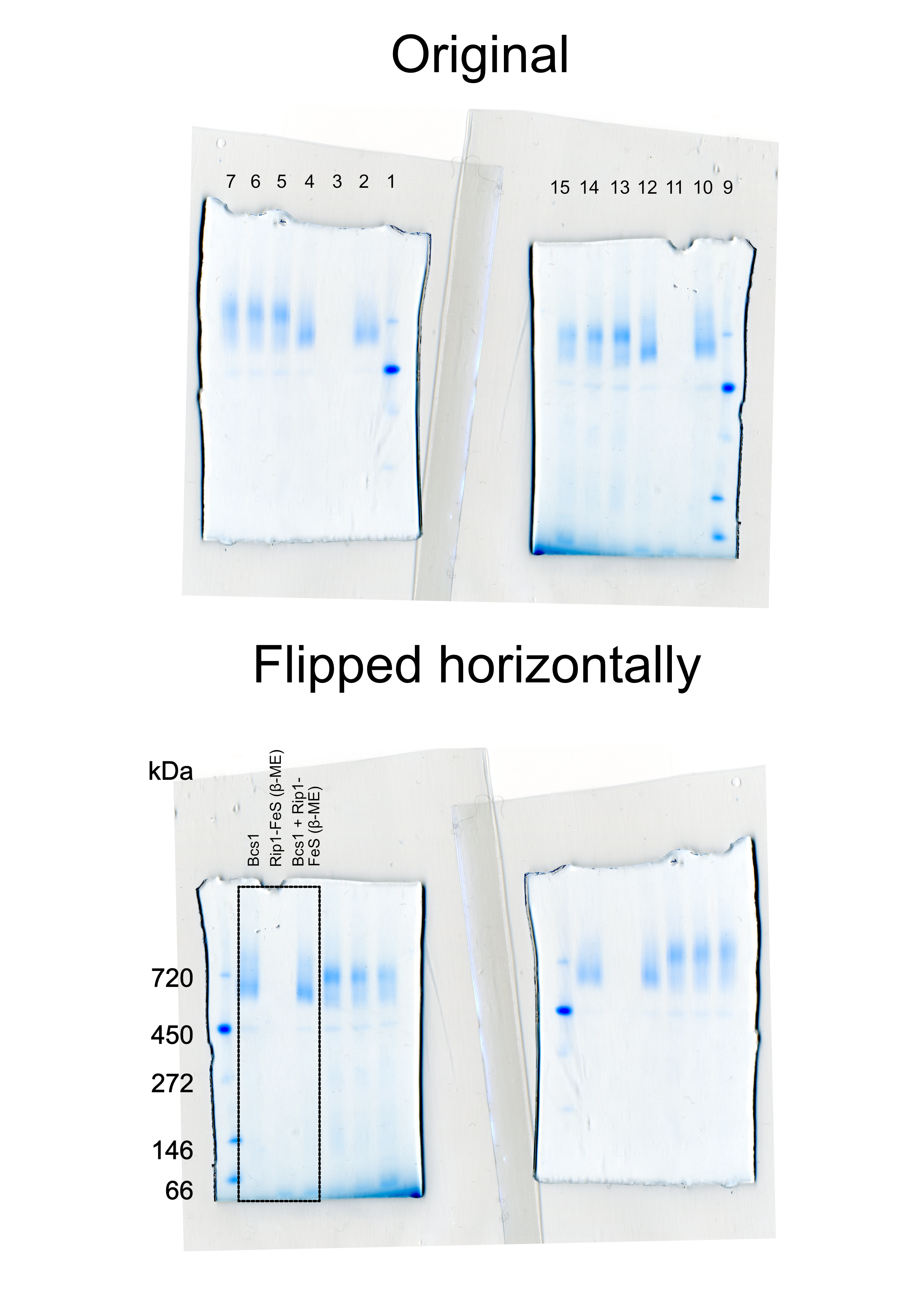

Supplement: Supplementary file 7 — Figure EV3 Source Data [file 44318_2025_459_MOESM7_ESM.zip › EV3/B/left panel/EV3B_left_source.png]

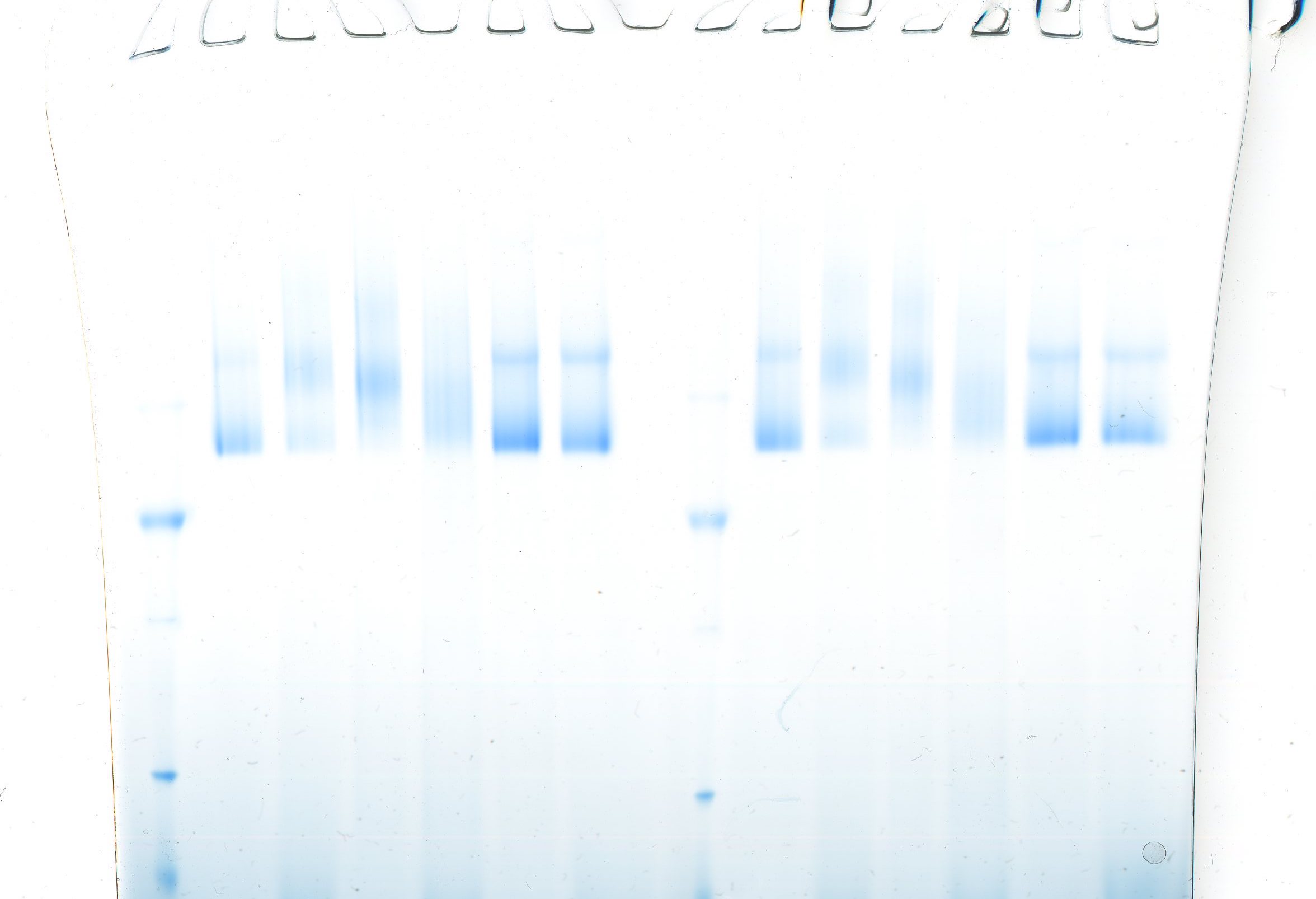

Supplement: Supplementary file 7 — Figure EV3 Source Data [file 44318_2025_459_MOESM7_ESM.zip › EV3/B/right panel/230711230711 BNcheckbcs1005.jpg]

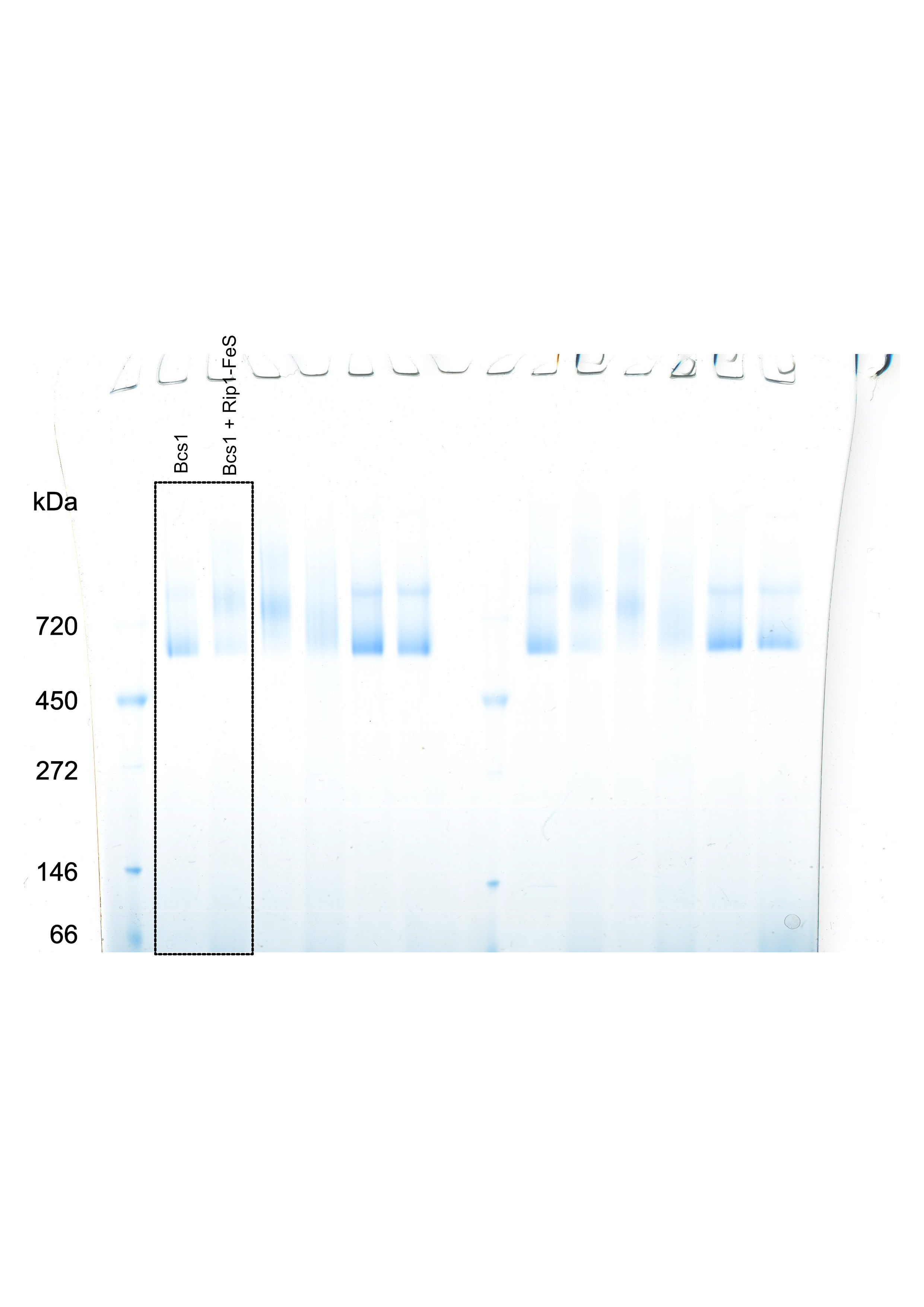

Supplement: Supplementary file 7 — Figure EV3 Source Data [file 44318_2025_459_MOESM7_ESM.zip › EV3/B/right panel/EV3B_right_source.png]

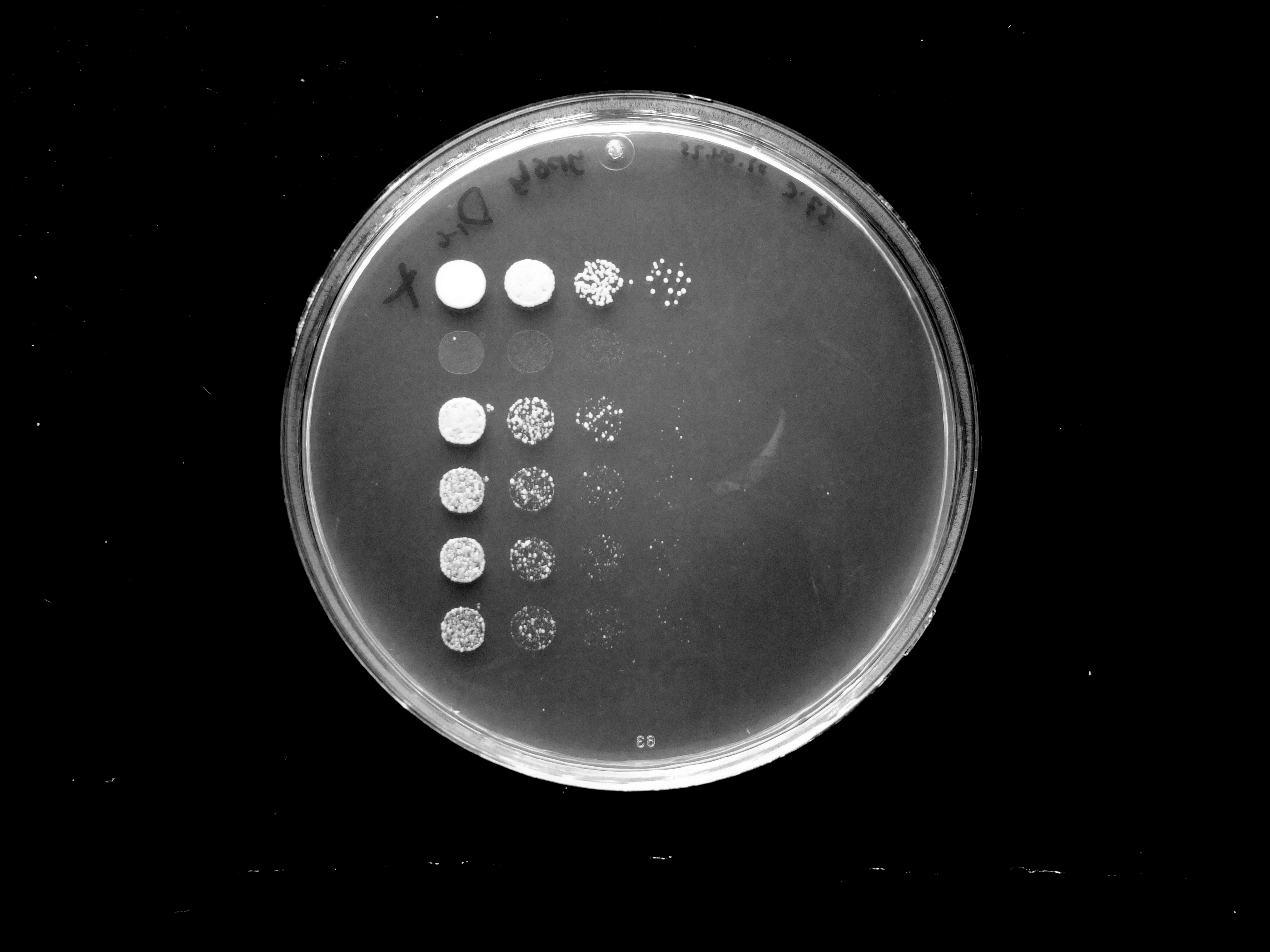

Supplement: Supplementary file 8 — Appendix Figure S6 Source Data [file 44318_2025_459_MOESM8_ESM.zip › Appendix Fig S6/A/right bottom/DSCF8395.tif]

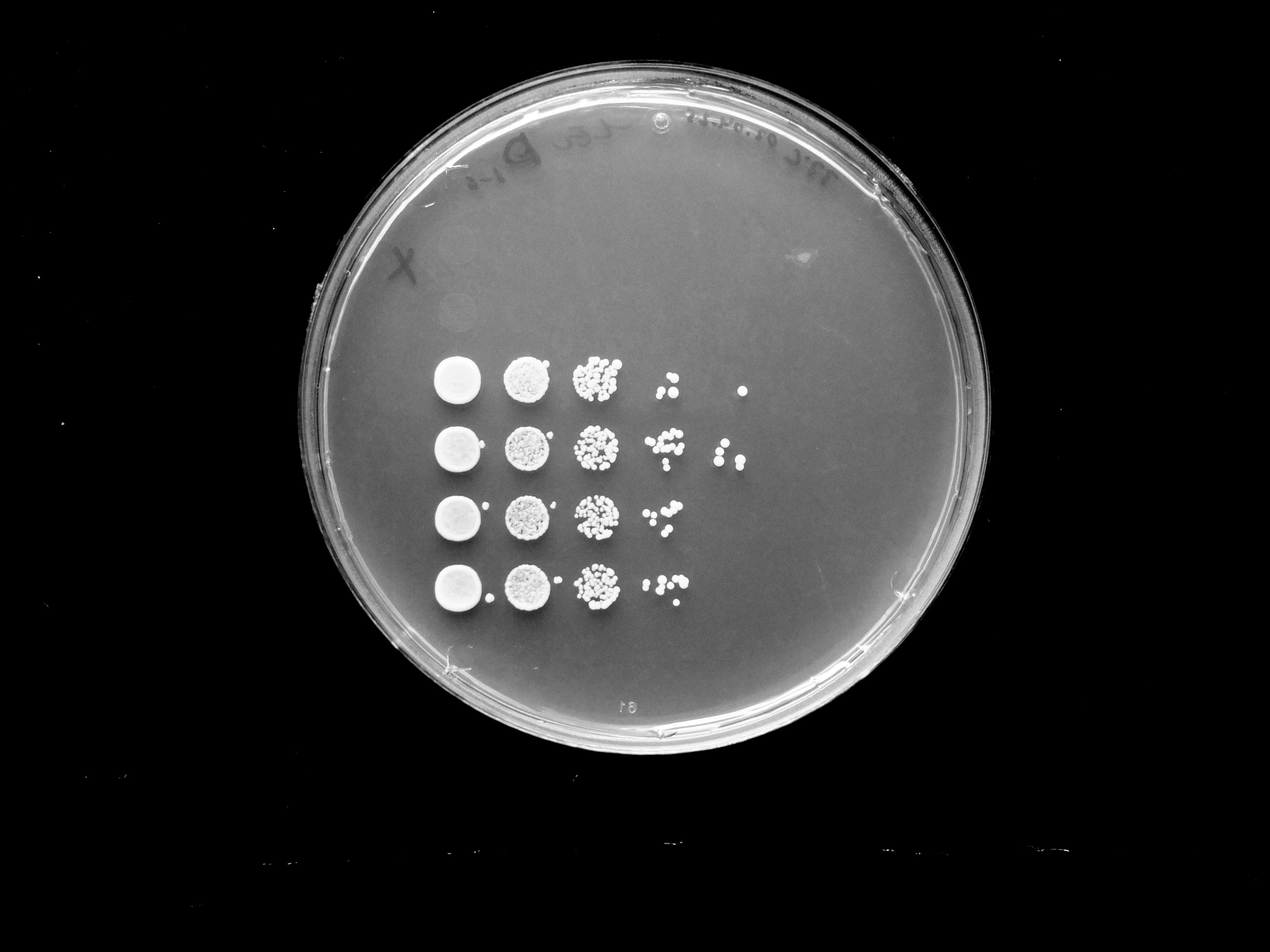

Supplement: Supplementary file 8 — Appendix Figure S6 Source Data [file 44318_2025_459_MOESM8_ESM.zip › Appendix Fig S6/A/right bottom/DSCF8369.tif]

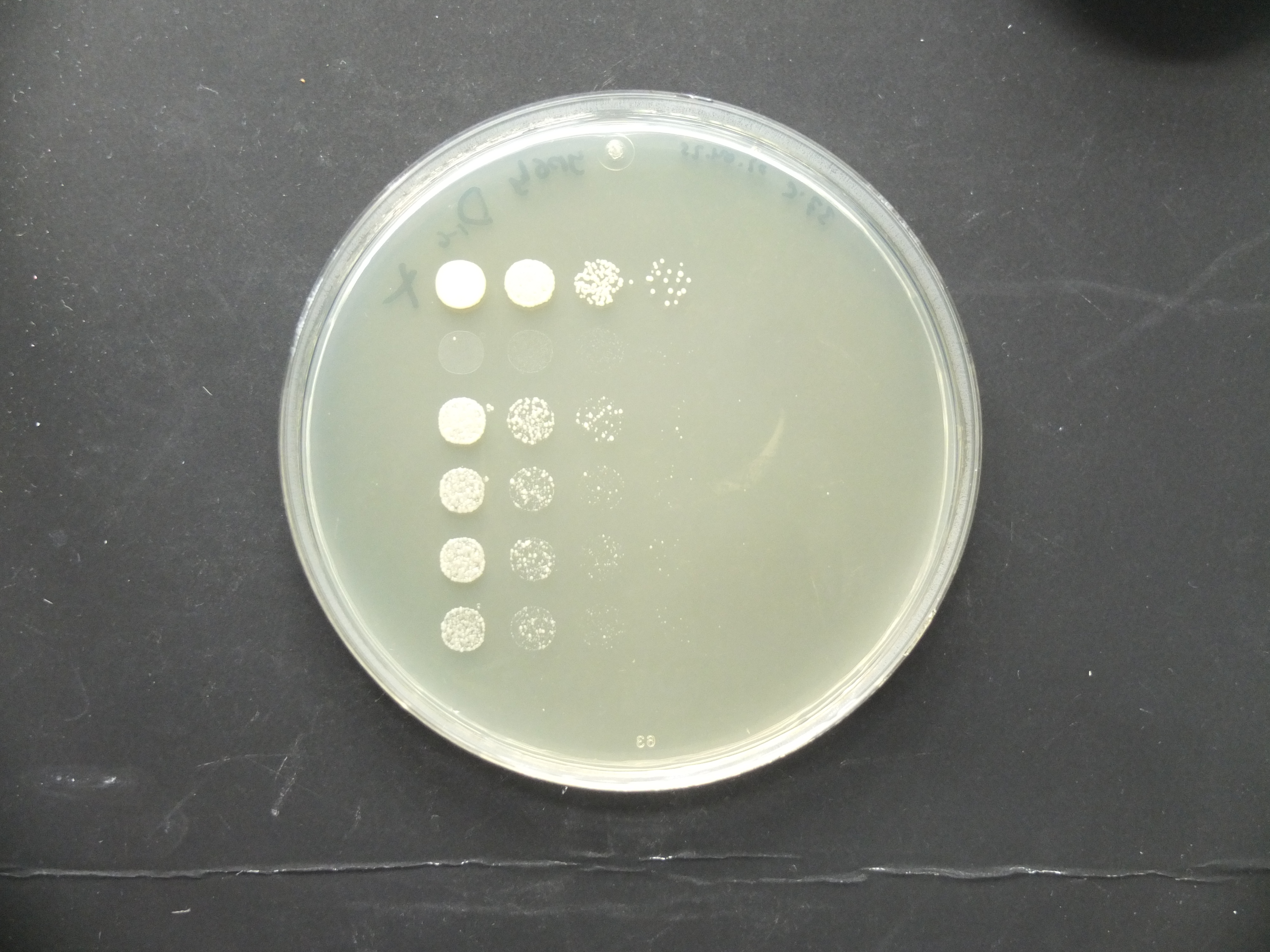

Supplement: Supplementary file 8 — Appendix Figure S6 Source Data [file 44318_2025_459_MOESM8_ESM.zip › Appendix Fig S6/A/right bottom/DSCF8395.JPG]

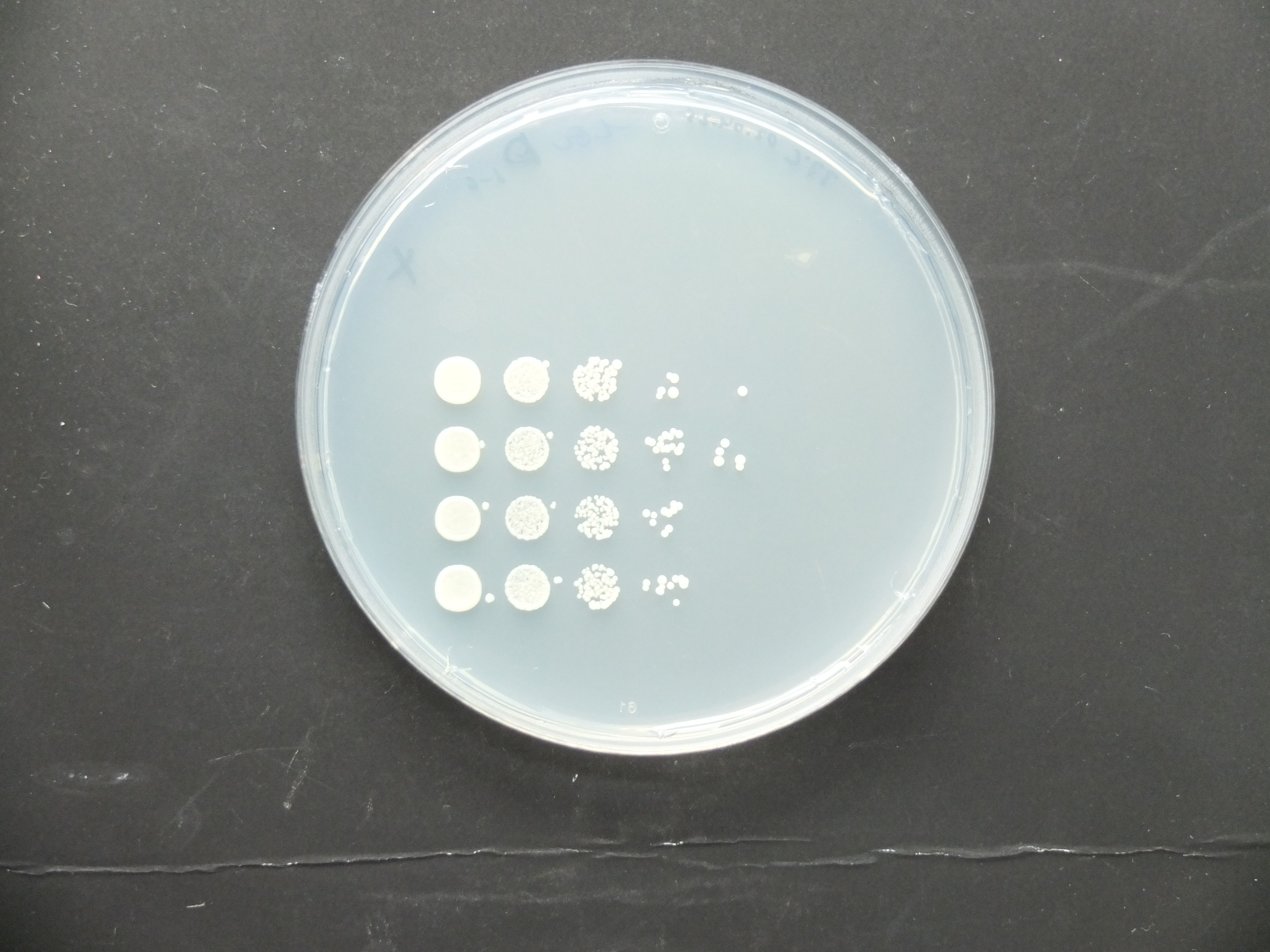

Supplement: Supplementary file 8 — Appendix Figure S6 Source Data [file 44318_2025_459_MOESM8_ESM.zip › Appendix Fig S6/A/right bottom/DSCF8369.JPG]

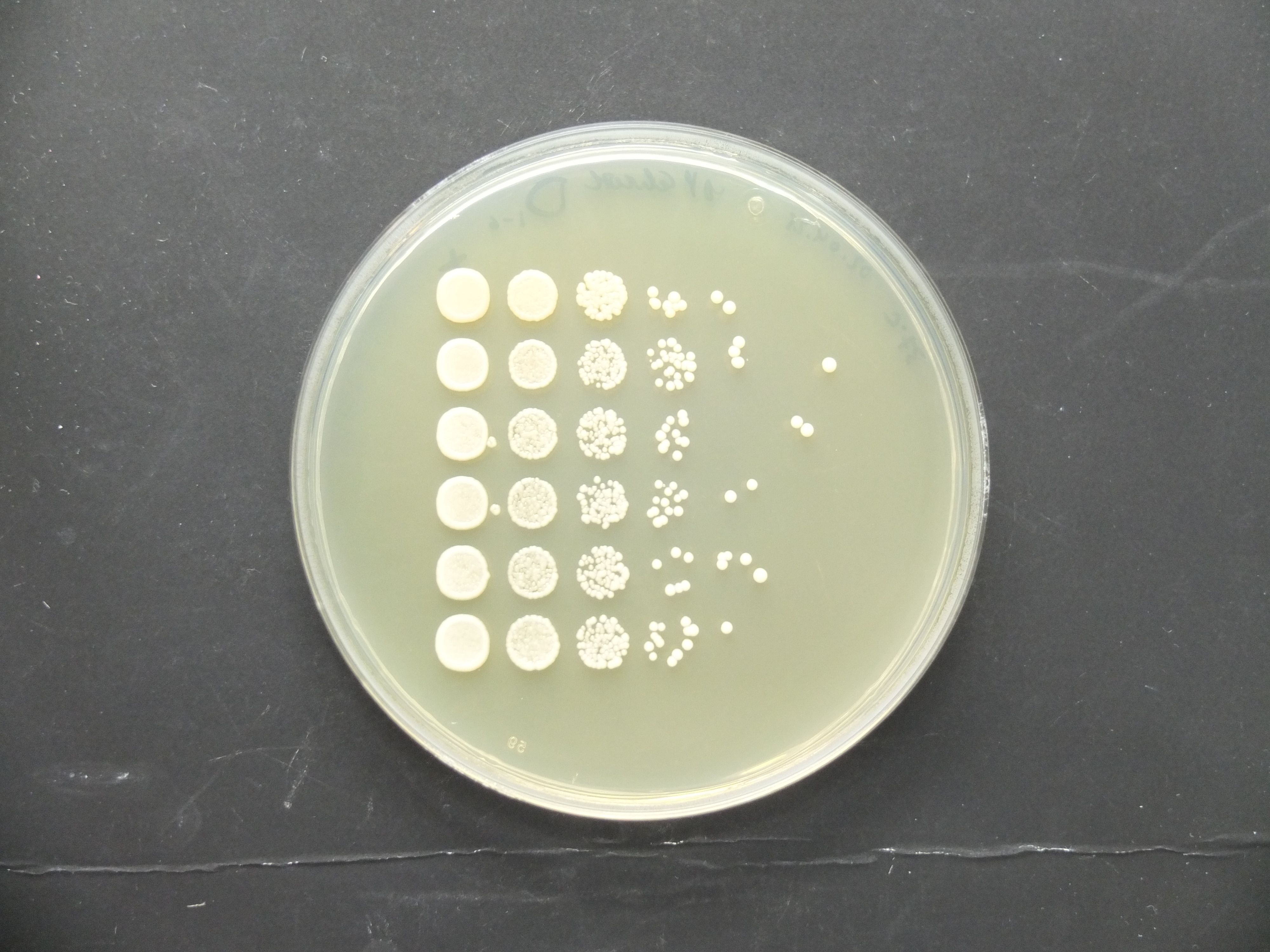

Supplement: Supplementary file 8 — Appendix Figure S6 Source Data [file 44318_2025_459_MOESM8_ESM.zip › Appendix Fig S6/A/right bottom/DSCF8359.JPG]

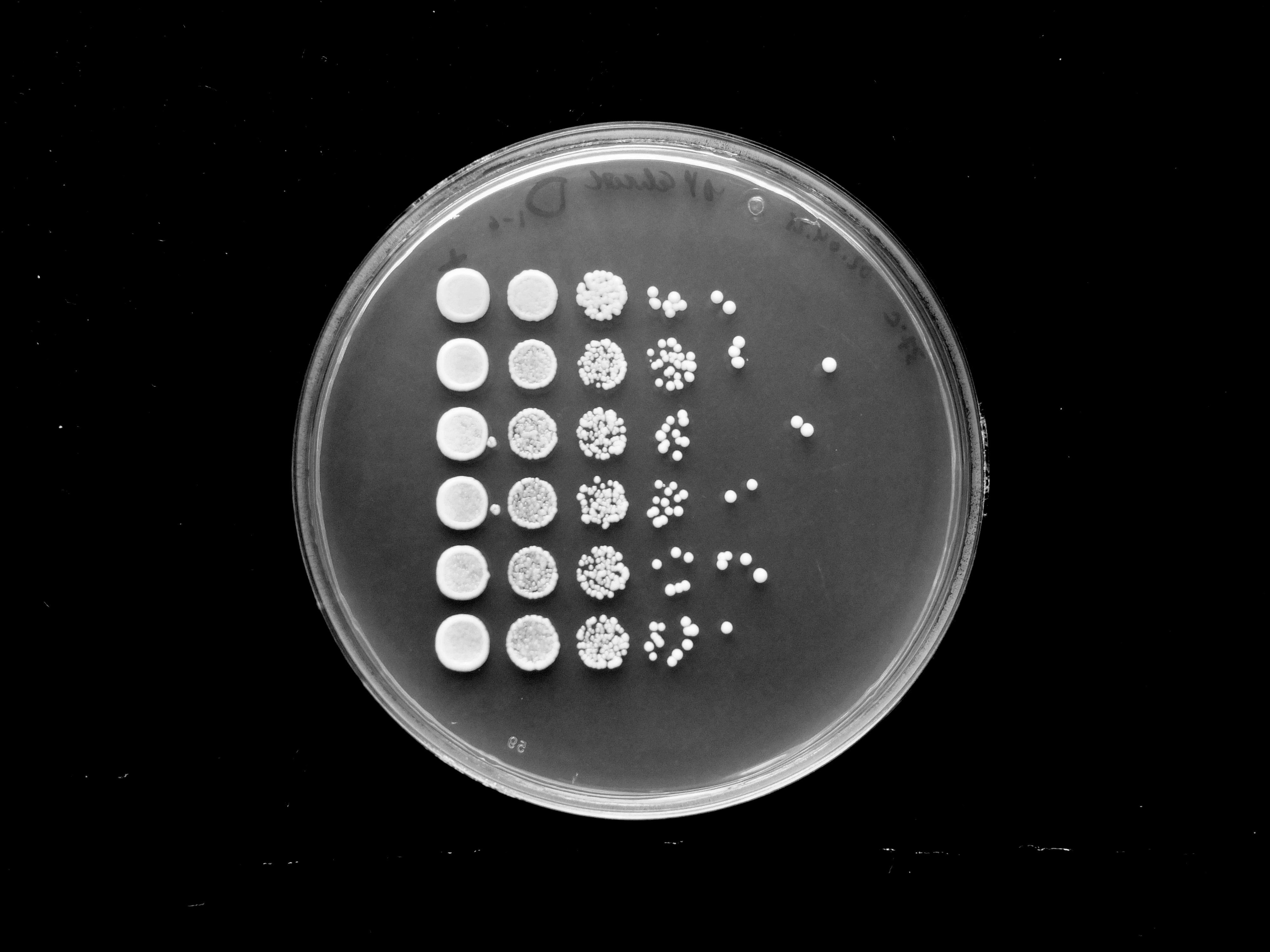

Supplement: Supplementary file 8 — Appendix Figure S6 Source Data [file 44318_2025_459_MOESM8_ESM.zip › Appendix Fig S6/A/right bottom/DSCF8359.tif]

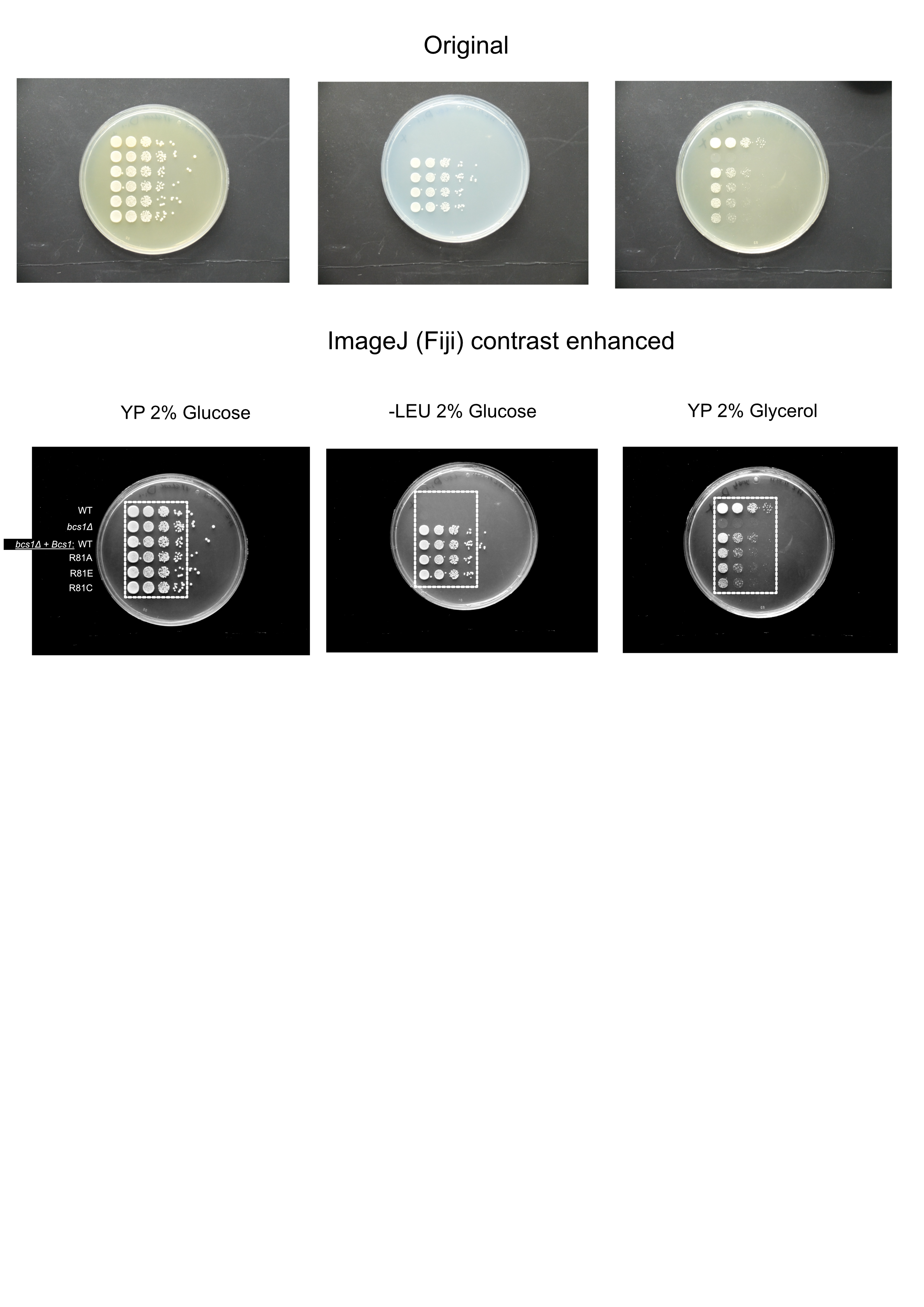

Supplement: Supplementary file 8 — Appendix Figure S6 Source Data [file 44318_2025_459_MOESM8_ESM.zip › Appendix Fig S6/A/right bottom/FigS6Arightbottom_source.png]

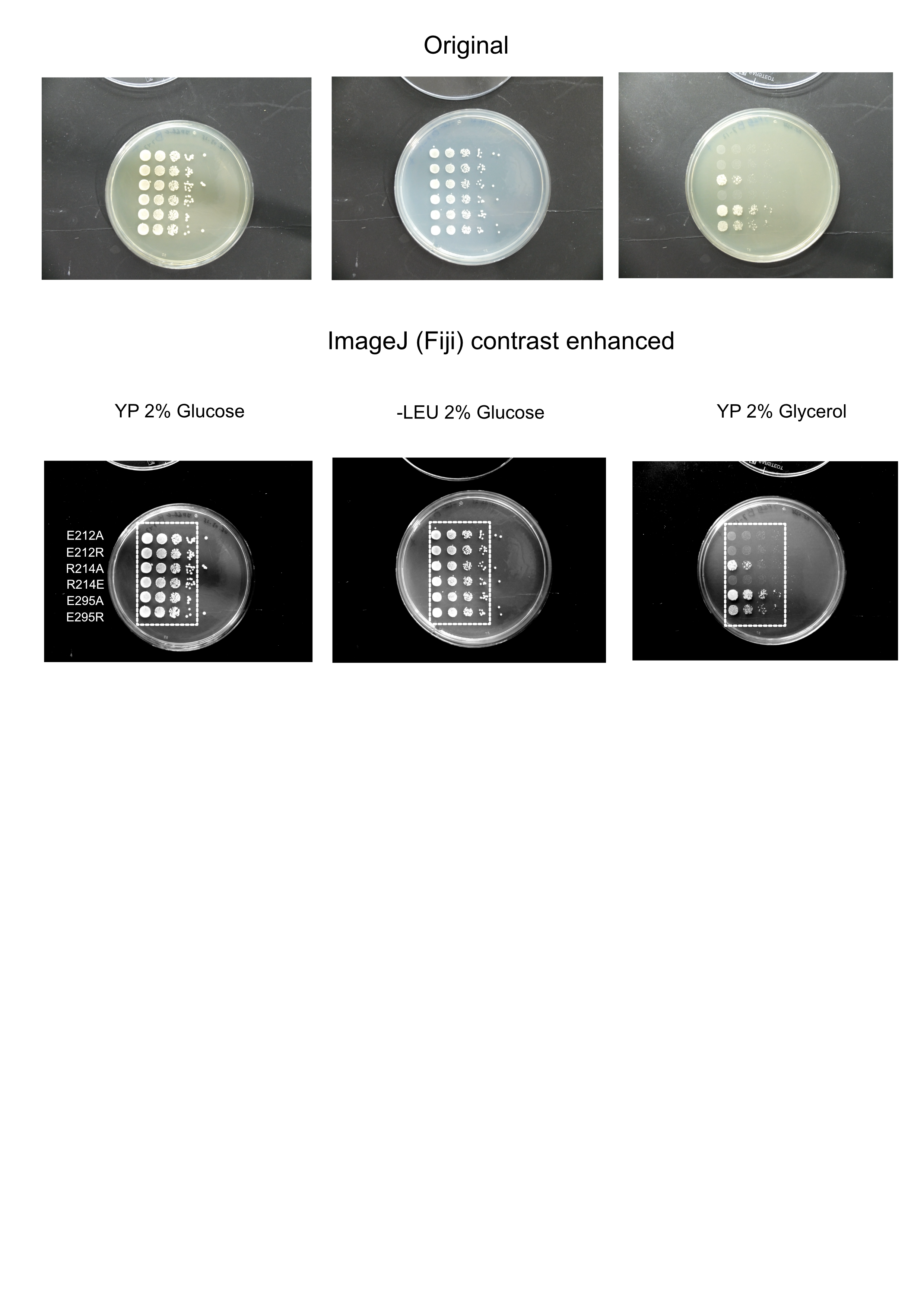

Supplement: Supplementary file 8 — Appendix Figure S6 Source Data [file 44318_2025_459_MOESM8_ESM.zip › Appendix Fig S6/A/left middle/Fig6SAleftmiddle_source.png]

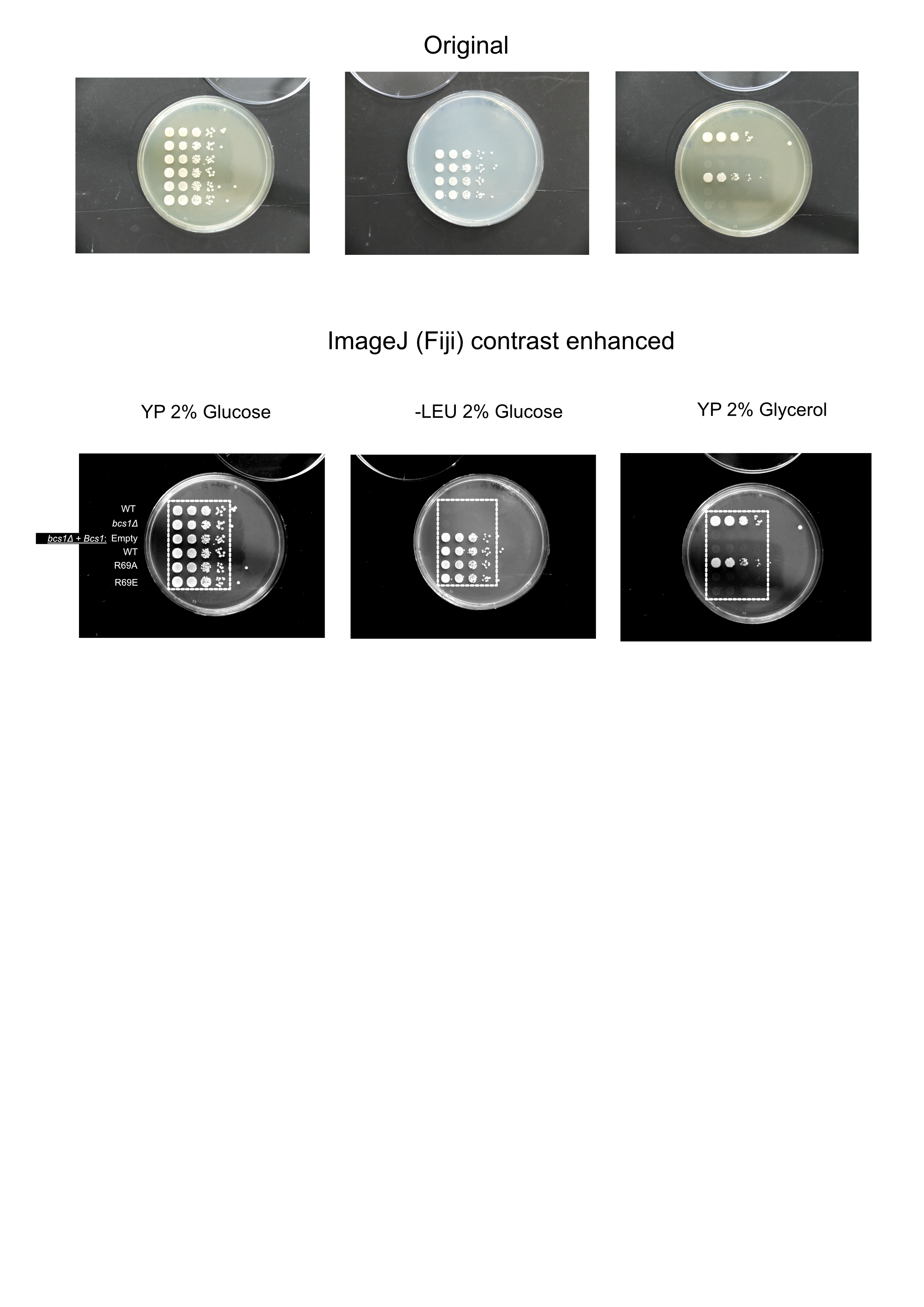

Supplement: Supplementary file 8 — Appendix Figure S6 Source Data [file 44318_2025_459_MOESM8_ESM.zip › Appendix Fig S6/A/left top/FigS6Alefttop_source.png]

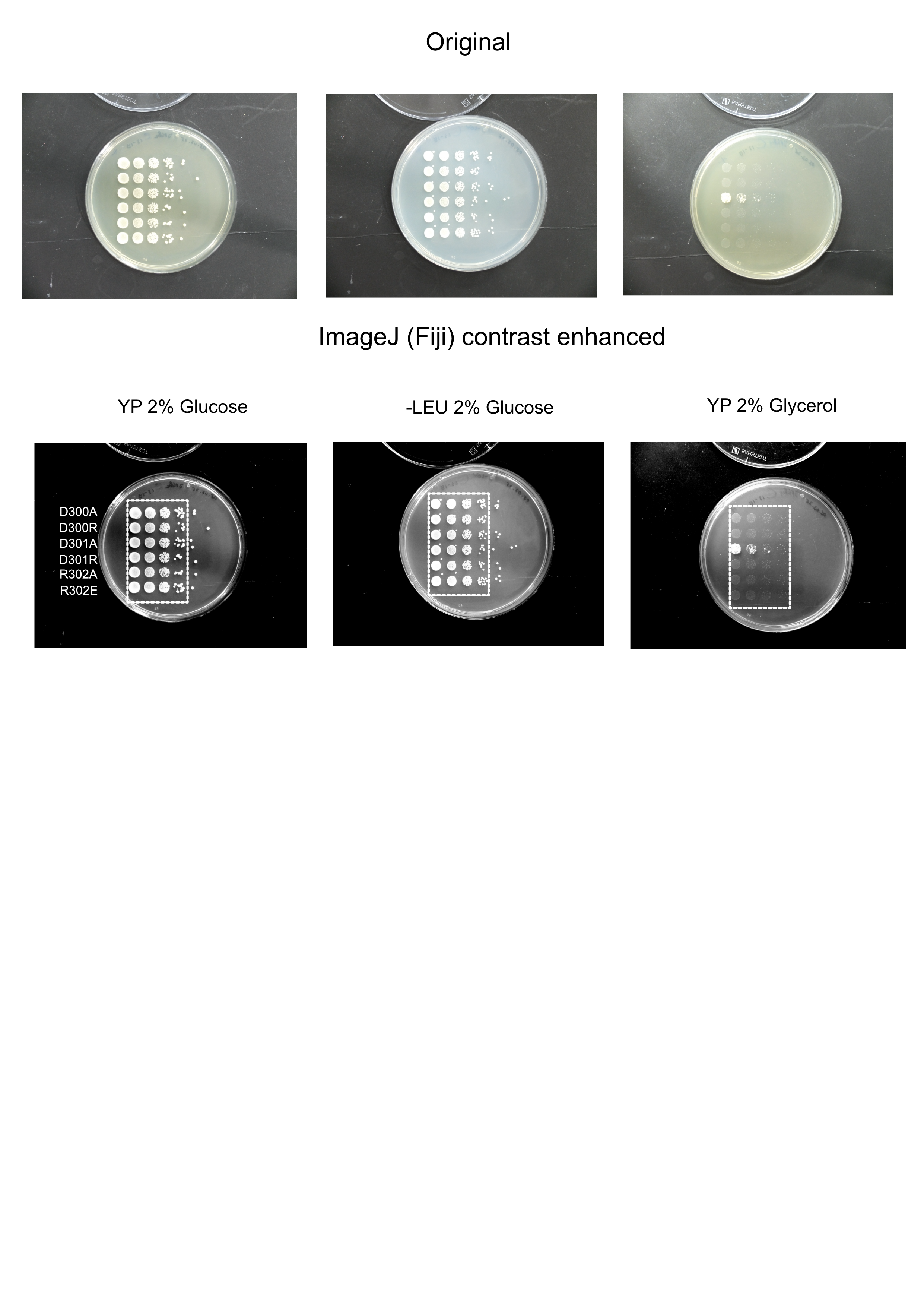

Supplement: Supplementary file 8 — Appendix Figure S6 Source Data [file 44318_2025_459_MOESM8_ESM.zip › Appendix Fig S6/A/left bottom/FigS6Aleftbottom_source.png]

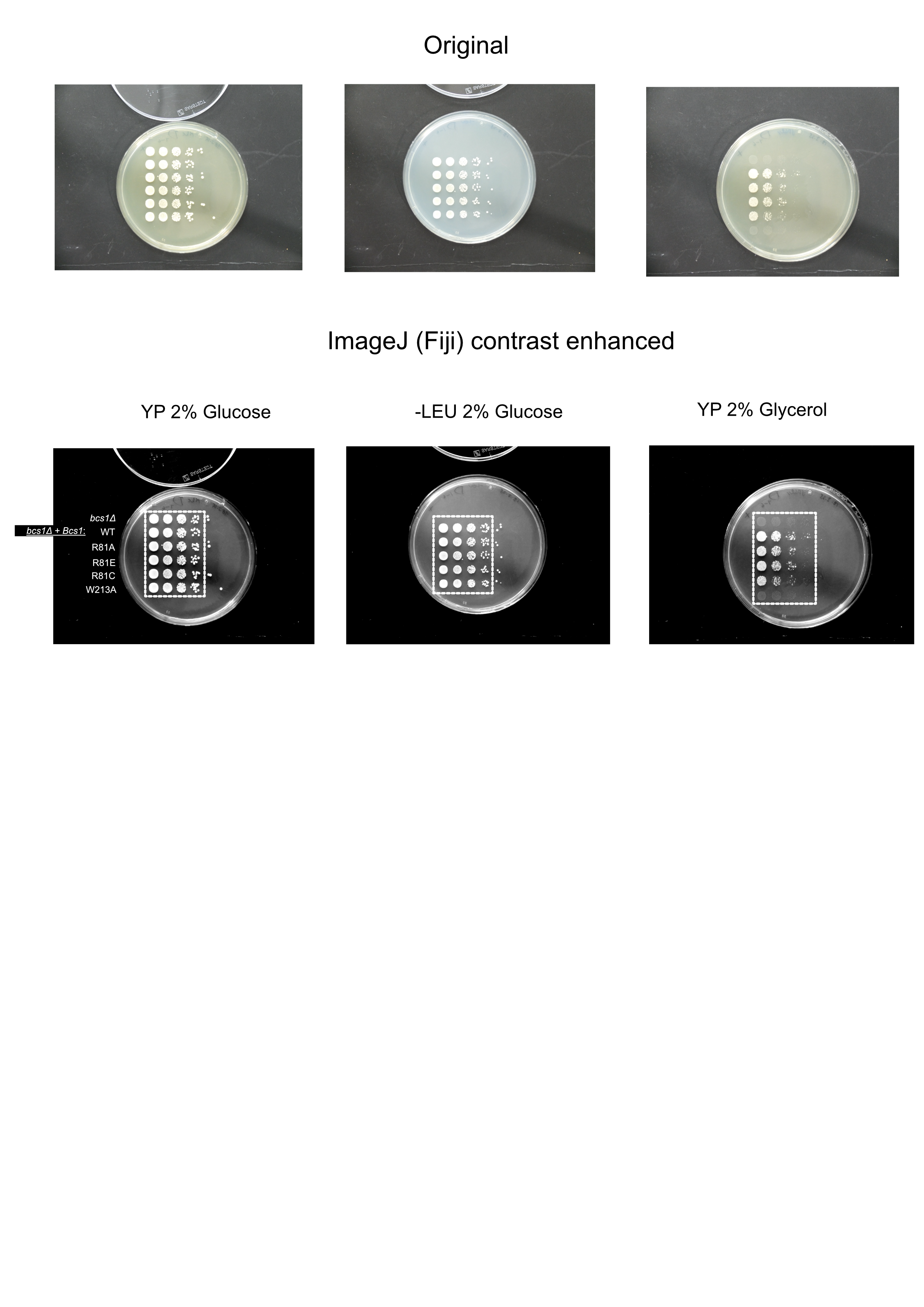

Supplement: Supplementary file 8 — Appendix Figure S6 Source Data [file 44318_2025_459_MOESM8_ESM.zip › Appendix Fig S6/A/right top/FigS6Arighttop_source.png]

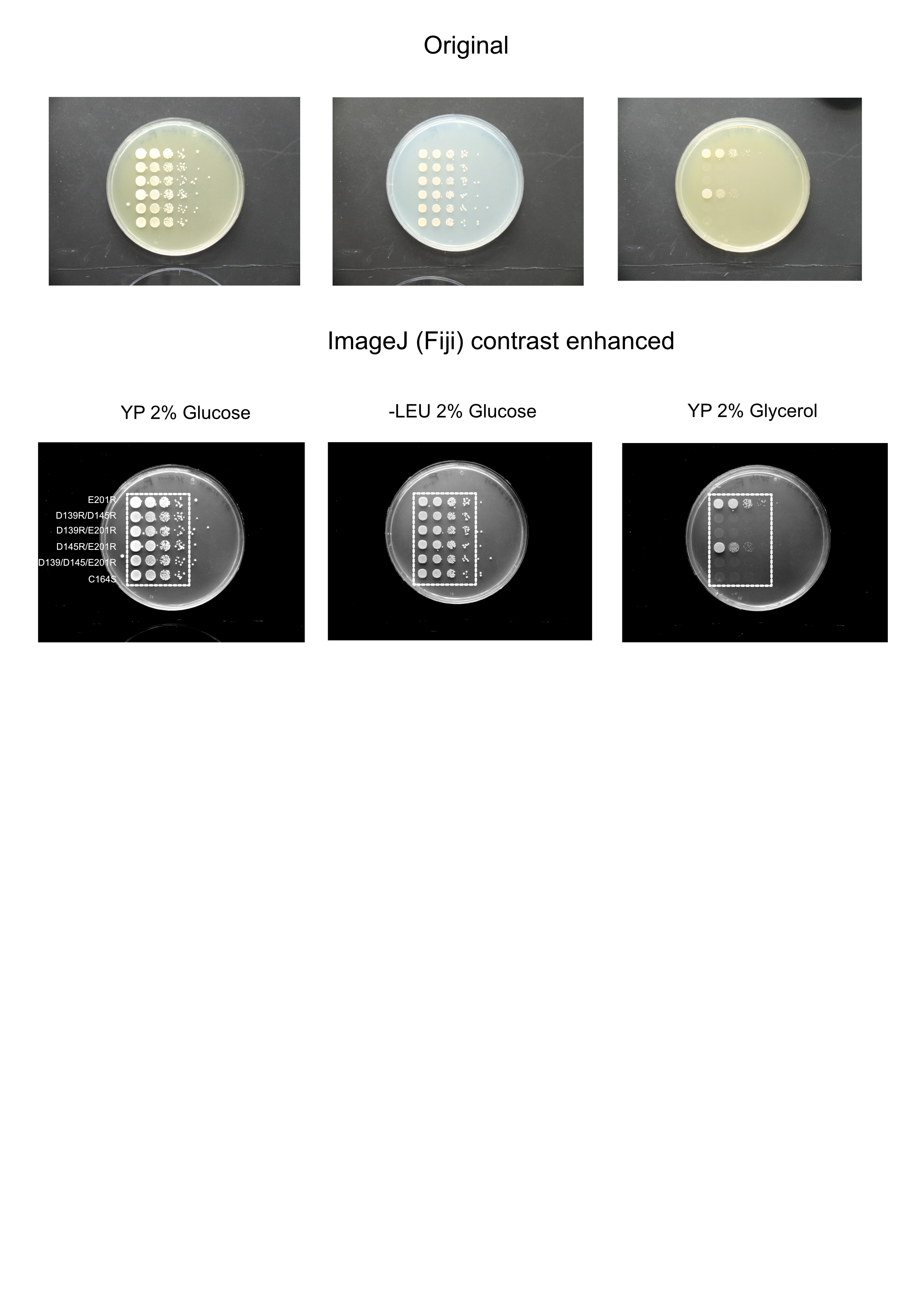

Supplement: Supplementary file 8 — Appendix Figure S6 Source Data [file 44318_2025_459_MOESM8_ESM.zip › Appendix Fig S6/B/right bottom/FigS6Brightbottom_source.png]

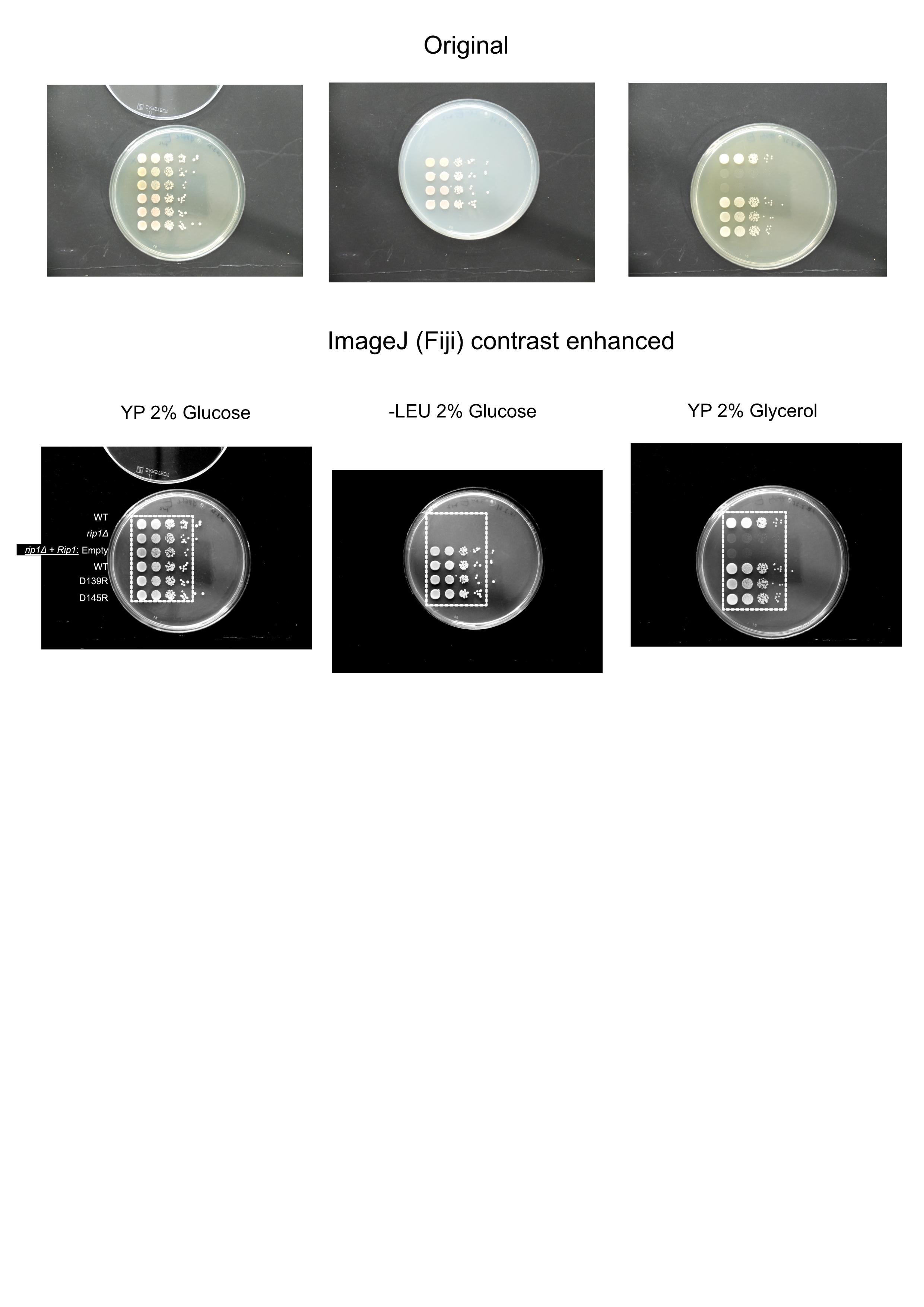

Supplement: Supplementary file 8 — Appendix Figure S6 Source Data [file 44318_2025_459_MOESM8_ESM.zip › Appendix Fig S6/B/left top/FigS6Blefttop_source.png]

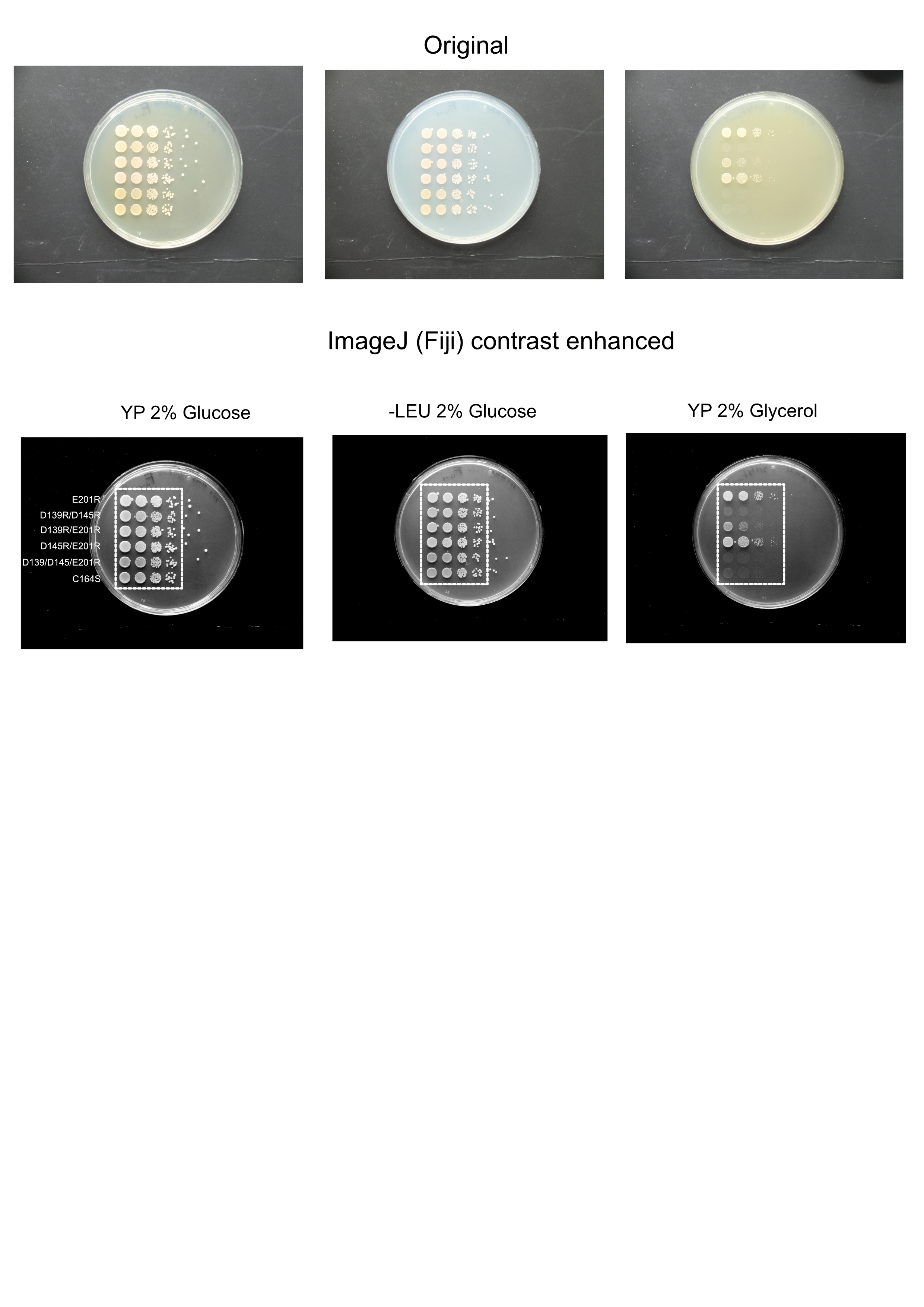

Supplement: Supplementary file 8 — Appendix Figure S6 Source Data [file 44318_2025_459_MOESM8_ESM.zip › Appendix Fig S6/B/left bottom/FigS6Bleftbottom_source.png]

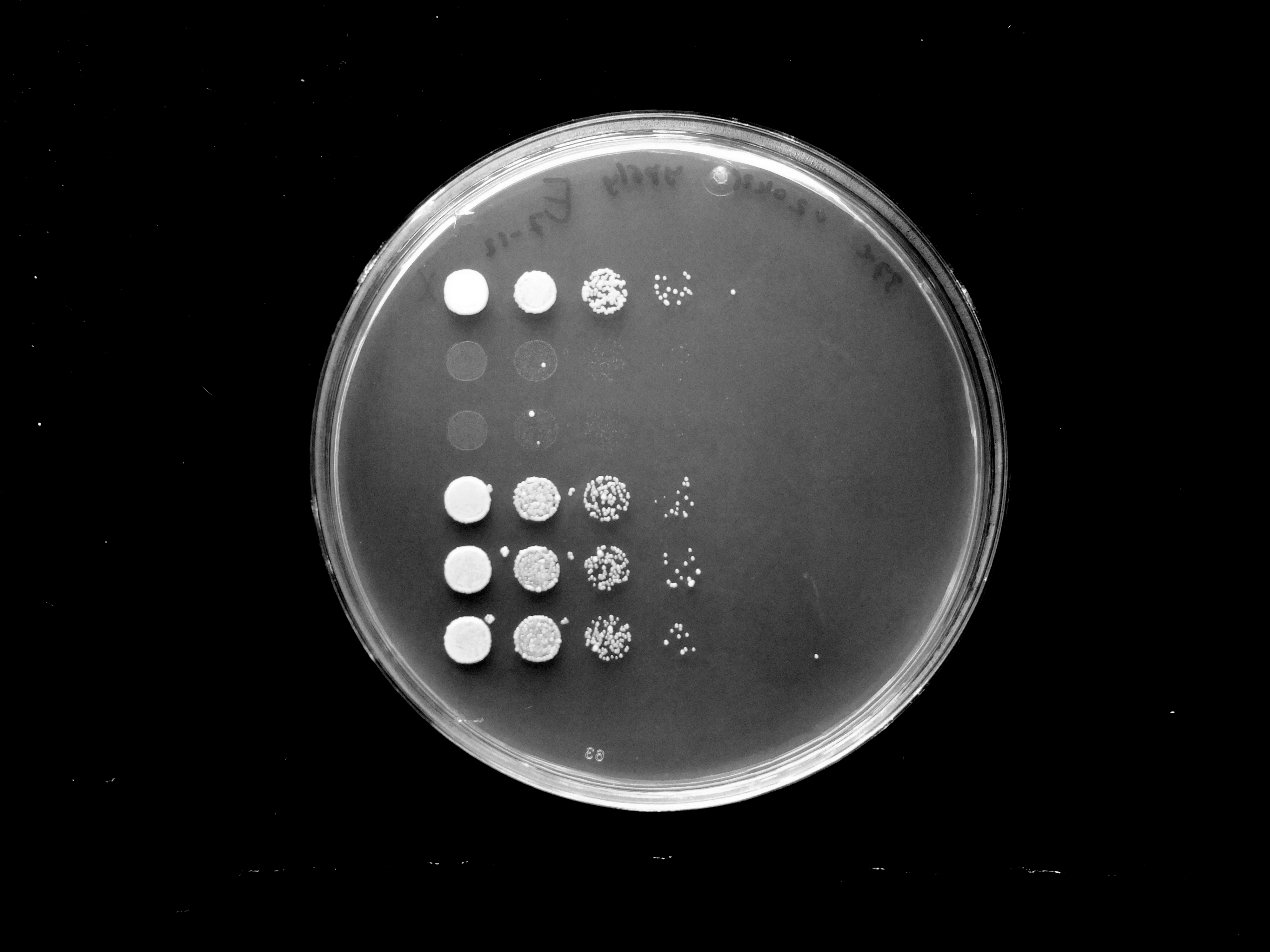

Supplement: Supplementary file 8 — Appendix Figure S6 Source Data [file 44318_2025_459_MOESM8_ESM.zip › Appendix Fig S6/B/right top/DSCF8397.tif]

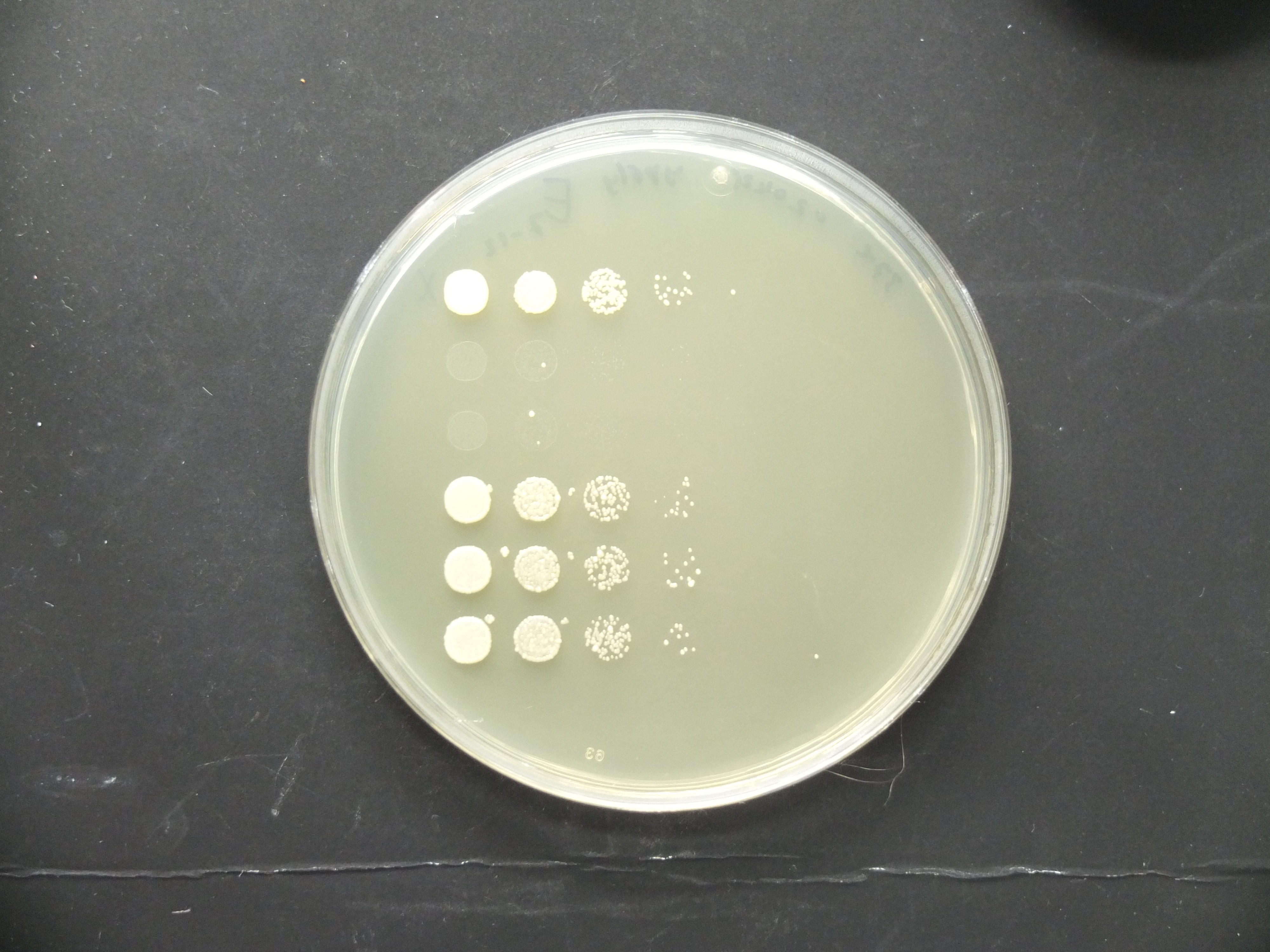

Supplement: Supplementary file 8 — Appendix Figure S6 Source Data [file 44318_2025_459_MOESM8_ESM.zip › Appendix Fig S6/B/right top/DSCF8397.JPG]

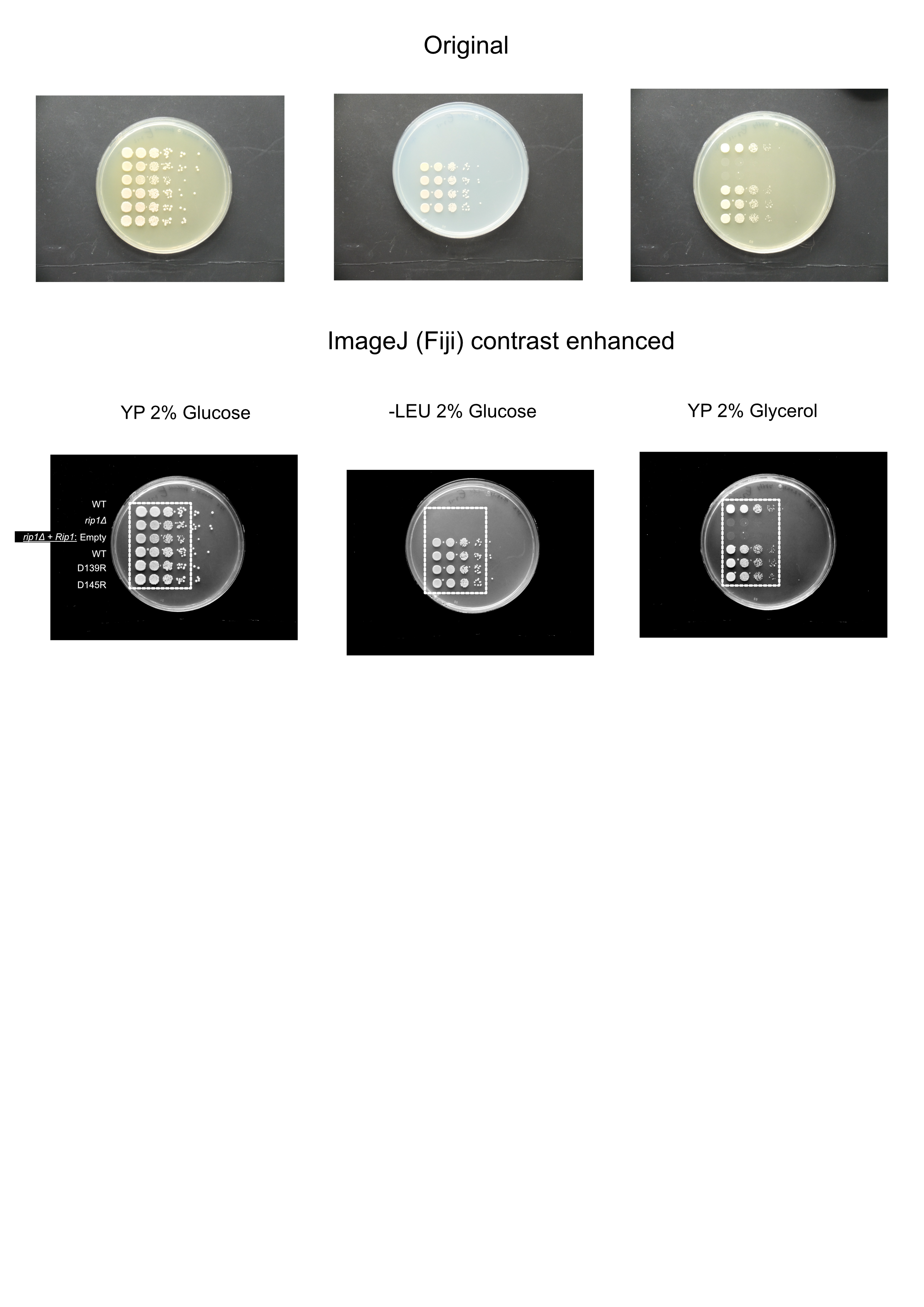

Supplement: Supplementary file 8 — Appendix Figure S6 Source Data [file 44318_2025_459_MOESM8_ESM.zip › Appendix Fig S6/B/right top/FigS6Brighttop_source.png]

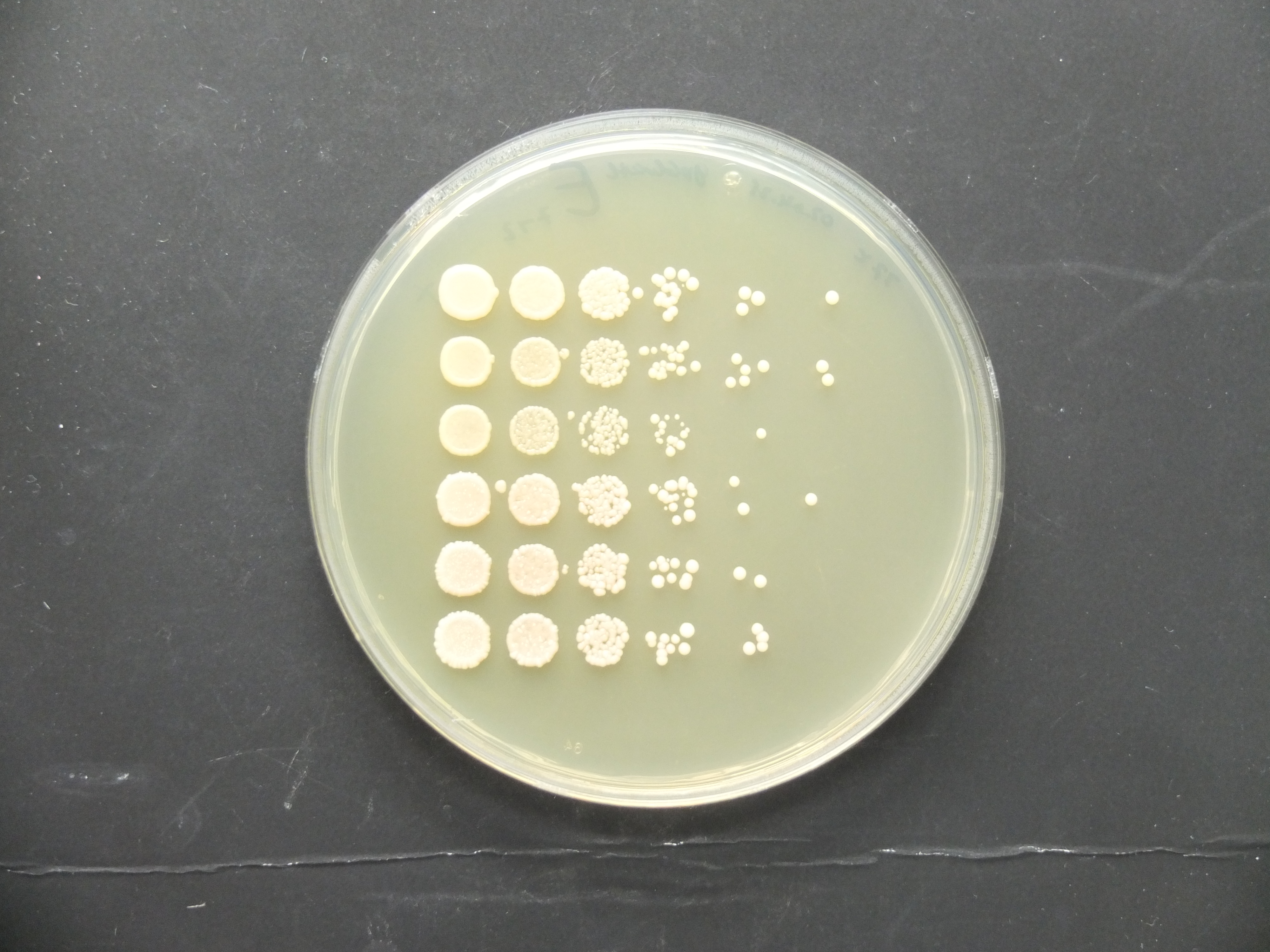

Supplement: Supplementary file 8 — Appendix Figure S6 Source Data [file 44318_2025_459_MOESM8_ESM.zip › Appendix Fig S6/B/right top/DSCF8363.JPG]

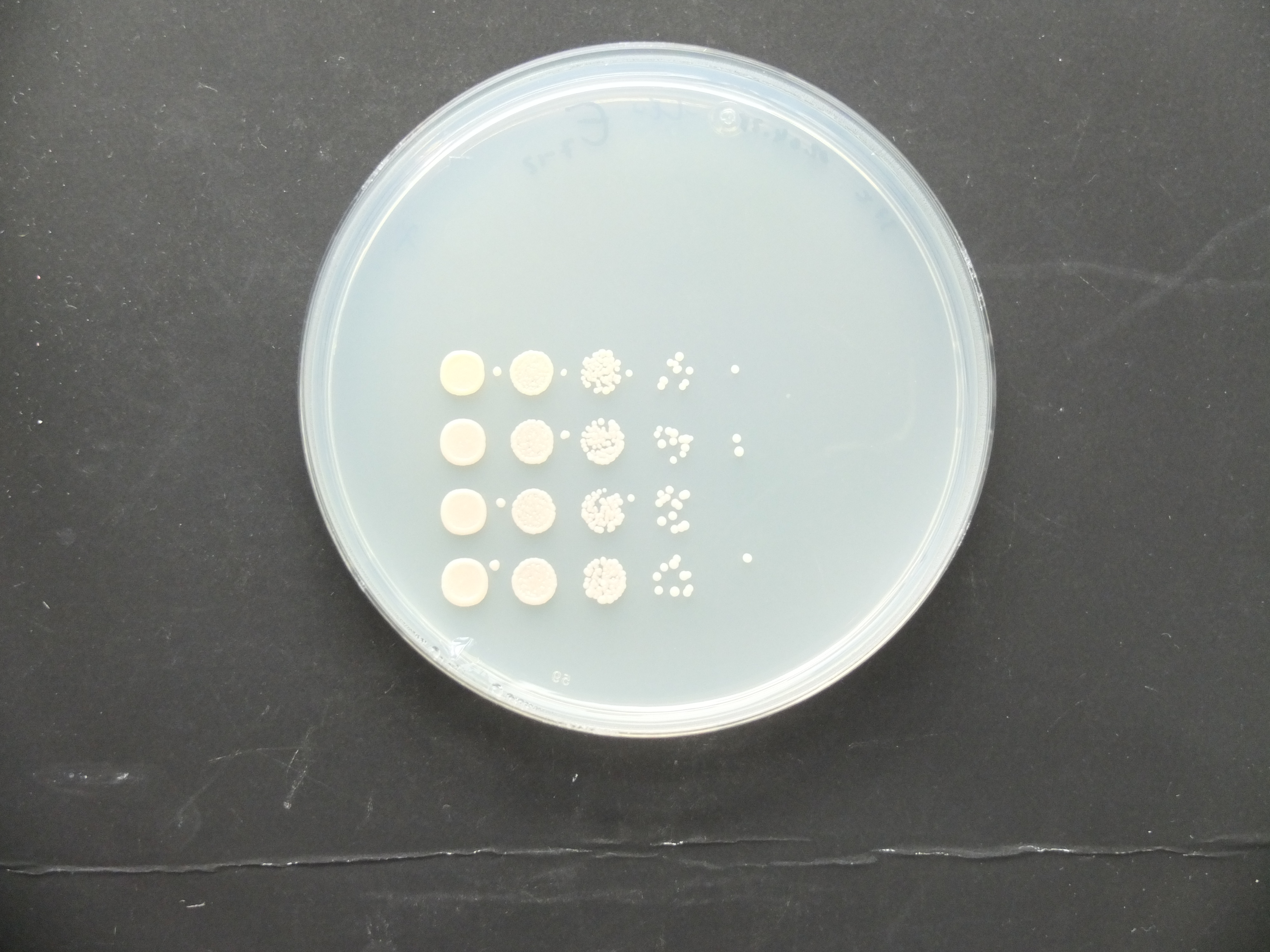

Supplement: Supplementary file 8 — Appendix Figure S6 Source Data [file 44318_2025_459_MOESM8_ESM.zip › Appendix Fig S6/B/right top/DSCF8373.JPG]

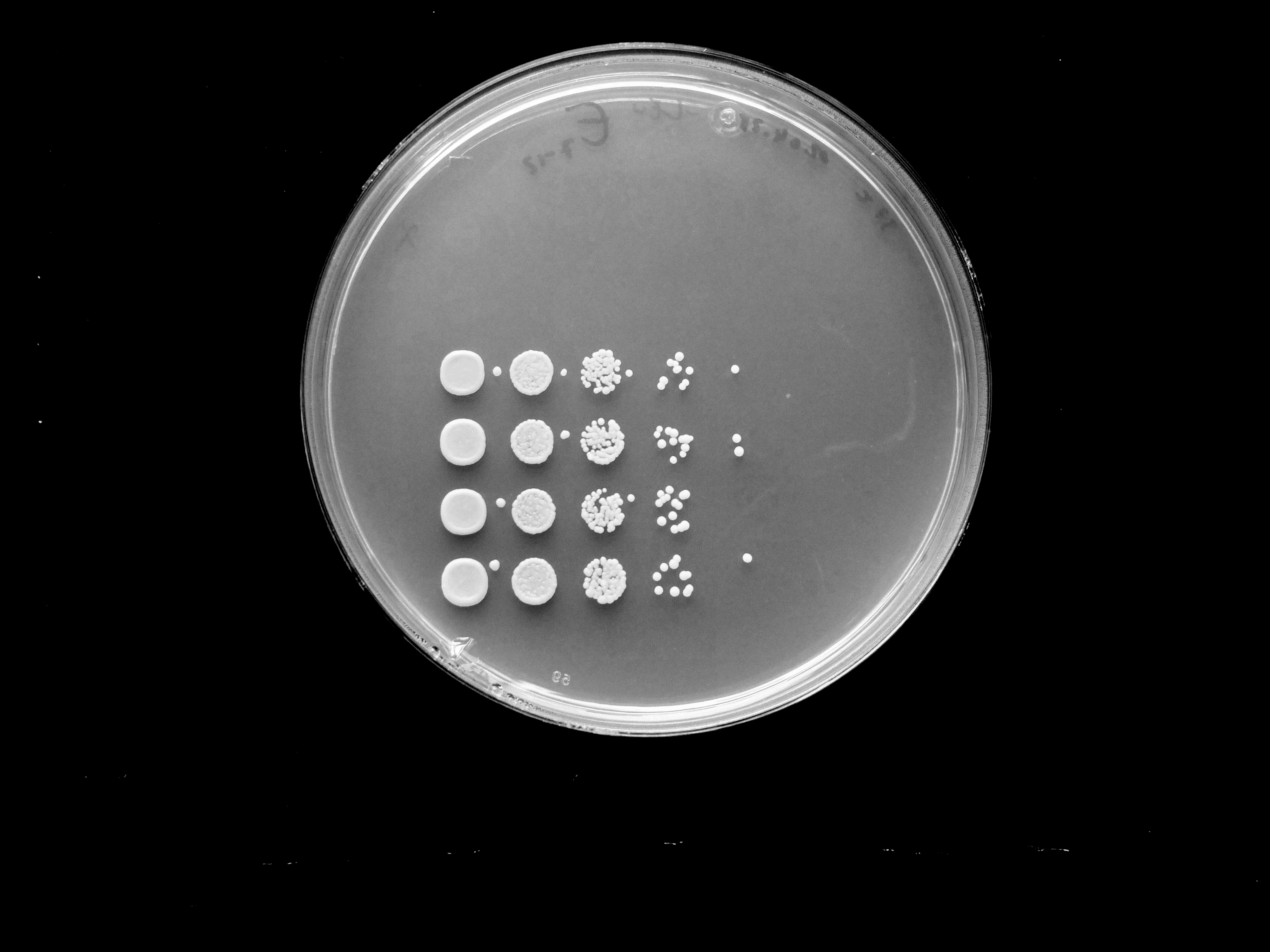

Supplement: Supplementary file 8 — Appendix Figure S6 Source Data [file 44318_2025_459_MOESM8_ESM.zip › Appendix Fig S6/B/right top/DSCF8373.tif]

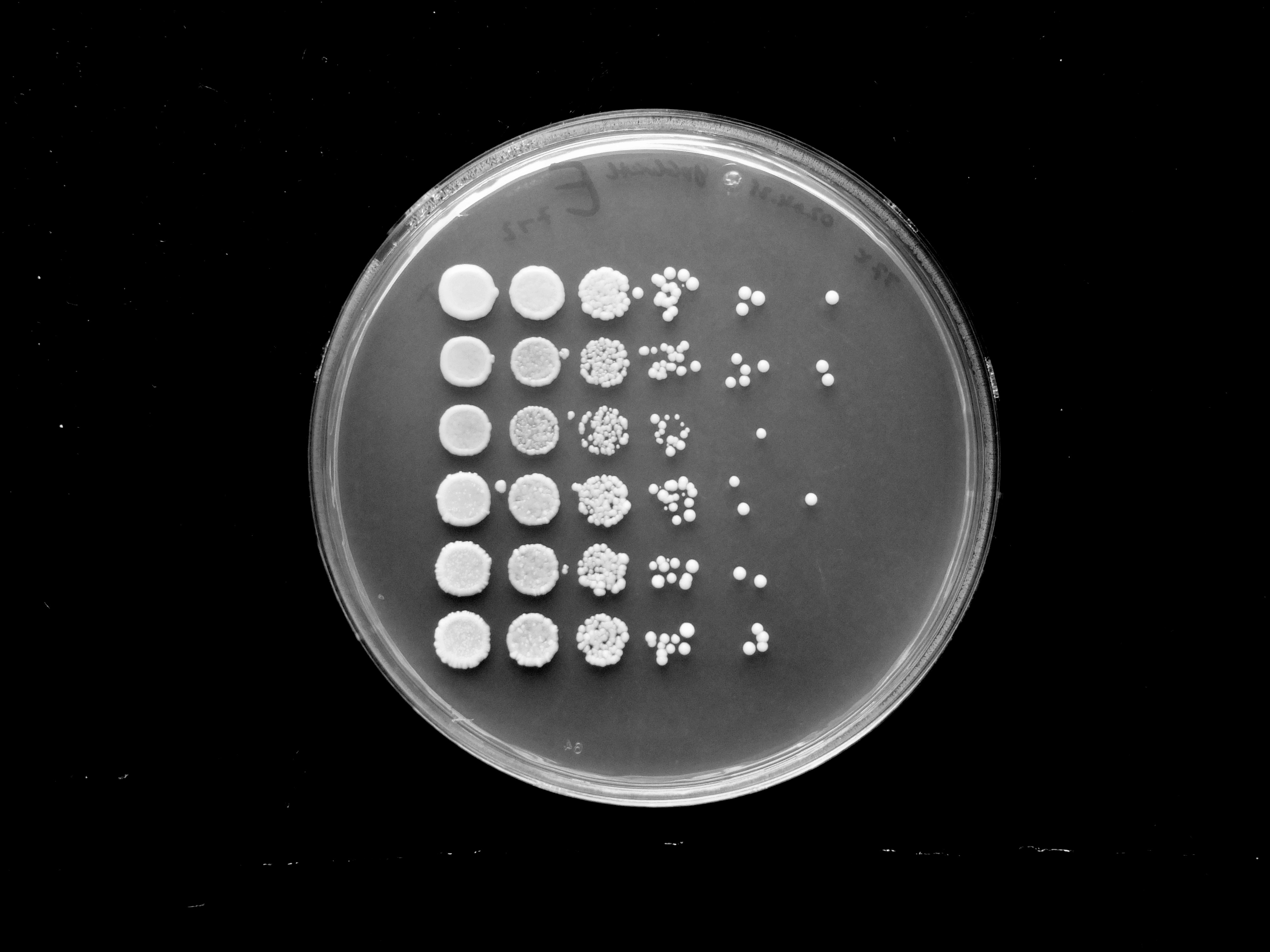

Supplement: Supplementary file 8 — Appendix Figure S6 Source Data [file 44318_2025_459_MOESM8_ESM.zip › Appendix Fig S6/B/right top/DSCF8363.tif]
