# Supplementary material for: Mechanistic insights into Bcs1-mediated mitochondrial membrane translocation of the folded Rieske protein
Source: EMBO J. 2025 May 23;44(13):3720–41. doi: 10.1038/s44318-025-00459-4 (PMC12219900; doi:10.1038/s44318-025-00459-4)
Supplement: Supplementary file 9 — Expanded View Figures [file 44318_2025_459_MOESM9_ESM.pdf]

Expanded View Figures

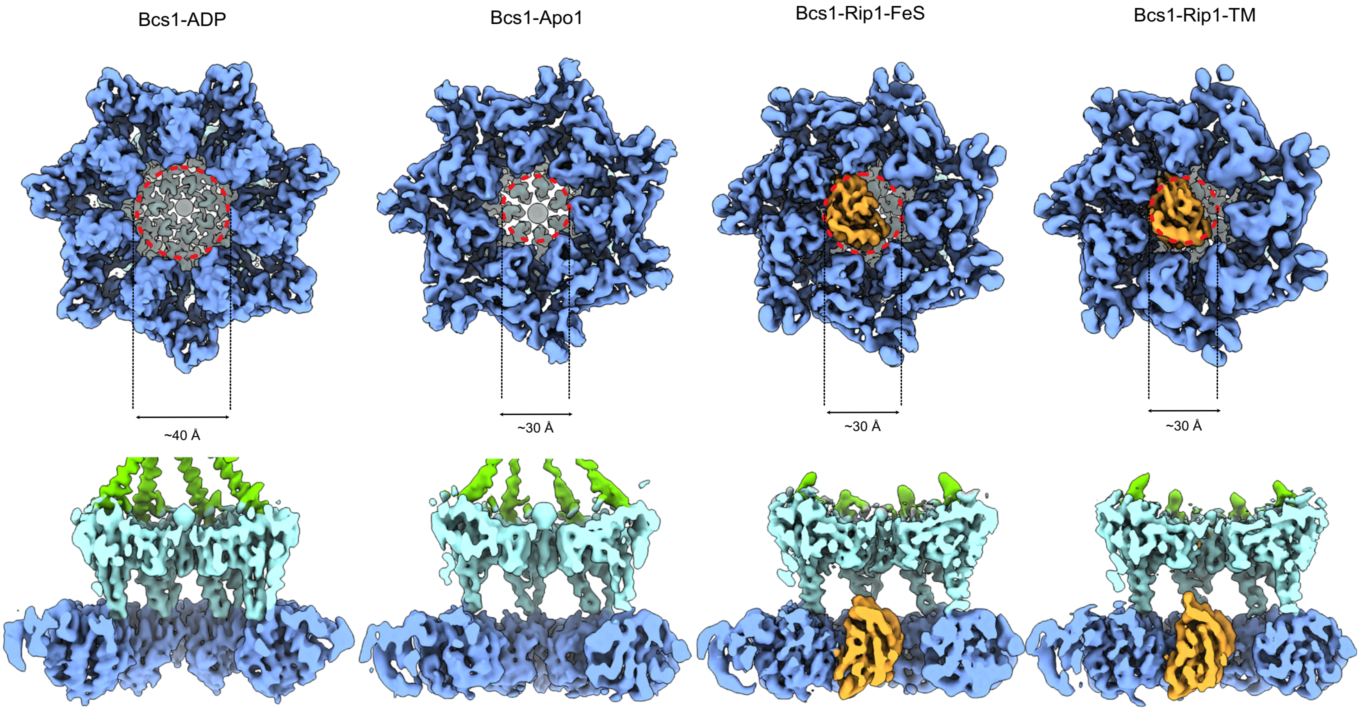

Figure EV1. Comparison of yeast Bcs1 structures in ADP and Apo1 states.

Cryo-EM structures shown at bottom (upper row) and side views (lower row) of Bcs1 in ADP state, Apo1 state (Kater et al, 2020) and bound to Rip1-FeS and Rip1-TM. Note that the Rip1-bound conformation of Bcs1 is Apo1. Red circles represent the diameter of the matrix vestibule.

 © The Author(s)

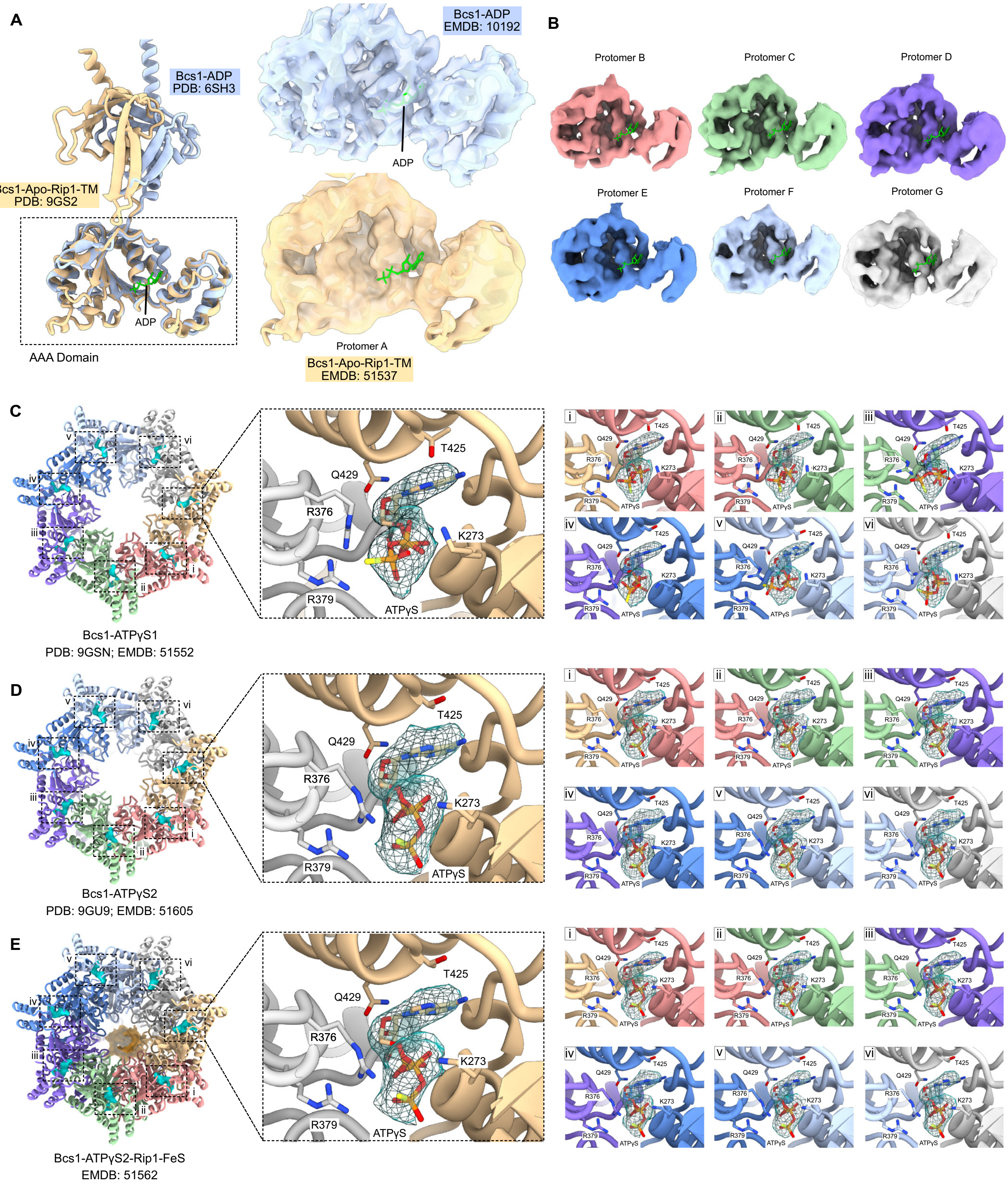
A
Bcs1-ADP
PDB: 6SH3
Bcs1-Apo-Rip1-TM
PDB: 9GS2
ADP
AAA Domain
Bcs1-ADP
EMDB: 10192
ADP
Protomer A
Bcs1-Apo-Rip1-TM
EMDB: 51537
B
Protomer B
Protomer C
Protomer D
Protomer E
Protomer F
Protomer G
C
Bcs1-ATPγS1
PDB: 9GSN; EMDB: 51552
T425
Q429
R376
K273
R379
ATPγS
D
Bcs1-ATPγS2
PDB: 9GU9; EMDB: 51605
E
Bcs1-ATPγS2-Rip1-FeS
EMDB: 51562
i
ii
iii
iv
v
vi

Figure EV2. ATP-binding pocket occupancy in the different Bcs1 states.

(**A**) Left panel: Alignment of the atomic models of Bcs1-ADP (PDB:6SH3, chain A) and Bcs1-Apo1-Rip1-TM (PDB:9GS2, chain A), displaying the relative position of ADP in the binding pocket. Right panel: Close-up view on the AAA domain of protomer A of Bcs1 and map superposition, highlighting the density of ADP found in the Bcs1-ADP state (top) and absent in Bcs1-Apo1-Rip1-TM (bottom). (**B**) ADP nucleotide model superimposed on the AAA domain density of the protomers B-G of Bcs1-Apo1-Rip1-TM. (**C**) Left panel: Bottom view of Bcs1- ATPγS1 and isolated ATPγS density. Highlighted in squares are a close-up view of the density in the protomer A and, in boxes i-vi, ATPγS densities for protomers B-G. Right panel: Close-up views of the enclosed regions i-vi. (**D**) same as in C for Bcs1- ATPγS2. (**E**) Same as in (**C**) for Bcs1- ATPγS2-Rip1-FeS.

 © The Author(s)

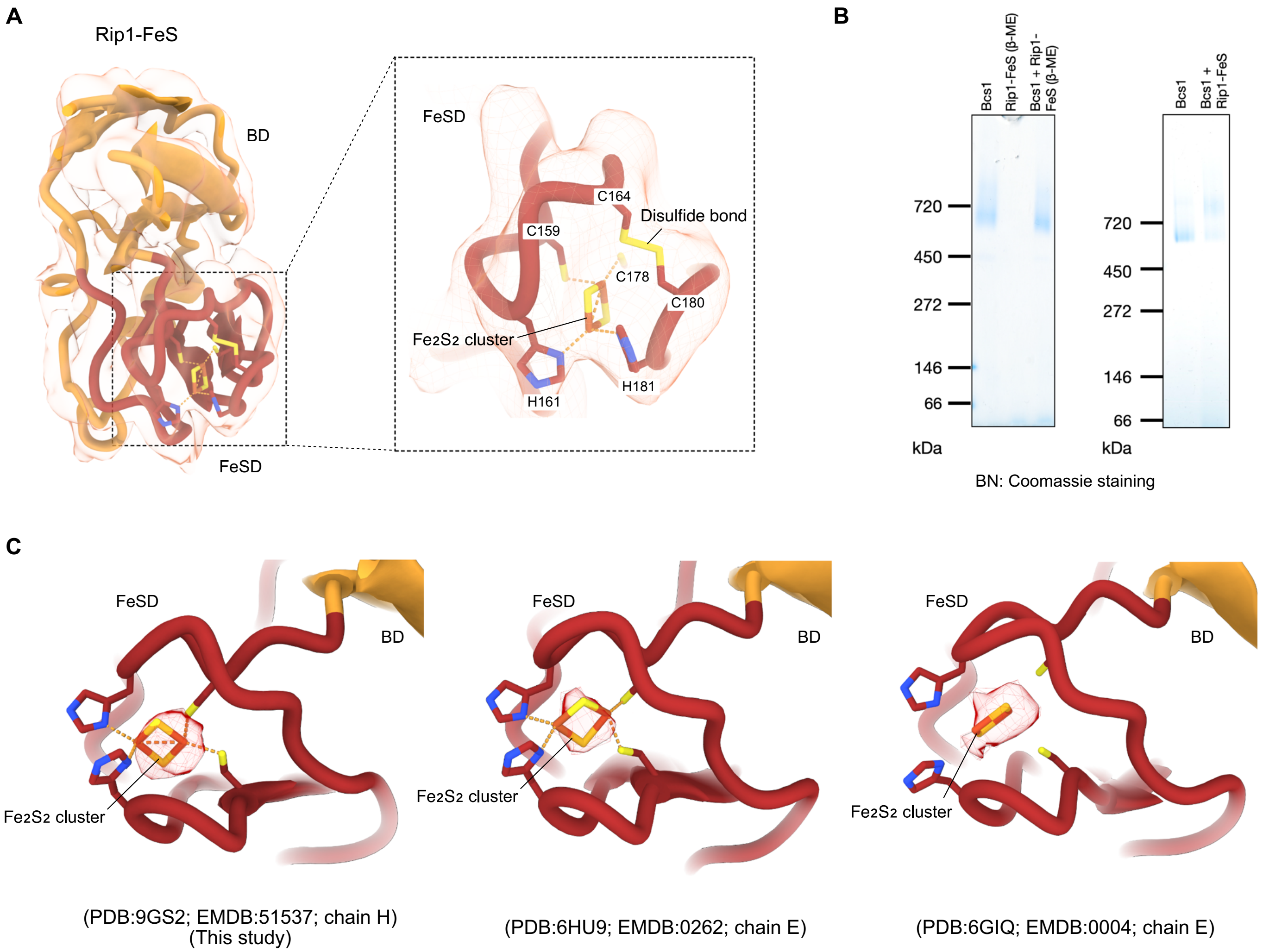

Figure EV3. Structural details of Rip1-FeS bound to Bcs1 in the Apo1 state.

(**A**) Left panel: Rip1-FeS atomic model fitted into density, highlighting the FeSD domain. Right panel: Close-up view on the FeSD displaying the residues involved in coordination of the 2Fe–2S and the disulfide bond between C164 and C180. The 2Fe–2S cluster is shown in a stick representation, where yellow sticks represent the sulphur atoms and red sticks represent the iron atoms. (**B**) Blue Native (BN) gel showing the effect of β-mercaptoethanol (β-ME) on the binding of purified Rip1-FeS to Bcs1 (left) compared to Rip1-FeS purified in the absence of β-ME. (**C**) Comparison of the Rip1-associated 2Fe–2S cluster densities from Bcs1-Apo1-Rip1-TM and active CIII complex Cryo-EM maps. Source data are available online for this figure.

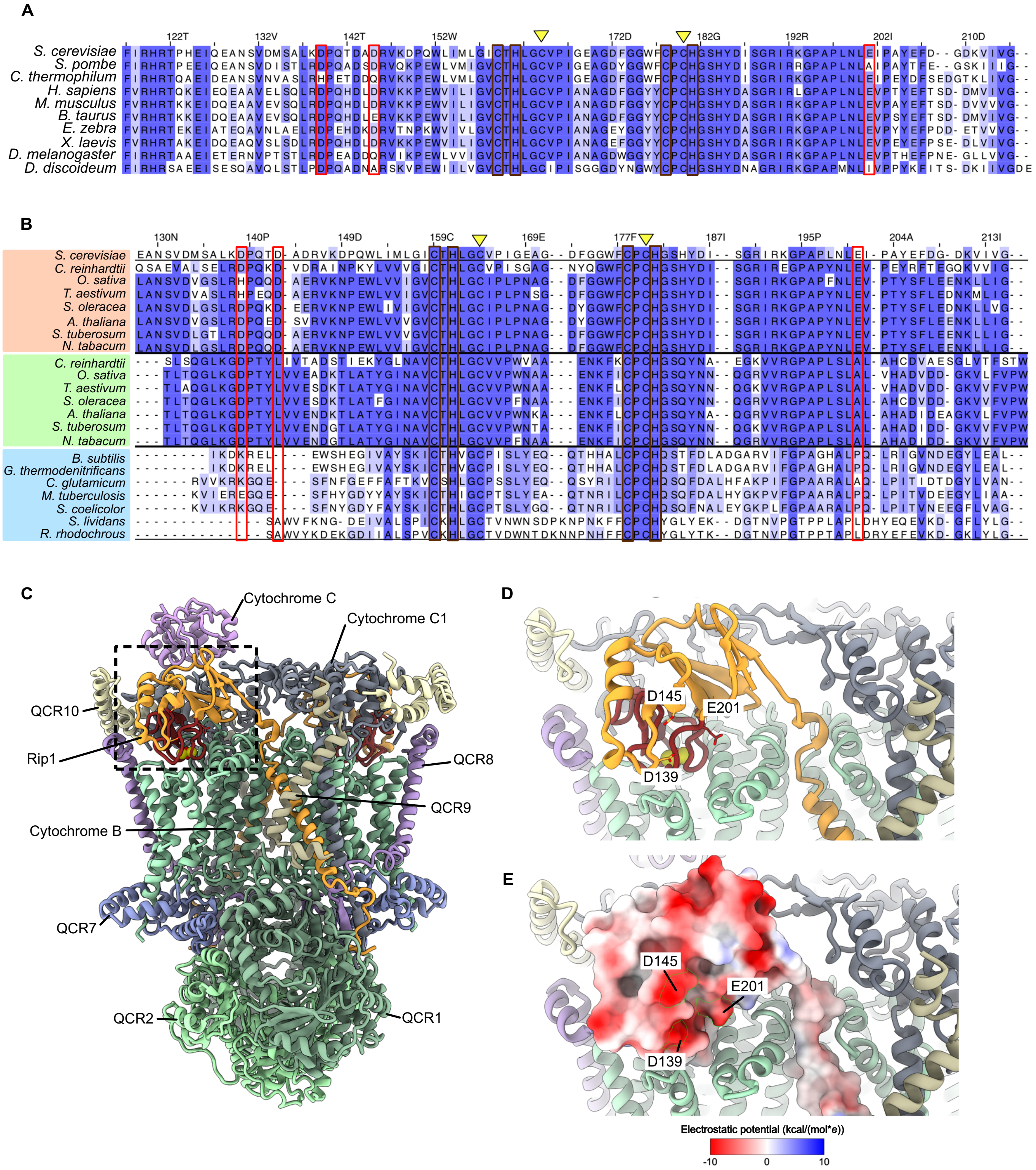
A
122T
132V
142T
152W
172D
182G
192R
202I
210D
S. cerevisiae
S. pombe
C. thermophilum
H. sapiens
M. musculus
B. taurus
E. zebra
X. laevis
D. melanogaster
D. discoideum
B
130N
140P
149D
159C
169E
177F
187I
195P
204A
213I
S. cerevisiae
C. reinhardtii
O. sativa
T. aestivum
S. oleracea
A. thaliana
S. tuberosum
N. tabacum
C. reinhardtii
O. sativa
T. aestivum
S. oleracea
A. thaliana
S. tuberosum
N. tabacum
B. subtilis
G. thermodenitrificans
C. glutamicum
M. tuberculosis
S. coelicolor
S. lividans
R. rhodochrous
C
Cytochrome C
Cytochrome C1
QCR10
Rip1
QCR8
QCR9
Cytochrome B
QCR7
QCR2
QCR1
D
D145
E201
D139
E
D145
E201
D139
Electrostatic potential (kcal/(mol*e))
-10
0
10

 © The Author(s)

Figure EV4. Conserved residues on the Bcs1-interaction surface of Rip1.

(**A**) Multiple sequence alignment of the globular domain of Rip1 from selected species generated by Clustal Omega (Madeira et al, 2024) and displayed using Jalview (Waterhouse et al, 2009). Enclosed in red boxes are some of the negatively charged residues (D139, D145, E201) that interact with Bcs1 during translocation, in brown boxes are the residues responsible for the coordination of the 2Fe–2S cluster (C159, H161, C178, H181) and yellow arrows point to the residues that form a structurally relevant disulfide bond (C164, C180). The amino acids are colored according to their percentage of identity. (**B**) Multiple sequence alignment as in (**A**) but comparing the mitochondrial (pink box) Rip1 homolog of photosynthetic species with its chloroplast homolog petC in the same species (green box) and its prokaryotic homolog qcrA from prokaryote species (light blue box). (**C**) Structure of the fully assembled dimeric bc1 complex (PDB: 1KYO), highlighting the position of the globular domain of one of the two Rip1 subunits. (**D**) Close-up view on the enclosed region from (**C**), displaying Rip1 negatively charged amino acids. (**E**) Same view as in (**D**) but displaying the negatively charged electrostatic surface. The 2Fe–2S cluster is shown in a sphere representation, where yellow spheres represent the sulfur atoms and red spheres represent the iron atoms.

© The Author(s)

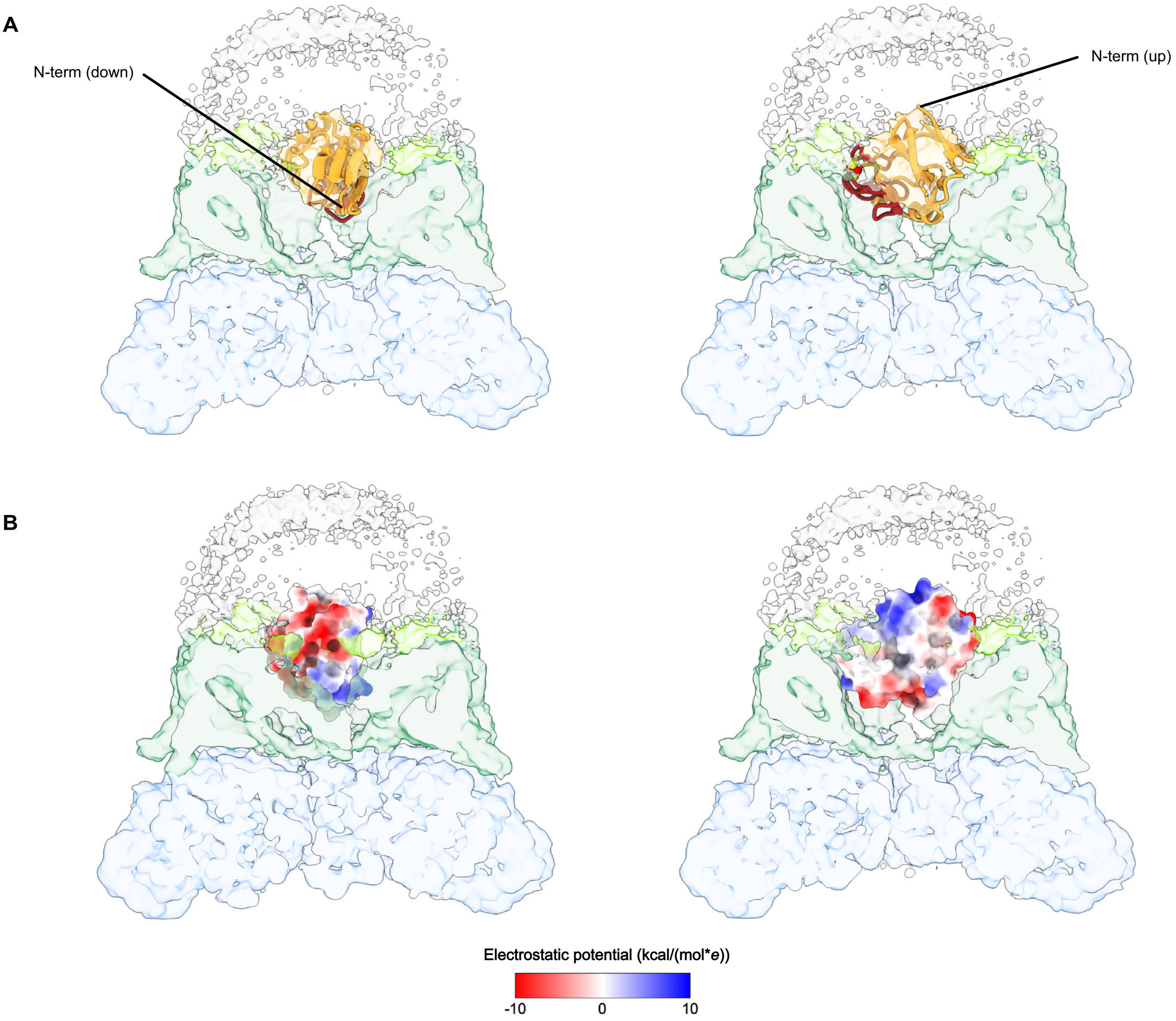

Figure EV5. Plausible orientations of Rip1-FeS in the translocation state.

(**A**) Cut side view of the atomic model of Rip1-FeS docked into the Bcs1-ATPγS2-Rip1-FeS map in an orientation that locates the N-terminal residues towards the mitochondrial matrix (left) or towards the IMS (right). The 2Fe–2S cluster is shown in a sphere representation, where yellow spheres represent the sulfur atoms and red spheres represent the iron atoms. (**B**) Electrostatic surface representation of the models displayed in (**A**).

 © The Author(s)
